# Asymmetric Lewis acid catalysis directed by octahedral rhodium centrochirality†
†Electronic supplementary information (ESI) available: Experimental details and analytical data, including chiral HPLC traces and X-ray crystallographic data. CCDC 1027144–1027147, 1028075 and 1014508. For ESI and crystallographic data in CIF or other electronic format see DOI: 10.1039/c4sc03101f



**DOI:** 10.1039/c4sc03101f

**Published:** 2014-11-10

**Authors:** Chuanyong Wang, Liang-An Chen, Haohua Huo, Xiaodong Shen, Klaus Harms, Lei Gong, Eric Meggers

**Affiliations:** a Fachbereich Chemie , Philipps-Universität Marburg , Hans-Meerwein-Straße , 35043 Marburg , Germany . Email: meggers@chemie.uni-marburg.de; b Department of Chemical Biology and Key Laboratory for Chemical Biology of Fujian Province , College of Chemistry and Chemical Engineering , Xiamen University , Xiamen 361005 , People's Republic of China

## Abstract

A rhodium-based asymmetric catalyst is introduced which derives its optical activity from octahedral centrochirality. Besides providing the exclusive source of chirality, the rhodium center serves as a Lewis acid by activating 2-acyl imidazoles through two point binding and enabling a very effective asymmetric induction mediated by the propeller-like *C*_2_-symmetrical ligand sphere. Applications to asymmetric Michael additions (electrophile activation) as well as asymmetric α-aminations (nucleophile activation) are disclosed, for which the rhodium catalyst is found to be overall superior to its iridium congener. Due to its straightforward proline-mediated synthesis, high catalytic activity (catalyst loadings down to 0.1 mol%), and tolerance towards moisture and air, this novel class of chiral-at-rhodium catalysts will likely to become of widespread use as chiral Lewis acid catalysts for a large variety of asymmetric transformations.

## Abstract 2

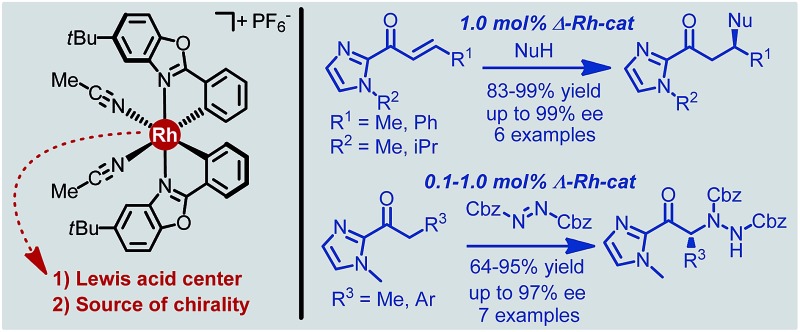
A chiral-at-metal octahedral rhodium(iii) complex serves as an effective asymmetric catalyst for Michael additions (electrophile activation) and α-aminations (nucleophile activation).

## 

Lewis acids are capable of activating a large variety of carbon-heteroatom and carbon–carbon bond forming reactions and chiral Lewis acids have therefore become indispensable tools for asymmetric catalysis.^1^ Their canonical design consists of a central metal ion coordinated to chiral organic ligands so that one-point or two-point binding of a substrate to the Lewis acidic metal ion activates the substrate towards nucleophilic or electrophilic attack by a co-substrate or reagent and at the same time provides the mode of asymmetric induction by transfering chirality from the organic ligands to the product, typically through shielding one face of a prochiral center. However, such chiral Lewis acids, although proven extremely useful and often prepared in a straightforward fashion by *in situ* combining a chiral ligand with an inorganic salt, have some intrinsic limitations resulting from possible background reactions of the non-coordinated metal salt combined with the phenomenon of ligand decelerated catalysis.^2^

Recently, we introduced a chiral-at-metal iridium(iii) complex as novel type of chiral Lewis acid in which the octahedral iridium center is irreversibly cyclometalated by two achiral bidentate ligands in a propeller type fashion and thereby provides the sole source of chirality (Δ-**Ir** in Fig. 1).^3,4^ We here now wish to report for the first time that rhodium can also serve as the combined source of centrochirality and Lewis acidity in substitutionally labile octahedral metal complexes. Unexpectedly, despite the well established significantly higher coordinative lability of rhodium(iii) over iridium(iii), the substitutionally labile, reactive rhodium catalyst retains its absolute and relative configuration in solution over many days without any signs of isomerisation. Importantly, for most of the investigated transformations, the rhodium catalyst is superior to its isostructural iridium congener, which can at least in parts be attributed to the faster ligand exchange kinetic of the rhodium complex, permitting higher turnover frequencies and turnover numbers.

**Fig. 1:**
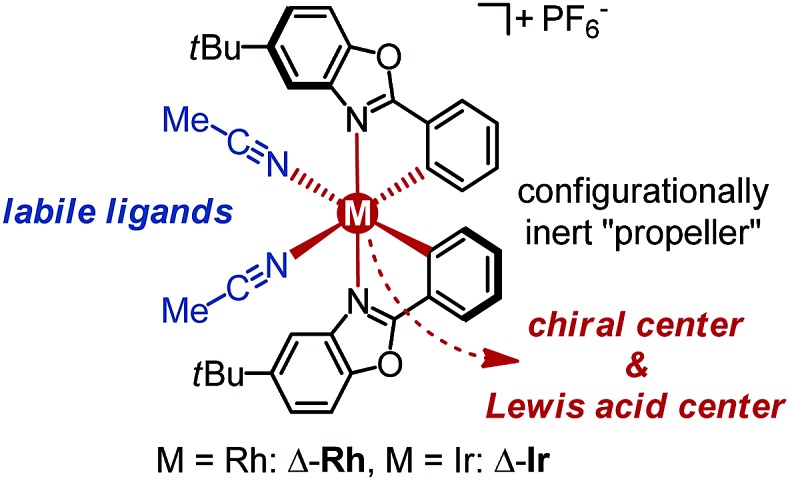
Substitutionally labile yet configurationally stable chiral-at-metal Rh^III^ (this study) and Ir^III^ (previous work) Lewis acid catalyst congeners.

We started our study by developing a synthesis of the complex Δ-**Rh**, the lighter congener of Δ-**Ir**, in which rhodium(iii) is cyclometalated in a propeller-like *C*_2_-symmetrical fashion by two *tert*-butyl-2-phenylbenzoxazoles in addition to two labile acetonitrile ligands. Despite all ligands being achiral, metal-centered chirality leads to a Δ-(right-handed propeller) and Λ-enantiomer (left-handed propeller). Accordingly, RhCl_3_ hydrate was reacted with 5-*tert*-butyl-2-phenylbenzoxazole (**1**) in 2-ethoxyethanol/water 3 : 1 under reflux to provide the rhodium dimer complex rac-**2** (62%) (Scheme 1). The subsequent reaction with d-proline afforded the rhodium(iii) prolinato complexes Δ-(*R*)-**3** and Λ-(*R*)-**3** as a mixture of diastereomers, which in our hands could not be separated by chromatography due to a limited stability of the complexes. However, fortuitously, we found that Δ-(*R*)-**3** is isolable in a straightforward fashion in a yield of 40% with high purity by just washing the mixture of diastereomers with CH_2_Cl_2_/diethyl ether. A crystal structure of Δ-(*R*)-**3** is shown in the ESI.† ^5–7^ Exposure of Δ-(*R*)-**3** to NH_4_PF_6_ in acetonitrile at 50 °C for 12 hours resulted in a substitution of d-proline with two acetonitrile ligands under complete retention of configuration to afford Δ-**Rh** in a yield of 90%. Δ-**Rh** is air stable, moisture tolerant and can be purified by standard flash silica gel chromatography. The mirror-imaged complex Λ-**Rh** is accessible in an analogous fashion by using the chiral auxiliary l-proline instead. Thus, following this convenient proline-mediated synthesis, Δ- and Λ-**Rh** can be accessed in a non-racemic fashion as verified by CD-spectroscopy (Fig. 2a).^8^ HPLC on a chiral stationary phase demonstrates that the chiral-at-rhodium complexes are virtually enantiopure (Fig. 2b). Furthermore, time dependent stability tests by ^1^H-NMR and HPLC confirm that the relative and absolute metal-centered configuration is completely retained in solution over many days (see ESI†).

**Scheme 1:**
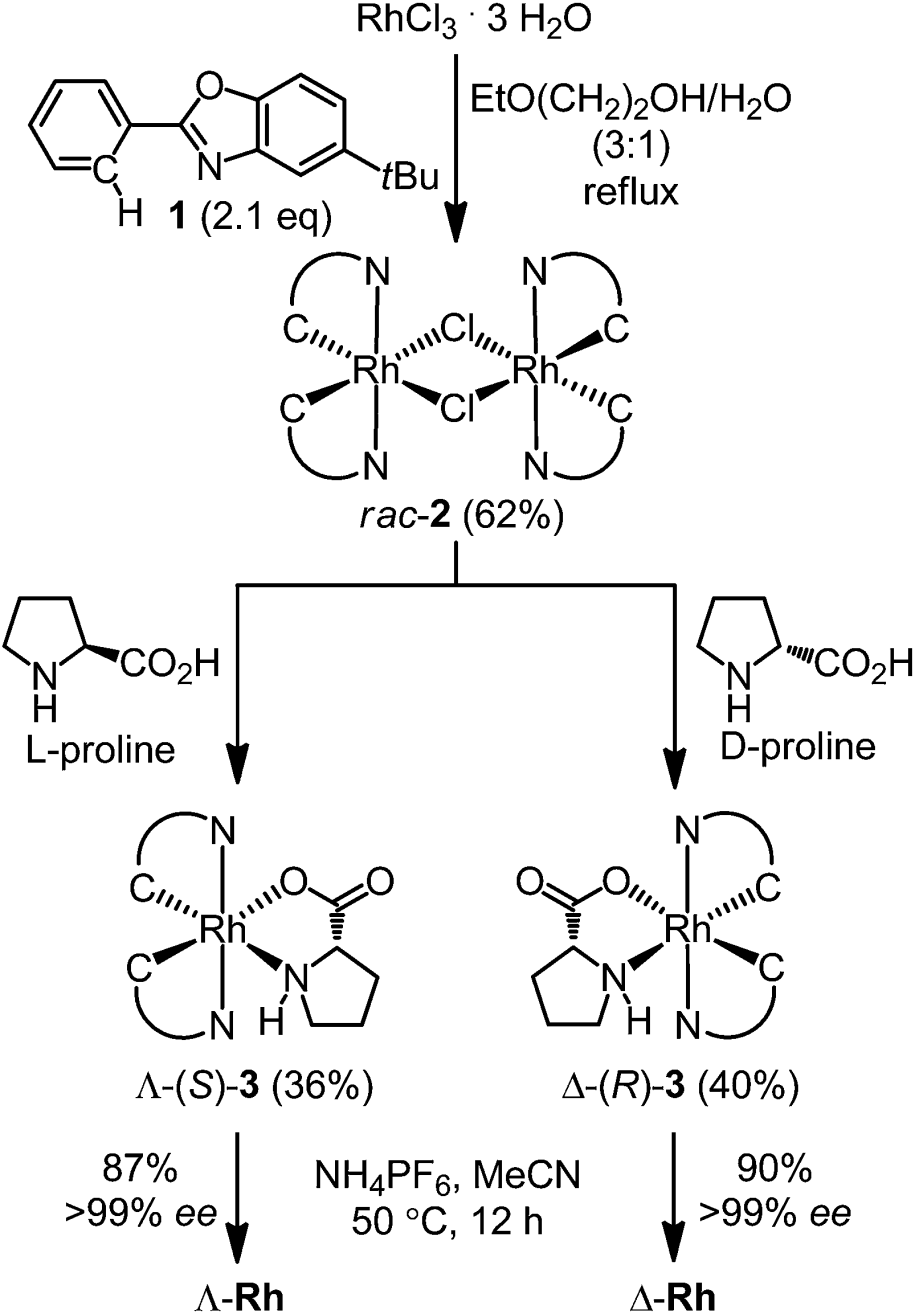
Proline-mediated synthesis of the enantiomerically pure rhodium(iii) complexes Λ-**Rh** and Δ-**Rh**.

**Fig. 2:**
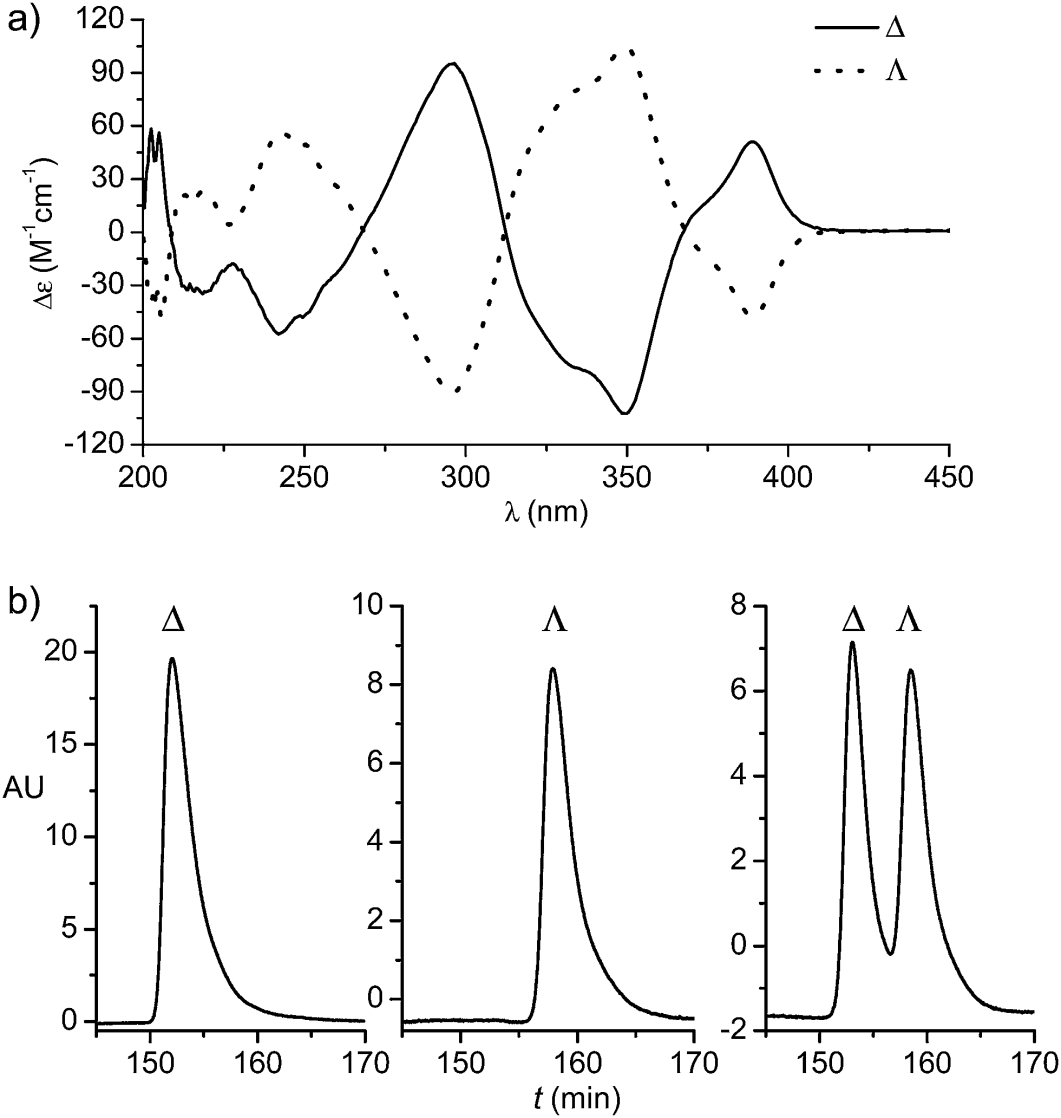
HPLC traces and CD spectra (0.2 mM in CH_3_OH) of Δ- and Λ-**Rh**.

A structure of Δ-**Rh** was obtained by single crystal X-ray diffraction and verifies the Δ-configuration at the rhodium center (Fig. 3). As expected, affected by the lanthanide contraction, the period 5 transition metal complex Δ-**Rh** and its period 6 congener Δ-**Ir** possess almost identical structures. For example, the lengths of the bonds between the transition metals and the cyclometalating benzoxazoles differ just in the range of 0.009 and 0.022 Å. However, the bonds to the coordinated acetonitrile ligands are notably longer in Δ-**Rh** compared to Δ-**Ir** by 0.041–0.043 Å, thereby indicating more exchange labile acetonitrile ligands in Δ-**Rh**.

**Fig. 3:**
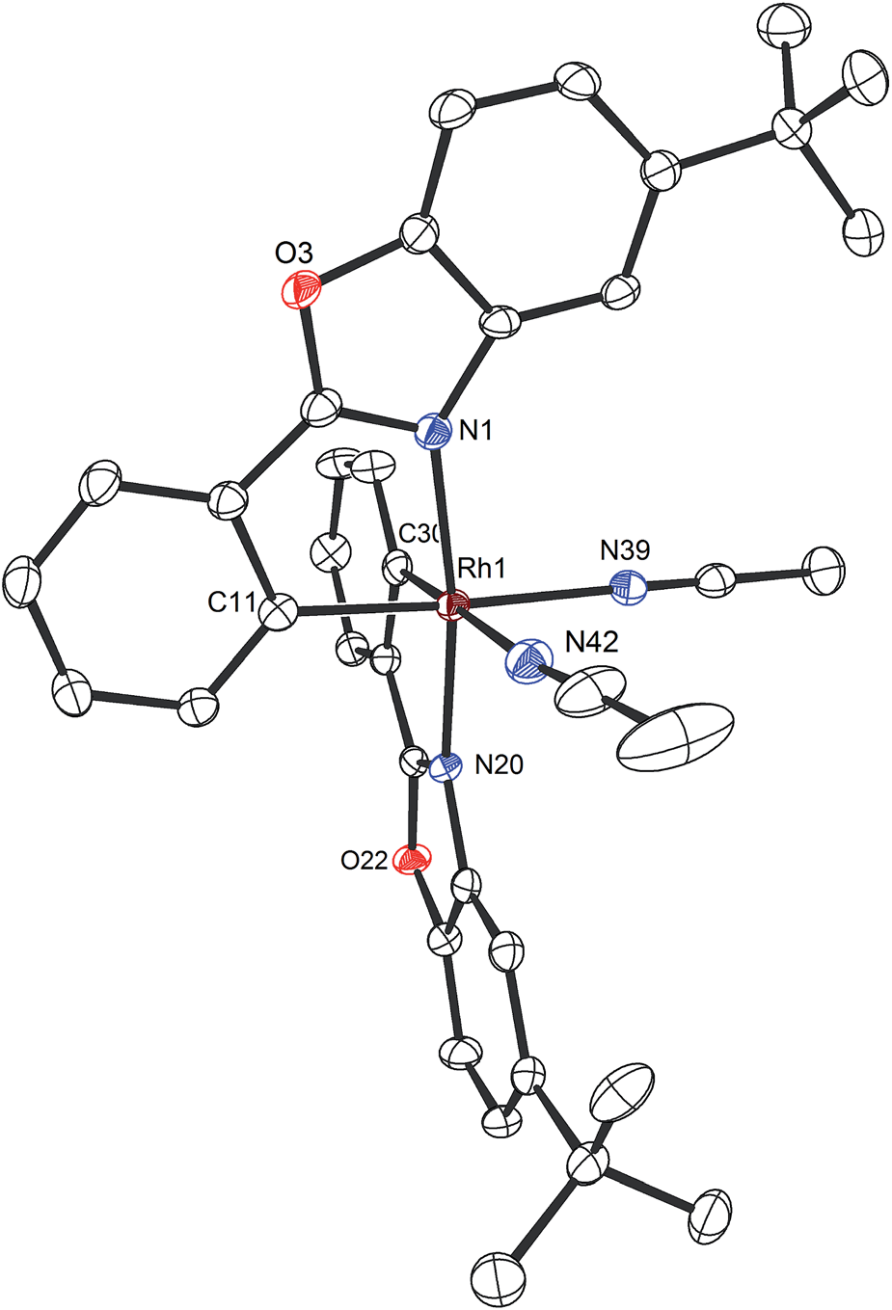
Crystal structure of the propeller-shaped catalyst Δ-**Rh**. The hexafluorophosphate counteranion is omitted for clarity. ORTEP drawing with 50% probability thermal ellipsoids.

We recently reported that Δ-**Ir** effectively catalyzes the enantioselective Friedel–Crafts addition of indoles to α,β-unsaturated 2-acyl imidazoles and we therefore used this reaction for an initial comparison of the homologous catalysts Δ-**Ir** and Δ-**Rh**.^3,9,10^ As shown in Table 1 (entry 1), although 1 mol% Δ-**Rh** catalyzes the addition of indole to enoyl imidazole **4** affording the Friedel–Crafts product (*R*)-**5a** with 94% yield and respectable 95% ee, reaction times, yields, and enantioselectivies cannot quite match the performance of the homolog Δ-**Ir**. However, to our surprise, we found that Δ-**Rh** is a superior asymmetric catalyst for the addition of CH-acidic carbonyl compounds to α,β-unsaturated 2-acyl imidazoles.^11^ For example, the addition of malodinitrile to alkene **4** catalyzed by 1 mol% Δ-**Rh** at room temperature afforded the Michael addition product (*R*)-**5b** with a significantly higher ee value of 92% compared to 89% using Δ-**Ir** (entry 2). Even more pronounced is the effect with Meldrum's acid as the nucleophile, providing (*R*)-**5d** with 85% ee using Δ-**Rh** compared to just 68% ee with Δ-**Ir** (entry 4). The enantioselectivity for the Δ-**Rh**-catalyzed reaction can be further improved significantly by either reducing the temperature to 5 °C (94% ee) or by increasing the catalyst loading to 2 mol% (95% ee). Δ-**Rh** (1 mol%) is even capable of catalyzing the formation of an all-carbon quaternary stereocenter^12^ as the reaction of *tert*-butyl 2-oxocyclopentane-1-carboxylate with acyl imidazole **4** yields (*R*,*R*)-**5e** with 99% ee and 4 : 1 dr (entry 5). Under the same conditions, Δ-**Ir** displays inferior performance with 97% ee and 3 : 1 dr and a low yield of just 41% while requiring a longer reaction time. Δ-**Rh** (1 mol%) also catalyzes the addition of 2,3-dihydro-1-oxo-1*H*-indene-2-carboxylic acid *tert*-butyl ester to acyl imidazole **4** providing the addition product (*R*,*R*)-**5f** in 92% yield with 96% ee and 14 : 1 dr. Δ-**Ir** performs similar for this transformation although the catalysis rate is somewhat sluggish and requires an elongated reaction time (72 h) for a complete conversion (entry 6). It is worth noting that the rhodium catalyst is tolerant towards moisture and air, as demonstrated for the conversion **4** → (*R*)-**5b** in which the presence of 1% H_2_O and air atmosphere did neither affect the yield nor the enantioselectivity (Table 1, entry 2).

**Table 1:** Asymmetric addition of nucleophiles to α,β-unsaturated 2-acyl imidazoles catalyzed by the congeners Δ-**Ir** and Δ-**Rh** ^*a*^

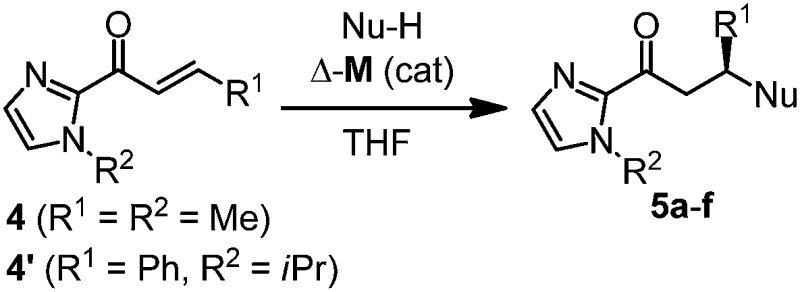						
Entry	Nucleophile	Product	Catalyst^*b*^	*T* ^*c*^ (°C)	Yield (%)	ee^*d*^ ^,^ ^*e*^ (%)
1	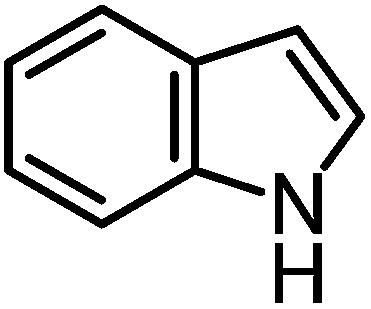	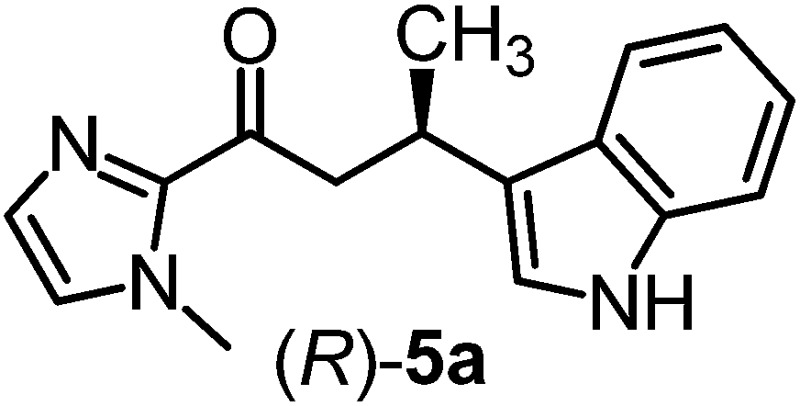	Δ-**Ir** (1.0)	Rt (20 h)	97	96
			Δ-**Rh** (1.0)	Rt (40 h)	94	95
2	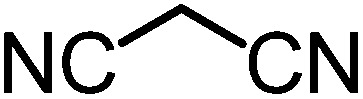	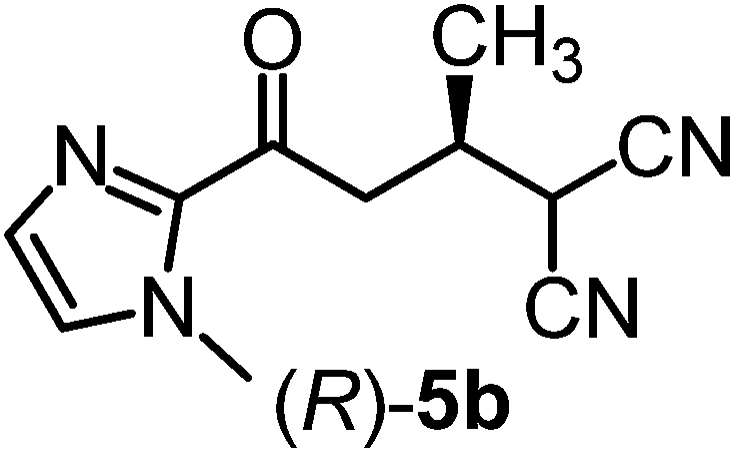	Δ-**Ir** (1.0)	Rt (16 h)	96	89
			Δ-**Rh** (1.0)	Rt (16 h)	96	92^*f*^
3	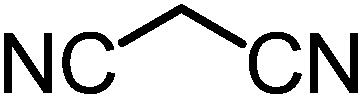	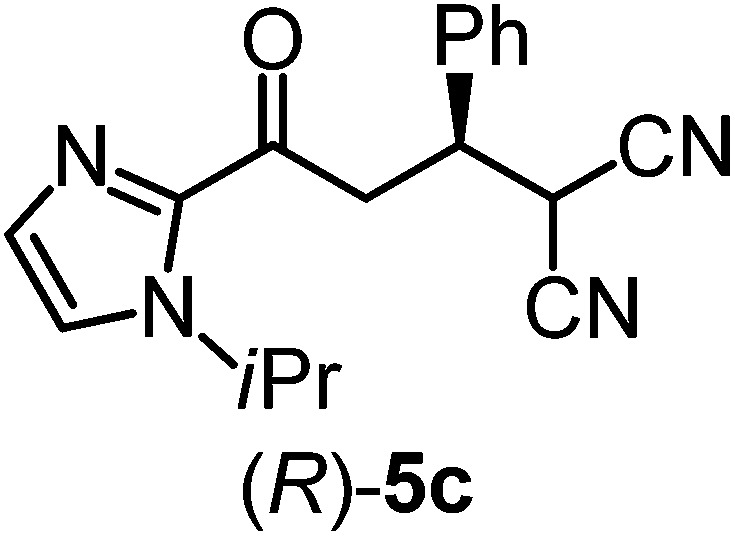	Δ-**Ir** (1.0)	Rt (96 h)	40	88
			Δ-**Rh** (1.0)	Rt (28 h)	91	95
4	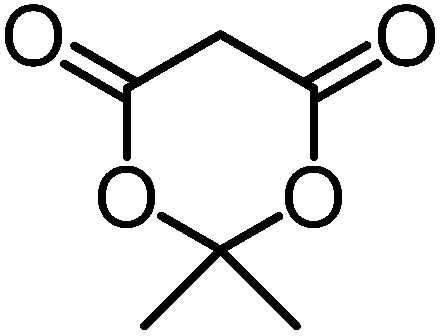	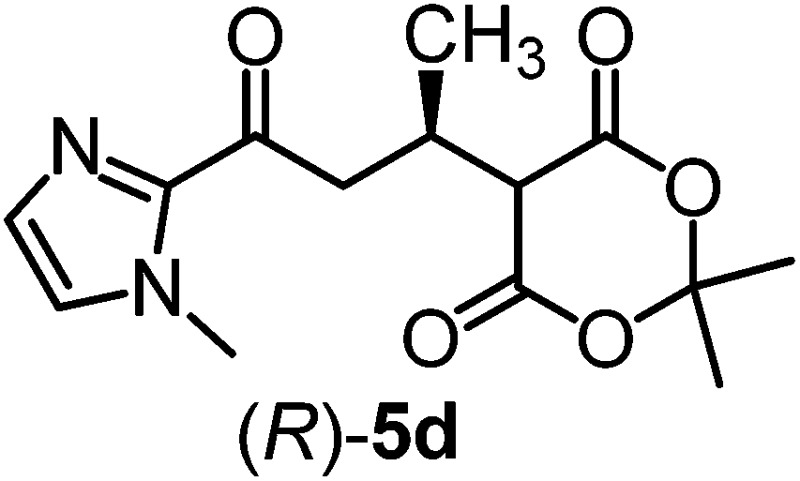	Δ-**Ir** (1.0)	Rt (16 h)	99	68
			Δ-**Rh** (1.0)	Rt (16 h)	99	85
			None	Rt (16 h)	8.5	N.d.^*g*^
			Δ-**Rh** (1.0)	5 (16 h)	97	94
			Δ-**Rh** (2.0)	Rt (6 h)	96	95
5	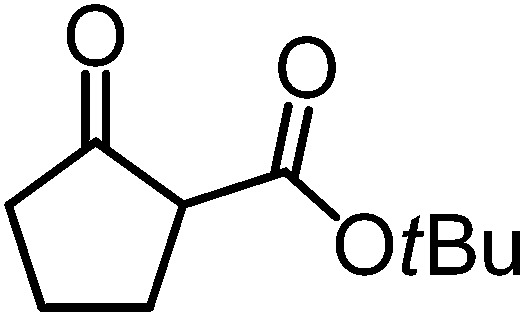	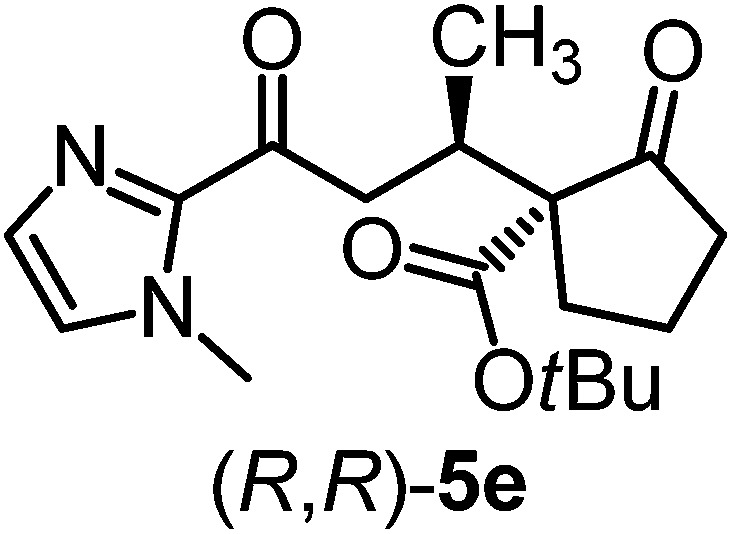	Δ-**Ir** (1.0)	Rt (96 h)	41	97 (3 : 1 dr)^*h*^
			Δ-**Rh** (1.0)	Rt (48 h)	83	99 (4 : 1 dr)
6	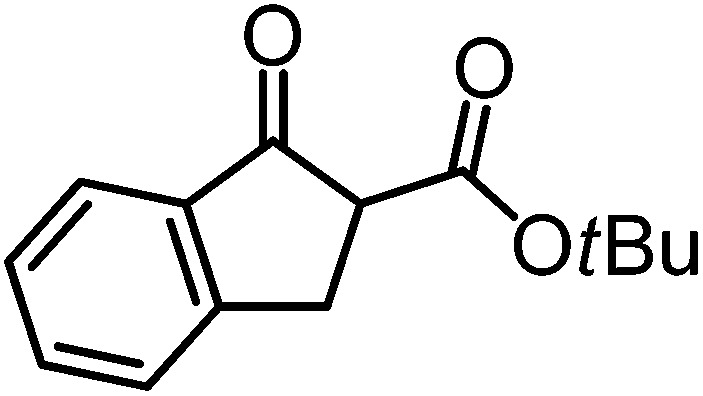	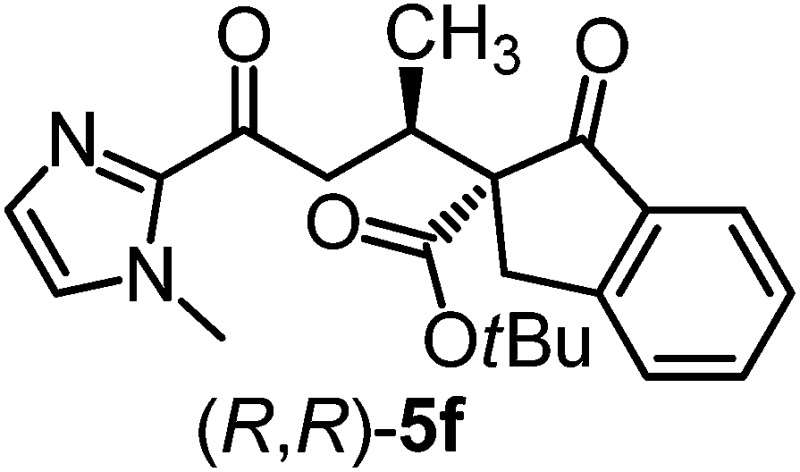	Δ-**Ir** (1.0)	Rt (72 h)	89	97 (10 : 1 dr)^*h*^
			Δ-**Rh** (1.0)	Rt (20 h)	92	96 (14 : 1 dr)

^*a*^Reaction conditions: to a Schlenk tube with the catalyst Δ-**Ir** or Δ-**Rh** (1.0 or 2.0 mol%) in distilled, anhydrous THF (entries 1 and 4: 0.20 mL, 1.0 M; entries 2, 3, 5 and 6: 0.40 mL, 0.5 M) was added acyl imidazole **4** or **4′** (0.20 mmol). After being stirred at room temperature for 20 min, the corresponding nucleophile was added at the indicated temperature and stirred at this temperature for the indicated time (monitored by TLC) under nitrogen atmosphere, and afterwards purified by flash chromatography on silica gel.

^*b*^Catalyst loadings in brackets given in mol%.

^*c*^Reaction times are given in brackets.

^*d*^Enantioselectivities were determined by HPLC chromatography on a chiral stationary phase of the purified products. Diastereoselectivities were determined by ^1^H-NMR analysis of the crude products.

^*e*^Absolute configurations were assigned in analogy to product (*R*)-**5a** (ref. 3).

^*f*^Identical yield and ee when the reaction was performed under air and in the presence of 1% H_2_O.

^*g*^Not determined.

^*h*^The relative configuration of the main diastereomers of **5e** and **5f** were assigned from a crystal structure of racemic **5f**.

Mechanistically, Δ-**Rh**, analogous to Δ-**Ir**, apparently serves as a chiral Lewis acid which coordinates in a bidentate fashion to the α,β-unsaturated 2-acyl imidazole, thereby shielding one prochiral face of the alkene and raising its electrophilicity, so that an asymmetric induction is provided in the course of the addition of the deprotonated carbon nucleophiles to the prochiral β-carbon. This mode of action is supported by a crystal structure, which was obtained upon mixing of the rhodium catalyst with an α,β-unsaturated 2-acyl imidazole substrate at room temperature, confirming the anticipated two-point coordination of the 2-acyl imidazole to the rhodium center upon replacement of the two labile acetonitrile ligands (Fig. 4). It is quite intriguing that the congeners Δ-**Rh** and Δ-**Ir** differ in their catalytic performance despite their isostructural nature, with the iridium catalyst being superior for the asymmetric Friedel–Crafts reaction, whereas the rhodium congener providing higher turnover frequencies and, in most cases, higher enantioselectivities for the shown Michael additions of β-dicarbonyl compounds. ^1^H-NMR experiments reveal that the acetonitrile exchange rates are by around an order of magnitude faster in Δ-**Rh** compared to Δ-**Ir** which is consistent with longer coordinative bonds of the metal-coordinated acetonitrile ligands in Δ-**Rh** compared to Δ-**Ir** (Fig. 3). It is therefore plausible that the superior catalytic activity of the more coordinatively labile Δ-**Rh** over the more inert Δ-**Ir** for the Michael additions with β-dicarbonyl compounds is due to substrate coordination and/or release being the rate limiting steps in the catalytic cycle, while it is the nucleophile addition step for the Friedel–Crafts reaction in which the aromaticity of the pyrrole ring is lost temporary in the course of the addition. The observed higher turnover frequencies for the Rh-catalyzed Michael additions also contribute to the observed higher enantioselectivities since a higher turnover frequency suppresses the undesired, uncatalyzed background reaction. That the uncatalyzed background reaction poses a problem and thereby affects the enantioselectivity is demonstrated for the Michael addition of Meldrum's acid (entry 4) for which we determined a significant background product formation in the absence of added catalyst (8.5% at room temperature over 16 hours). However, the additional influence of electronic effects on the stereoselectivity of asymmetric catalytic reactions is well established and may also contribute to the observed differences.^13,14^

**Fig. 4:**
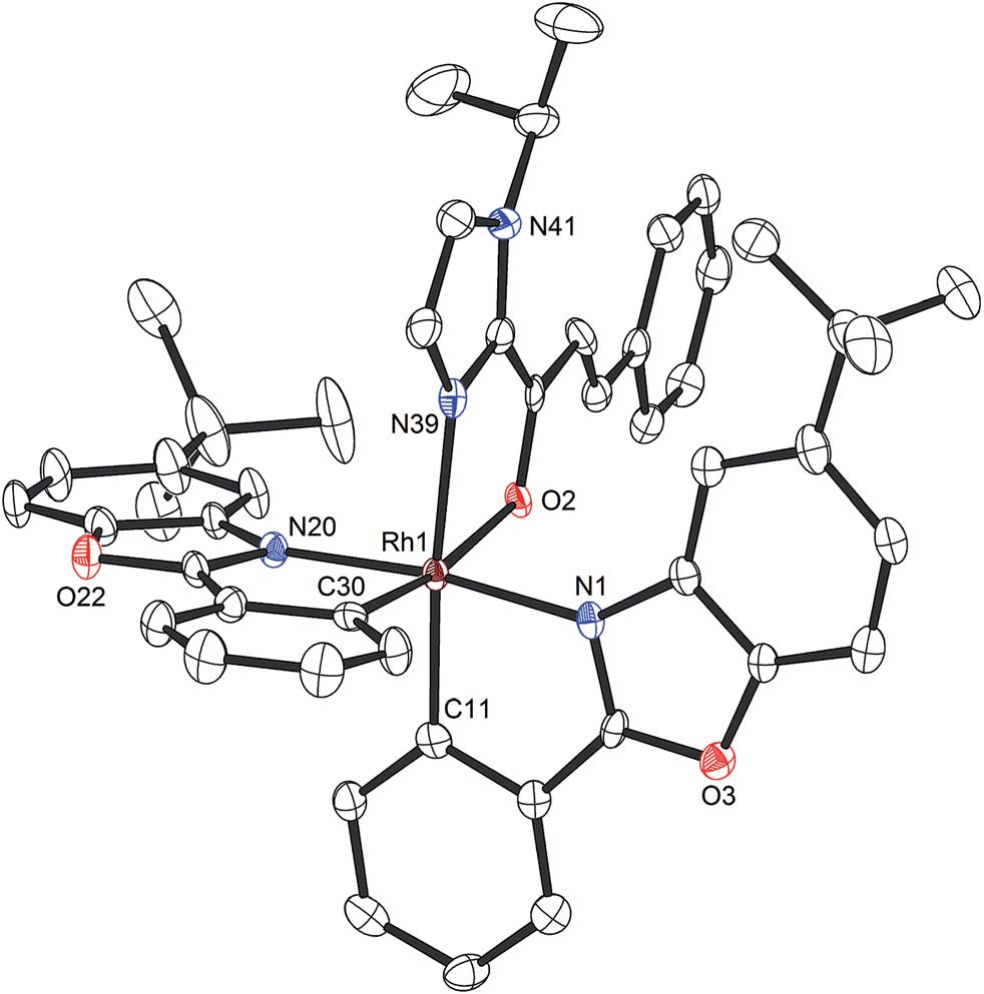
Crystal structure of α,β-unsaturated 2-acyl imidazole **4′** coordinated to the racemic rhodium catalyst upon release of the acetonitrile ligands. The hexafluorophosphate counteranion is omitted for clarity. ORTEP drawing with 50% probability thermal ellipsoids. Single crystals suitable for X-ray diffraction were obtained by reacting **4′** (0.060 mmol) with Δ/Λ-**Rh** (0.060 mmol) overnight at room temperature in CH_2_Cl_2_ (2.0 mL). Upon slow addition of *n*-hexane (5.0 mL), crystals were collected after several days (70% yield).

The usability and impact of a chiral Lewis acid scaffold correlates with its generality for asymmetric catalysis in more than one reaction family. Next, we therefore decided to investigate transformations that proceed through the activation of the α-position of carbonyl compounds and – instead of α,β-unsaturated 2-acyl imidazoles – we chose saturated 2-acyl imidazoles **6a–g** as our α-CH-acidic substrates and dibenzyl azodicarboxylate as a model electrophile.^15,16^ Interestingly, whereas the reaction of **6a** with dibenzyl azodicarboxylate in the presence of 2 mol% Λ-**Ir** afforded the α-amination product **7a** in 86% yield and 92% ee after 3 hours at room temperature, the rhodium catalyst Λ-**Rh** provided a higher yield (88%) and higher enantioselectivity (96% ee) with a 10-fold reduced catalyst loading of merely 0.2 mol% (Table 2, entry 1). Even with a catalyst loading of just 0.1 mol%, the yield (83%) and enantioselectivity (94% ee) remain satisfactory. For practical reasons, it is worth noting that the ee values can be improved to virtually complete enantiopurity by just washing the product with Et_2_O/*n*-hexane (1 : 4). The remaining substrate scope shown in Table 2 (entries 2–7) reveals that the rhodium catalyst is far superior to its iridium congener, providing higher enantioselectivities at lower catalyst loadings. Mechanistically, the rhodium(iii) and iridium(iii) catalysts apparently serve as a chiral Lewis acid by coordinating to the imidazole and carbonyl group of 2-acyl imidazoles in a bidentate fashion, thereby triggering its deprotonation to an intermediate enolate complex, which subsequently reacts with the protonated azodicarboxylate to form the coordinated α-amination product. To strengthen our mechanistic proposal, a crystal structure of the proposed intermediate metal enolate complex was obtained by reacting the iridium catalyst with 2-acyl imidazole **6a** under slightly basic conditions. The structure shown in Fig. 5 also visualizes that the *Si*-face of the enolate α-carbon is shielded by one *tert*-butyl group and thus provides an effective asymmetric induction. The much higher catalytic activity of the rhodium catalyst over its iridium congener can be attributed to the significantly higher lability of the coordinative bonds to rhodium which allows a much faster turnover. This is further supported by determined initial rates for the α-amination **6a** → **7a** which are by a factor of 21 higher for Λ-**Rh** compared to Λ-**Ir**.

**Table 2:** Asymmetric α-amination of 2-acyl imidazoles catalyzed by the congeners Λ-**Ir** and Λ-**Rh** ^*a*^

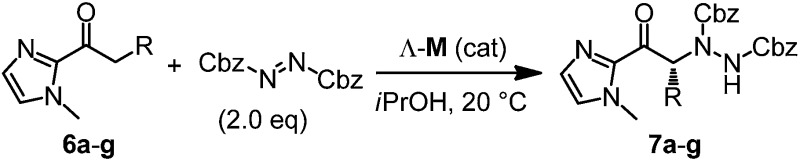						
Entry	Starting Cpds	Product	Catalyst (mol%)	Time (h)	Yield^*b*^ (%)	ee^*c*^ (%)
1	R = Ph (**6a**)	(*R*)-**7a**	Λ-**Ir** (2.0)	3	86	92 (>99.5)
			Λ-**Rh** (0.2)	4	88	96 (>99.5)
			Λ-**Rh** (0.1)	15	83	94 (>99.5)
			None	11	4	N.d.^*d*^
2	R = 2-MePh (**6b**)	(*R*)-**7b**	Λ-**Ir** (2.0)	5	81	91 (>99.5)
			Λ-**Rh** (0.2)	4	84	94 (>99.5)
3	R = 4-MeOPh (**6c**)	(*R*)-**7c**	Λ-**Ir** (2.0)	4	87	95 (99)
			Λ-**Rh** (0.2)	6	85	97 (99)
4	R = 4-ClPh (**6d**)	(*R*)-**7d**	Λ-**Ir** (2.0)	5	82	79 (84)
			Λ-**Rh** (0.5)	8	83	95 (97)
5	R = 2-Naph (**6e**)	(*R*)-**7e**	Λ-**Ir** (2.0)	4	83	90 (>99.5)
			Λ-**Rh** (0.2)	6	86	96 (99)
6	R = 3-thienyl (**6f**)	(*R*)-**7f**	Λ-**Ir** (2.0)	8	71	80 (94)
			Λ-**Rh** (0.2)	12	64	90 (97)
7	R = Me (**6g**)	(*R*)-**7g**	Λ-**Ir** (2.0)	16	85	91
			Λ-**Rh** (1.0)	22	95	92

^*a*^Reaction conditions: to **6a–g** (0.20 mmol) in anhydrous *i*PrOH (0.10 mL, 2.0 M) was added the catalyst, stirred at room temperature for 30 min, before dibenzyl azodicarboxylate (0.40 mmol) was added and the reaction was stirred for the indicated time at 20 °C.

^*b*^Isolated yields.

^*c*^Enantiomeric excess determined by HPLC on chiral stationary phase. Enantiomeric purities after washing with Et_2_O/*n*-hexane (1 : 4) are provided in brackets.

^*d*^Not determined.

**Fig. 5:**
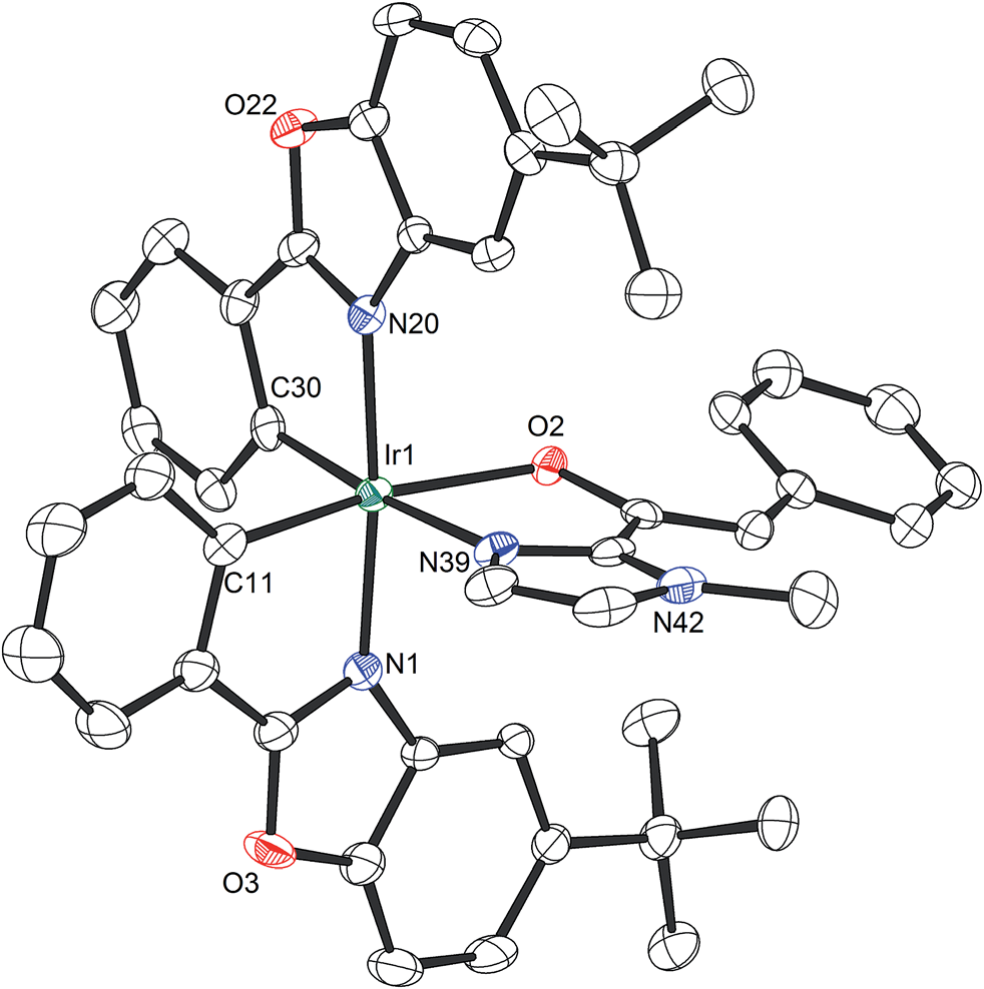
Crystal structure of an iridium enolate complex as a proposed catalytic intermediate in the α-amination of 2-acyl imidazole **6a** catalyzed by the iridium(iii) catalyst. Only one enantiomer is shown and solvent molecules are omitted for clarity.

In conclusion, we here reported the first example of an asymmetric catalyst which derives both its optical activity and Lewis acidity from an octahedral rhodium stereocenter. This novel, configurationally surprisingly stable chiral Lewis acid is conceptually very simple, as it just contains achiral mono- and bidentate ligands, and it can be accessed conveniently in an enantiomerically pure fashion through a proline-mediated synthesis. Interestingly, although isostructural to its iridium congener, the two homologs differ significantly in their catalytic Lewis acid activity, with the rhodium complex demonstrating advantages as catalyst for the Michael addition of CH-acidic β-dicarbonyl compounds to α,β-unsaturated 2-acyl imidazoles and for the α-functionalization of saturated 2-acyl imidazoles. The superiority of the rhodium catalyst over its iridium congener can in large parts be attributed to a significantly higher lability of the two accessible rhodium coordination sites which allow higher turnover frequencies and turnover numbers. We believe that the here introduced class of chiral-at-rhodium(iii) complexes will be of widespread use as chiral Lewis acid catalysts for a large variety of asymmetric transformations. Investigations along these lines are undergoing in our laboratory.
