# Supplementary material for: Asymmetric Lewis acid catalysis directed by octahedral rhodium centrochirality
Source: Chem Sci. 2014 Nov 10;6(2):1094–100. doi: 10.1039/c4sc03101f (PMC5811158; doi:10.1039/c4sc03101f)
Supplement: Supplementary file 1 [file SC-006-C4SC03101F-s001.pdf]

Electronic Supplementary Material (ESI) for Chemical Science.
This journal is © The Royal Society of Chemistry 2014

Supporting Information

Asymmetric Lewis Acid Catalysis Directed by Octahedral Rhodium Centrochirality

Chuanyong Wang, Liang-An Chen, Haohua Huo, Xiaodong Shen, Klaus Harms, Lei Gong and Eric Meggers

Contents

1. General Information S2

2. Synthesis of Rhodium Catalysts Λ-**Rh** and Δ-**Rh** S3

3. Catalysis Reactions S7

4. Assignment of Absolute Configurations S18

5. HPLC on Chiral Stationary Phase S20

6. Investigation of the Proposed Catalyst-Coordinated Substrate S42

7. Investigation of the Configurational Stability of the Rhodium Catalyst S44

8. NMR Spectra of New Rhodium Complexes S47

9. Single Crystal X-Ray Diffraction S50

10. References S56

1. General Information

All reactions were carried out under an atmosphere of nitrogen or argon with magnetic stirring. Catalysis reactions were performed using standard Schlenk glassware techniques (Michael reactions) or in brown glass vials (enolate chemistry). Solvents were distilled under nitrogen from calcium hydride (CH_3CN, CH_2Cl_2), sodium/benzophenone (THF), or magnesium turnings/iodine (MeOH). ^1H NMR and proton decoupled ^{13}C NMR spectra were recorded on Bruker Avance 300 (300 MHz), Bruker AM (400 MHz) or Bruker AM (500 MHz) spectrometers at ambient temperature. NMR standards were used as follows: ^1H NMR spectroscopy: δ = 7.26 ppm ($CDCl_3$), δ = 5.32 ppm (CD_2Cl_2). $^{13}C\{^1H\}$ NMR spectroscopy: δ = 77.1 ppm ($CDCl_3$), δ = 53.8 ppm (CD_2Cl_2). IR spectra were recorded on a Bruker Alpha FT-IR spectrophotometer or on a Nicolet Avatar 330 FT-IR spectrophotometer. CD spectra were recorded on a JASCO J-810 CD spectropolarimeter (600-200 nm, 1 nm bandwidth, 50 nm/min scanning speed, accumulation of 3 scans). High-resolution mass spectra were recorded on a Bruker En Apex Ultra 7.0 TFT-MS instrument using ESI technique. Chiral HPLC chromatography was performed with an Agilent 1200 HPLC system. Optical rotations were measured with a Perkin-Elmer 241 or 341 polarimeter at concentrations of 1.0 g/100 mL. Substrates **4** and **4'**,[1] *tert*-butyl β-ketoesters,[2] and complexes Λ- and Δ-**Ir**[3] were synthesized according to reported procedures. All other reagents were commercially available and used without further purification.

2. Synthesis of Rhodium Catalysts Λ-Rh and Δ-Rh

5-*tert*-Butyl-2-phenylbenzo[*d*]oxazole (1)

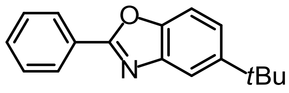

The compound was synthesized following a published procedure with slight modifications.[4] A solution of 2-amino-4-*tert*-butylphenol (0.825 g, 5.0 mmol) and benzaldehyde (0.50 mL, 5.0 mmol) in *m*-xylene (16.0 mL) was stirred at 120 °C for 30 min. 4-Methoxy-TEMPO (46.5 mg, 5 mol%) was added to the mixture and the reaction was stirred at this temperature for further 8 h under an oxygen atmosphere. The reaction mixture was cooled to room temperature and concentrated under reduced pressure. The residue was purified by flash chromatography on silica gel (EtOAc/*n*-hexane = 1:20) to obtain the product **1** (1.152 g, 4.6 mmol, yield: 92%) as a white solid.

^{1}H NMR (300 MHz, $CDCl_3$) δ 8.29-8.22 (m, 2H), 7.81 (d, J = 1.8 Hz, 1H), 7.56-7.48 (m, 4H), 7.42 (dd, J = 8.6, 1.9 Hz, 1H), 1.40 (s, 9H).

All spectroscopic data are in agreement with the literature.[5]

Precursor Rhodium Complex *rac*-2

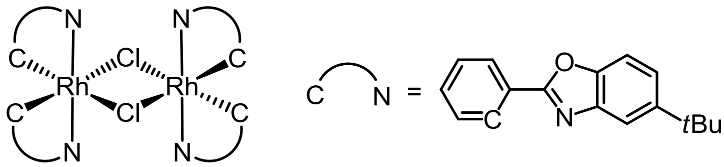

Compound *rac*-**2** was synthesized according to a route reported by Mesmaeker for rhodium(III) μ-chloro-bridged dimers with related cyclometalated ligands.[6] Accordingly, 5-*tert*-butyl-2-phenylbenzo[*d*]oxazole **1** (1.030 g, 4.1 mmol) was added to $RhCl_3$•$3H_2O$ (418.5 mg, 2.0 mmol) in a mixture of 2-ethoxyethanol and water (3:1, 92.0 mL). The reaction mixture was heated at 120 °C for 24 h under an atmosphere of nitrogen. The resulting precipitate was collected by centrifugation, washed with methanol and dried to obtain the product *rac*-**2** (792.4 mg, 0.62 mmol, yield: 62%) as a pale yellow solid.

^{1}H NMR (300 MHz, CD_2Cl_2) δ 8.38 (t, J = 1.2 Hz, 4H), 7.58 (dd, J = 7.6, 1.3 Hz, 4H), 7.31-7.20 (m, 8H), 6.97 (td, J = 7.4, 0.9 Hz, 4H), 6.77 (td, J = 7.6, 1.5 Hz, 4H), 6.12 (d, J = 7.9 Hz, 4H), 1.22 (s, 36H).

^{13}C NMR (75 MHz, CD_2Cl_2) δ 170.4 (4C), 164.8 (4C), 164.4 (4C), 149.0 (4C), 148.0 (4C), 139.6 (4C), 133.6 (4C), 131.4 (4C), 125.5 (4C), 124.1 (4C), 123.1 (4C), 115.8 (4C), 110.5 (4C), 35.4 (4C), 31.8 (12C).

IR (film): ν (cm^{-1}) 3055, 2957, 2870, 1589, 1526, 1441, 1373, 1271, 1196, 1120, 1075, 1027, 929, 892, 808, 727, 702, 647, 448.

HRMS (ESI, *m/z*) calcd for $C_{68}H_{64}Rh_2N_4O_4Cl$ [M–Cl]$^+$: 1241.2721, found: 1241.2709.

Rhodium Auxiliary Complexes Λ-(*S*)-3 and Δ-(*R*)-3

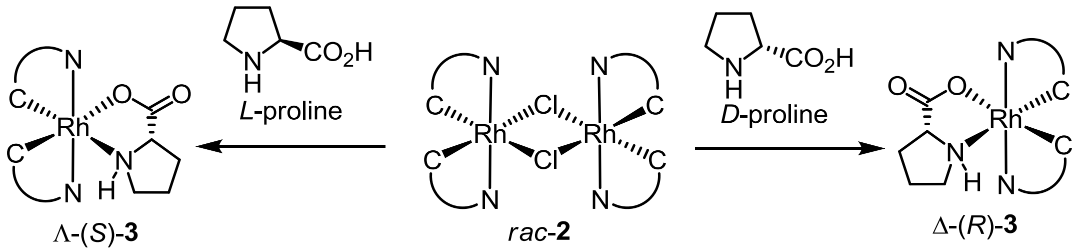

The rhodium auxiliary complexes Λ-(*S*)-**3** and Δ-(*R*)-**3** were synthesized according to a reported method[7] with some modifications. To a solution of NaOMe (16.2 mg, 0.30 mmol) in methanol (16.0 mL), L-proline (34.5 mg, 0.30 mmol) or D-proline (34.5 mg, 0.30 mmol) was added in one portion. The mixture was stirred for 10 min, to which a suspension of rhodium dimer (201.3 mg, 0.15 mmol) was added. The mixture was stirred and heated at 50 °C for 12 h. After the mixture cooled to room temperature, CH_2Cl_2 (16.0 mL) was added. The reaction mixture was stirred for a further 12 h to give a clear, yellow solution. The solvent was removed in vacuo and the mixture of two diastereoisomers was washed with dichloromethane / diethyl ether (1:6, v/v) until the filtrate was almost colorless. The residual insoluble solid was dried and collected as Λ-(*S*)-**3** (77.4 mg, 36%) or Δ-(*R*)-**3** (86.1 mg, 40%). The absolute configurations of the obtained Λ-(*S*)/Δ-(*R*) configured rhodium(III) complexes were assigned by an X-ray crystal structure of Δ-(*R*)-**3**. CD spectroscopy confirmed that the complexes are enantiomers.

^{1}H NMR (300 MHz, CD_2Cl_2) δ 8.17 (d, *J* = 1.8 Hz, 1H), 7.84-7.79 (m, 2H), 7.73 (dd, *J* = 15.2, 8.8 Hz, 2H), 7.63 (td, *J* = 9.0, 1.8 Hz, 2H), 7.36 (d, *J* = 1.6 Hz, 1H), 7.12-7.02 (m, 2H), 6.95 (td, *J* = 7.5, 1.5 Hz, 2H), 6.76 (d, *J* = 7.7 Hz, 1H), 6.49 (d, *J* = 7.7 Hz, 1H), 4.34-4.17 (m, 2H), 2.80-2.67 (m, 1H), 2.30-2.13 (m, 2H), 2.07-1.94 (m, 1H), 1.68-1.53 (m, 2H), 1.45 (d, *J* = 7.6 Hz, 18H).

^{13}C NMR (75 MHz, CD_2Cl_2) δ 180.5, 172.7, 172.6, 171.5, 171.4, 167.4, 167.0, 166.1, 165.7, 151.4, 150.6, 149.09, 149.06, 138.9, 138.3, 135.4, 134.4, 131.7, 131.3, 131.2, 131.0, 126.3, 126.0, 124.3, 124.2, 123.5, 123.1, 115.7, 112.2, 111.5, 111.1, 64.3, 49.7, 35.8, 35.6, 32.0, 31.9, 30.4, 27.3.

IR (film): ν (cm^{-1}) 3146, 3056, 2958, 1591, 1524, 1445, 1373, 1270, 1191, 1122, 1077, 1033, 928, 814, 773, 648, 550, 449.

Λ-(*S*)-**3**:

HRMS (ESI, *m*/*z*) calcd for $C_{39}H_{41}RhN_3O_4$ $[M+H]^+$: 718.2147, found: 718.2133.

CD (MeOH): λ, nm (Δε, $M^{-1}cm^{-1}$) 394 (–28), 353 (+41), 295 (–34), 253 (+23), 233 (–6), 216 (+53), 203 (–80).

Δ-(*R*)-**3**:

HRMS (ESI, *m*/*z*) calcd for $C_{39}H_{40}RhN_3O_4Na$ $[M+Na]^+$: 740.1966, found: 740.1930.

CD (MeOH): λ, nm (Δε, $M^{-1}cm^{-1}$) 394 (+15), 353 (–18), 295 (+22), 253 (–9), 233 (+6), 216 (–24), 203 (+45).

Synthesis of Non-Racemic Rhodium Catalysts

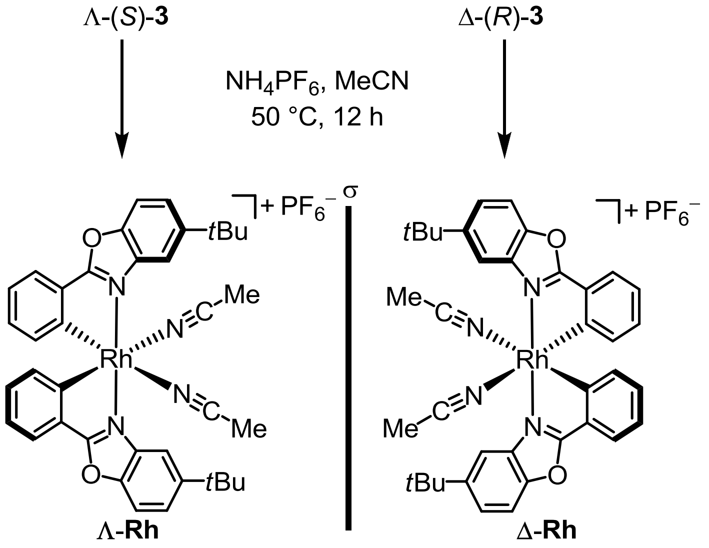

A suspension of the rhodium auxiliary complex Λ-(*S*)-**3** (71.7 mg, 0.10 mmol) or Δ-(*R*)-**3** (71.7 mg, 0.10 mmol) and NH_4PF_6 (163.0 mg, 1.00 mmol) in acetonitrile (20.0 mL) was heated at 50 °C for 12 h under nitrogen in the dark. The reaction mixture was concentrated to dryness and subjected to flash silica gel chromatography (100% CH_2Cl_2 to CH_2Cl_2/CH_3CN = 15:1) to give the enantiopure catalyst Λ-**Rh** (72.2 mg, 0.087 mmol, 87%) or Δ-**Rh** (74.7 mg, 0.090 mmol, 90%) as a pale yellow solid. The absolute configurations of the obtained Λ- and Δ-configured rhodium(III) complexes

were verified by CD spectroscopy and confirmed by an X-ray crystal structure of Δ-**Rh**. The enantiomeric purity was verified by HPLC analysis with a chiral stationary phase.

^{1}H NMR (300 MHz, CD_2Cl_2) δ 7.88 (d, J = 1.6 Hz, 2H), 7.80-7.74 (m, 6H), 7.09 (td, J = 7.5, 0.9 Hz, 2H), 6.94 (td, J = 7.6, 1.5 Hz, 2H), 6.40 (d, J = 7.8 Hz, 2H), 2.31 (s, 6H), 1.46 (s, 18H).

^{13}C NMR (75 MHz, CD_2Cl_2) δ 171.5, 160.2, 159.8, 151.2, 148.9, 138.2, 133.5, 132.4, 130.6, 126.3, 125.3, 124.6, 122.0, 113.4, 112.0, 35.7, 31.9, 3.7.

IR (film): ν (cm^{-1}) 2957, 1593, 1528, 1446, 1381, 1274, 1193, 1126, 1081, 1033, 931, 835, 730, 649, 555, 449.

Λ-**Rh**:

HRMS (ESI, *m*/*z*) calcd for $C_{38}H_{38}RhN_4O_2$ $[M-PF_6]^+$: 685.2044, found: 685.2036.

CD (MeOH): λ, nm (Δε, M^{-1}cm^{-1}) 390 (–48), 350 (+104), 295 (–92), 242 (+56), 228 (+5), 218 (+24), 204 (–46).

Δ-**Rh**:

HRMS (ESI, *m*/*z*) calcd for $C_{38}H_{38}RhN_4O_2$ $[M-PF_6]^+$: 685.2044, found: 685.2026.

CD (MeOH): λ, nm (Δε, M^{-1}cm^{-1}) 390 (+51), 350 (–104), 295 (+94), 242 (–58), 228 (–20), 218 (–34), 204 (+57).

3. Catalysis Reactions

3.1. Asymmetric Michael Reactions

General Procedure (Reactions of Table 1). To a solution of catalyst Δ-**Ir** or Δ-**Rh** (1 mol%) in distilled, anhydrous THF (entries 1 and 4: 0.20 mL, 1.0 M; entries 2, 3, 5 and 6: 0.40 mL, 0.5 M) was added the 2-acyl imidazole **4** or **4'** (0.20 mmol) in a Schlenk tube. After being stirred at room temperature for 20 min, the corresponding nucleophile was added at room temperature or 5 °C. The reaction was stirred at the indicated temperature for the indicated time (monitored by TLC) under nitrogen atmosphere. Afterwards, the mixture was concentrated under reduced pressure. The residue was purified by flash chromatography on silica gel (EtOAc/*n*-hexane = 1:2 to 2:1) to afford the products **5a-f**. The dr values were determined by ^{1}H NMR analysis of the crude products, and the ee values were determined by chiral HPLC chromatography using a Chiralpak IC or AD-H column.

(*R*)-3-(1*H*-Indol-3-yl)-1-(1-methyl-1*H*-imidazol-2-yl)butan-1-one (5a)

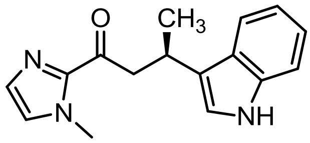

Starting from **4** (30.2 mg, 0.20 mmol) and 1*H*-indole (58.6 mg, 0.50 mmol) according to the general procedure to give **5a** as a white solid (catalyzed by Δ-**Ir**: 51.9 mg, yield: 97%, ee: 96%; catalyzed by Δ-**Rh**: 50.2 mg, yield: 94%, ee: 95%). Enantiomeric excess established by HPLC analysis using a Chiralpak IC column (HPLC: IC, 254 nm, *n*-hexane/isopropanol = 85:15, flow rate 0.5 mL/min, 40 °C, t_r (major) = 22.4 min, t_r (minor) = 25.9 min); $[\alpha]_D^{20}$ = +13.8° (*c* 0.5, CH_2Cl_2) for 95% ee of **5a** (Lit.[3] $[\alpha]_D^{20}$ = –14.5° (*c* 2.7, CH_2Cl_2) for 96% ee of product with *S*-configuration).

^{1}H NMR (300 MHz, $CDCl_3$) δ 8.41 (br s, 1H), 7.67 (d, *J* = 7.8 Hz, 1H), 7.34-7.28 (m, 1H), 7.22-7.05 (m, 3H), 7.01-6.95 (m, 2H), 3.93 (s, 3H), 3.91-3.78 (m, 1H), 3.66-3.34 (m, 2H), 1.40 (d, *J* = 6.9 Hz, 3H).

^{13}C NMR (75 MHz, $CDCl_3$) δ 192.4, 143.4, 136.5, 128.9, 127.0, 126.7, 121.9, 121.5, 120.3, 119.3, 119.1, 111.2, 46.8, 36.2, 27.2, 21.8.

All spectroscopic data were in agreement with the literature.[1]

(*R*)-2-(4-(1-Methyl-1*H*-imidazol-2-yl)-4-oxobutan-2-yl)malononitrile (5b)

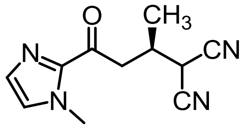

Starting from **4** (30.2 mg, 0.20 mmol) and malononitrile (15.8 mg, 0.24 mmol) according to the general procedure to give **5b** as a colorless oil (catalyzed by Δ-**Ir**: 41.5 mg, yield: 96%, ee: 89%; catalyzed by Δ-**Rh**: 41.5 mg, yield: 96%, ee: 92%). Enantiomeric excess established by HPLC analysis using a Chiralpak AD-H column (HPLC: AD-H, 254 nm, *n*-hexane/isopropanol = 90:10, flow rate 1.0 mL/min, 25 °C, t_r (major) = 22.8 min, t_r (minor) = 24.1 min); $[\alpha]_D^{20}$ = –33.2° (*c* 0.4, CH_2Cl_2) for 92% ee of **5b**.

^{1}H NMR (300 MHz, $CDCl_3$) δ 7.15 (s, 1H), 7.08 (s, 1H), 4.37 (d, *J* = 4.9 Hz, 1H), 3.99 (s, 3H), 3.47 (dd, *J* = 18.0, 5.3 Hz, 1H), 3.28 (dd, *J* = 18.0, 8.2 Hz, 1H), 2.89-2.71 (m, 1H), 1.36 (d, *J*= 6.9 Hz, 3H).

^{13}C NMR (75 MHz, $CDCl_3$) δ 189.6, 142.4, 129.8, 127.9, 112.5, 111.5, 41.6, 36.2, 31.9, 28.2, 17.3.

All spectroscopic data were in agreement with the literature.[8]

(*R*)-2-(3-(1-Isopropyl-1*H*-imidazol-2-yl)-3-oxo-1-phenylpropyl)malononitrile (5c)

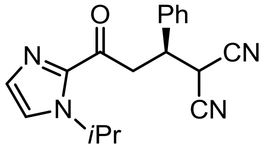

Starting from **4'** (45.8 mg, 0.20 mmol) and malononitrile (15.8 mg, 0.24 mmol) according to the general procedure to give **5c** as a colorless oil (catalyzed by Δ-**Ir**: 24.7 mg, yield: 40%, ee: 88%; catalyzed by Δ-**Rh**: 55.8 mg, yield: 91%, ee: 95%). Enantiomeric excess established by HPLC analysis using a Chiralpak AD-H column (HPLC: AD-H, 254 nm, *n*-hexane/isopropanol = 70:30, flow rate 0.8 mL/min, 25 °C, t_r (minor) = 13.0 min, t_r (major) = 23.6 min); $[\alpha]_D^{20}$ = –0.4° (*c* 0.8, CH_2Cl_2) for 95% ee of **5c**.

^{1}H NMR (300 MHz, $CDCl_3$) δ 7.46-7.35 (m, 5H), 7.30 (d, *J* = 0.9 Hz, 1H), 7.19 (d, *J* = 0.8 Hz, 1H), 5.42 (dt, *J* = 13.4, 6.7 Hz, 1H), 4.53-4.45 (m, 1H), 4.03-3.88 (m, 2H), 3.87-3.72 (m, 1H), 1.41 (dd, *J* = 8.6, 6.7 Hz, 6H).

^{13}C NMR (75 MHz, $CDCl_3$) δ 188.7, 141.5, 136.5, 130.2, 129.2, 129.0, 128.2, 122.1, 112.0, 111.7, 49.5, 41.7, 41.2, 29.3, 23.55, 23.52.

IR (film): *ν* (cm^{-1}) 3034, 2983, 2909, 2254, 1670, 1497, 1465, 1454, 1395, 1371, 1254, 1199, 1162, 1087, 971, 914, 772, 731, 700, 671, 646, 591, 548, 488, 407.

HRMS (ESI, *m/z*) calcd for $C_{18}H_{18}N_4ONa$ $[M+Na]^+$: 329.1373, found: 329.1369.

(*R*)-2,2-Dimethyl-5-(4-(1-methyl-1*H*-imidazol-2-yl)-4-oxobutan-2-yl)-1,3-dioxane-4,6-dione (5d)

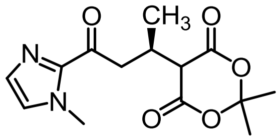

Starting from **4** (30.2 mg, 0.20 mmol) and 2,2-dimethyl-1,3-dioxane-4,6-dione (86.5 mg, 0.6 mmol) according to the general procedure to give **5d** as a white solid (catalyzed by Δ-**Ir**: 58.3 mg, yield: 99%, ee: 68%; catalyzed by Δ-**Rh**: 58.3 mg, yield: 99%, ee: 85%). Enantiomeric excess established by HPLC analysis using a Chiralpak AD-H column (HPLC: AD-H, 254 nm, *n*-hexane/isopropanol = 90:10, flow rate 0.8 mL/min, 40 °C, t_r (major) = 24.9 min, t_r (minor) = 26.6 min); $[\alpha]_D^{20}$ = –3.3° (*c* 0.8, CH_2Cl_2) for 95% ee of **5d** (catalyzed by Δ-**Rh** (2 mol%) at room temperature).

^{1}H NMR (300 MHz, $CDCl_3$) *δ* 7.13 (d, *J* = 0.9 Hz, 1H), 7.03 (s, 1H), 4.22-4.16 (m, 1H), 3.98 (s, 3H), 3.56 (dd, *J* = 7.2, 5.0 Hz, 2H), 3.24-3.10 (m, 1H), 1.77 (d, *J* = 5.8 Hz, 6H), 1.21 (d, *J* = 7.0 Hz, 3H).

^{13}C NMR (75 MHz, $CDCl_3$) *δ* 192.3, 165.2, 164.7, 142.9, 129.2, 127.2, 104.7, 49.3, 41.9, 36.2, 28.9, 28.4, 26.9, 17.3.

IR (film): *ν* (cm^{-1}) 3136, 2928, 2883, 1780, 1739, 1667, 1459, 1408, 1298, 1200, 1151, 1086, 1053, 991, 958, 914, 871, 787, 698, 670, 634, 596, 544, 496, 426.

HRMS (ESI, *m/z*) calcd for $C_{14}H_{19}N_2O_5$ $[M+H]^+$: 295.1288, found: 295.1282.

(*R*)-*tert*-Butyl 1-((*R*)-4-(1-methyl-1*H*-imidazol-2-yl)-4-oxobutan-2-yl)-2-oxocyclopentanecarb-oxylate (5e)

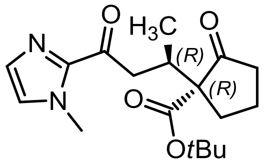

Starting from **4** (30.2 mg, 0.20 mmol) and *tert*-butyl 2-oxocyclopentanecarboxylate (73.7 mg, 0.40 mmol) according to the general procedure to give **5e** (major product) as a colorless oil (catalyzed by

Δ-**Ir**: 27.4 mg, yield: 41%, ee: 97%, dr: 3:1; catalyzed by Δ-**Rh**: 55.5 mg, yield: 83%, ee: 99%, dr: 4:1). The dr was determined by ^{1}H NMR and the enantiomeric excess established by HPLC analysis using a Chiralpak AD-H column (HPLC: AD-H, 254 nm, *n*-hexane/isopropanol = 95:5, flow rate 0.5 mL/min, 40 °C, t_r (minor) = 26.4 min, t_r (major) = 29.5 min); $[\alpha]_D^{20}$ = +13.1° (*c* 1.4, CH_2Cl_2) for 99% ee of **5e**.

^{1}H NMR (300 MHz, $CDCl_3$) δ 7.08 (d, *J* = 0.8 Hz, 1H), 6.99 (s, 1H), 3.96 (s, 3H), 3.22 (dd, *J* = 16.6, 10.3 Hz, 1H), 3.08-2.97 (m, 1H), 2.74 (dd, *J* = 16.6, 2.6 Hz, 1H), 2.50-2.30 (m, 2H), 2.21-2.06 (m, 1H), 2.01-1.85 (m, 3H), 1.42 (s, 9H), 0.94 (d, *J* = 6.7 Hz, 3H).

^{13}C NMR (75 MHz, $CDCl_3$) δ 214.5, 191.2, 169.2, 143.2, 128.9, 127.0, 82.0, 65.8, 41.7, 38.6, 36.2, 32.4, 29.4, 27.9, 19.4, 16.4.

IR (film): *v* (cm^{-1}) 3112, 2970, 2868, 1743, 1715, 1673, 1462, 1405, 1368, 1283, 1246, 1151, 1125, 1005, 979, 914, 834, 775, 695, 589, 549, 434.

HRMS (ESI, *m*/*z*) calcd for $C_{18}H_{27}N_2O_4$ $[M+H]^+$: 335.1965, found: 335.1964.

(*R*)-*tert*-Butyl 2-((*R*)-4-(1-methyl-1*H*-imidazol-2-yl)-4-oxobutan-2-yl)-1-oxo-2,3-di-hydro-1*H*-indene-2-carboxylate (5f)

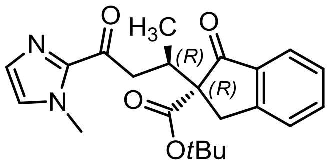

Starting from **4** (30.2 mg, 0.20 mmol) and *tert*-butyl 1-oo-2,3-dihydro-1*H*-indene-2-carboxylate (92.9 mg, 0.40 mmol) according to the general procedure to give **5f** (major product) as a colorless oil (catalyzed by Δ-**Ir**: 68.1 mg, yield: 89%, ee: 97%, dr: 10:1; catalyzed by Δ-**Rh**: 70.4 mg, yield: 92%, ee: 96%, dr: 14:1). Enantiomeric excess established by HPLC analysis using a Chiralpak AD-H column (HPLC: AD-H, 254 nm, *n*-hexane/isopropanol = 97:3, flow rate 0.5 mL/min, 25 °C, t_r (major) = 84.2 min, t_r (minor) = 168.0 min); $[\alpha]_D^{20}$ = –96.9° (*c* 0.7, CH_2Cl_2) for 97% ee of **5f**. The dr value was determined by ^{1}H NMR as shown below. The relative configuration was assigned by X-ray crystallography.

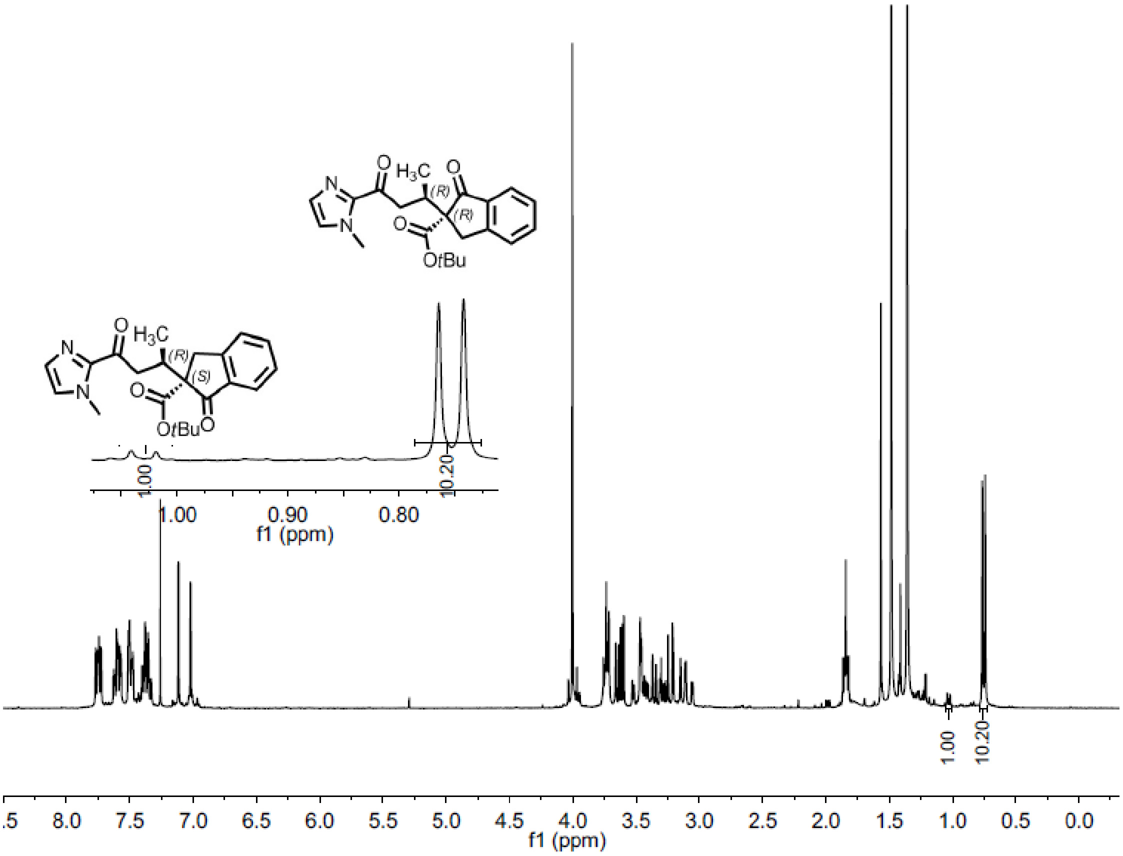

Figure S1. ^{1}H NMR of the crude product (*R*,*R*)-**5f** and its diastereomer from an iridium-catalyzed reaction. Calculated dr = 10:1 according to relative areas of integration.

^{1}H NMR (300 MHz, $CDCl_3$) δ 7.74 (d, J = 7.6 Hz, 1H), 7.65-7.56 (m, 1H), 7.50 (d, J = 7.7 Hz, 1H), 7.40-7.32 (m, 1H), 7.13 (d, J = 0.9 Hz, 1H), 7.03 (s, 1H), 4.01 (s, 3H), 3.69 (d, J = 17.5 Hz, 1H), 3.51-3.39 (m, 1H), 3.32-3.18 (m, 2H), 3.09 (dd, J = 16.3, 2.8 Hz, 1H), 1.36 (s, 9H), 0.76 (d, J = 6.7 Hz, 3H).

^{13}C NMR (75 MHz, $CDCl_3$) δ 202.4, 191.2, 169.3, 154.1, 143.3, 136.0, 135.2, 128.9, 127.6, 127.0, 126.3, 124.6, 82.1, 65.9, 42.4, 36.3, 33.1, 33.0, 27.8, 15.2.

IR (film): ν (cm^{-1}) 2969, 2930, 1705, 1673, 1604, 1464, 1406, 1369, 1332, 1252, 1213, 1146, 1092, 986, 913, 844, 769, 743, 694, 651, 590, 517, 466.

HRMS (ESI, *m*/*z*) calcd for $C_{22}H_{27}N_2O_4$ $[M+H]^+$: 383.1965, found: 383.1962.

3.2. Asymmetric α-Aminations

General Procedure (Reactions of Table 2). To a solution of the 2-acyl imidazole **6a-g** (0.20 mmol), synthesized from their respective Weinreb amides,[9] in anhydrous *i*PrOH (0.10 mL, 2.0 M) was added metal catalyst Λ-**Ir** (3.7 mg, 0.0040 mmol, 2 mol%) or Λ-**Rh** (0.1-1 mol%) in a brown glass vial. After being stirred at room temperature for 30 min, dibenzyl azodicarboxylate (125.5 mg, 0.40 mmol, 2.0 eq, purity of ≥95%) was added. The reaction was stirred at 20 °C, resulting in a precipitate of the products (*R*)-**7a-f**. After the reaction was completed (monitored by TLC), the precipitate was isolated by centrifugation and washed with Et_2O/*n*-hexane (v/v = 1:4) until the filtrate was almost colorless to give the white solid products (*R*)-**7a-f**. In the case of product (*R*)-**7g** formation, after the 2-acyl imidazole **6g** was consumed completely (monitoring by TLC), the mixture was diluted with CH_2Cl_2 and directly subjected to flash silica gel column chromatography with EtOAc/*n*-hexane = 1:2 to afford the product (*R*)-**7g** as a colorless oil. Enantiomeric excess values were determined by chiral HPLC chromatography.

Procedure for Determination of Initial Rates. To a solution of the 2-acyl imidazole **6a** (0.20 mmol) in anhydrous *i*PrOH (0.10 mL, 2.0 M) was added metal catalyst *rac*-**Ir** (1.84 mg, 0.0020 mmol, 1 mol%) or *rac*-**Rh** (1.66 mg, 0.0020 mmol, 1 mol%) in a brown glass vial. After being stirred at room temperature for 30 min, dibenzyl azodicarboxylate (125.5 mg, 0.40 mmol, 2.0 eq, purity ≥95%) was added. The reaction was stirred at 20 °C and conversions were determined by ^{1}H NMR analysis of the crude mixture using mesitylene as an internal standard.

rac-**Ir** (1 mol%): 35 min => 15% conversion, 90 min => 34% conversion.
rac-**Rh** (1 mol%): 2 min => 18% conversion, 5 min => 39% conversion.
Result: ratio of initial rates rhodium catalysis / iridium catalysis = 21.

(*R*)-Dibenzyl 1-(2-(1-methyl-*1H*-imidazol-2-yl)-2-oxo-1-phenylethyl)hydrazine-1,2-dicarboxylate (7a)

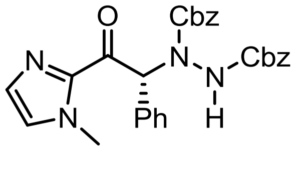

Starting from **6a** (40.0 mg, 0.20 mmol) and dibenzyl azodicarboxylate (125.5 mg, 0.40 mmol, 2.0 eq) according to the general procedure to give (*R*)-**7a** as a white solid. Catalyzed by Λ-**Ir** (3.7 mg, 0.0040 mmol, 2 mol%): 85.7 mg, yield: 86%, *ee*: 92% (crude)/>99.5% (isolated); catalyzed by Λ-**Rh** (0.33 mg, 0.40 µmol, 0.2 mol%): 87.6 mg, yield: 88%, *ee*: 96% (crude)/>99.5% (isolated); catalyzed by Λ-**Rh** (0.17 mg, 0.20 µmol, 0.1 mol%): 82.7 mg, yield: 83%, *ee*: 94% (crude)/>99.5% (isolated). Enantiomeric excess established by HPLC analysis using a Chiralpak AD-H column (λ = 254 nm, eluents: *n*-hexane/isopropanol = 60:40, flow rate: 1.0 mL/min, 25 °C, t_r (major) = 15.3 min, t_r (minor) = 20.4 min); $[\alpha]_D^{20}$ = – 174° (c = 0.4, $CHCl_3$) for (*R*)-**7a** (>99.5% *ee*).

^{1}H NMR (500 MHz, $CDCl_3$) δ 7.47-7.35 (m, 2H), 7.33-7.13 (m, 12H), 7.12-6.90 (m, 5H), 5.34-4.42 (m, 4H), 4.03-3.73 (m, 3H).

^{13}C NMR (126 MHz, $CDCl_3$) δ 189.0, 156.3, 155.1, 141.1, 136.0, 135.9, 132.7, 130.3, 129.8, 128.4, 128.3, 128.0, 127.84, 127.77, 127.5, 127.2, 68.1, 67.8, 66.9, 35.8.

IR (film): ν (cm^{-1}) 3321, 3069, 3032, 2956, 1716, 1681, 1497, 1454, 1402, 1338, 1290, 1218, 1157, 1120, 1080, 1052, 1028, 914, 848, 778, 737, 697, 649.

HRMS (ESI, *m*/*z*) calcd for $C_{28}H_{26}N_4NaO_5$ $[M+Na]^+$: 521.1795, found: 521.1803.

(*R*)-Dibenzyl 1-(2-(1-methyl-*1H*-imidazol-2-yl)-2-oxo-1-o-tolylethyl)hydrazine-1,2-dicarboxylate (7b)

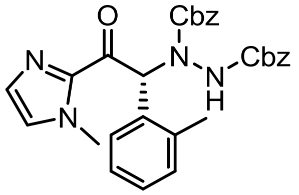

Starting from **6b** (42.8 mg, 0.20 mmol) and dibenzyl azodicarboxylate (125.5 mg, 0.40 mmol, 2.0 eq) according to the general procedure to give (*R*)-**7b** as a white solid. Catalyzed by Λ-**Ir** (3.7 mg, 0.0040 mmol, 2 mol%): 83.0 mg, yield: 81%, *ee*: 91% (crude)/>99.5% (isolated); catalyzed by Λ-**Rh** (0.33 mg, 0.40 µmol, 0.2 mol%): 86.0 mg, yield: 84%, *ee*: 94% (crude)/>99.5% (isolated). Enantiomeric excess established by HPLC analysis using a Chiralpak AD-H column (λ = 254 nm, eluents: *n*-hexane/isopropanol = 60:40, flow rate: 1.0 mL/min, 25 °C, t_r (major) = 16.1 min, t_r (minor) = 17.9 min); $[\alpha]_D^{20}$ = – 126.8° (c = 0.6, $CHCl_3$) for (*R*)-**7b** (>99.5% *ee*).

^{1}H NMR (500 MHz, $CDCl_3$) δ 7.48-6.91 (m, 17H), 6.88 (s, 1H), 5.33-4.30 (m, 4H), 3.93-3.78 (m, 3H), 2.73-1.66 (m, 3H).

^{13}C NMR (126 MHz, $CDCl_3$) δ 190.2, 156.3, 155.1, 141.0, 139.5, 138.7, 135.8, 135.2, 131.4, 130.7, 130.6, 130.0, 129.1, 128.5, 128.2, 128.1, 128.0, 127.9, 127.8, 127.6, 127.5, 127.2, 125.3, 125.0, 68.0, 66.7, 64.0, 35.7, 19.4.

IR (film): ν (cm^{-1}) 3341, 3064, 3031, 2956, 1753, 1716, 1677, 1497, 1455, 1402, 1359, 1292, 1216, 1158, 1119, 1059, 1028, 970, 917, 777, 738, 697, 579.

HRMS (ESI, *m*/*z*) calcd for $C_{29}H_{28}N_4NaO_5$ $[M+Na]^+$: 535.1952, found: 535.1950.

(*R*)-Dibenzyl 1-(1-(4-methoxyphenyl)-2-(1-methyl-*1H*-imidazol-2-yl)-2-oxoethyl)hydrazine-1,2-dicarboxylate (7c)

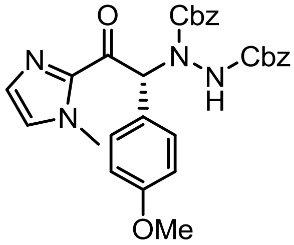

Starting from **6c** (46.0 mg, 0.20 mmol) and dibenzyl azodicarboxylate (125.5 mg, 0.40 mmol, 2.0 eq) according to the general procedure to give (*R*)-**7c** as a white solid. Catalyzed by Λ-**Ir** (3.7 mg, 0.0040 mmol, 2 mol%): 91.8 mg, yield: 87%, *ee*: 95% (crude)/99% (isolated); catalyzed by Λ-**Rh** (0.33 mg, 0.40 μmol, 0.2 mol%): 89.8 mg, yield: 85%, *ee*: 97% (crude)/99% (isolated). Enantiomeric excess established by HPLC analysis using a Chiralpak AD-H column (λ = 254 nm, eluents: *n*-hexane/isopropanol = 60:40, flow rate: 1.0 mL/min, 25 °C, t_r (major) = 20.6 min, t_r (minor) = 28.7 min); $[\alpha]_D^{20}$ = – 155° (c = 1.0, $CHCl_3$) for (*R*)-**7c** (99% *ee*).

^{1}H NMR (500 MHz, $CDCl_3$) δ 7.46 (br, 1H), 7.37-7.12 (m, 10H), 7.13-6.84 (m, 5H), 6.84-6.66 (m, 2H), 5.31-4.55 (m, 4H), 4.01-3.55 (m, 6H).

^{13}C NMR (126 MHz, $CDCl_3$) δ 189.0, 159.6, 156.2, 155.5, 141.0, 135.7, 131.7, 129.8, 128.3, 128.1, 127.9, 127.8, 127.7, 127.5, 127.2, 124.4, 113.8, 68.0, 67.0, 66.8, 55.0, 35.9.

IR (film): ν (cm^{-1}) 3322, 3032, 2957, 1716, 1679, 1609, 1513, 1454, 1402, 1289, 1249, 1178, 1116, 1081, 1029, 916, 753, 697.

HRMS (ESI, *m*/*z*) calcd for $C_{29}H_{28}N_4NaO_6[M+Na]^+$: 551.1901, found: 551.1902.

(*R*)-Dibenzyl 1-(1-(4-chlorophenyl)-2-(1-methyl-*1H*-imidazol-2-yl)-2-oxoethyl)hydrazine-1,2-dicarboxylate (7d)

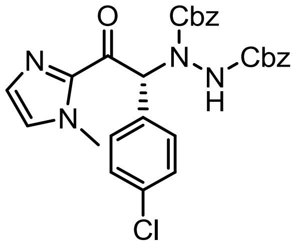

Starting from **6d** (46.8 mg, 0.20 mmol) and dibenzyl azodicarboxylate (125.5 mg, 0.40 mmol, 2.0 eq) according to the general procedure to give (*R*)-**7d** as a white solid (catalyzed by Λ-**Ir** (3.7 mg, 0.0040 mmol, 2 mol%): 87.2 mg, yield: 82%, *ee*: 79% (crude)/84% (isolated); catalyzed by Λ-**Rh** (0.83 mg, 0.0010 mmol, 0.5 mol%): 88.3 mg, yield: 83%, *ee*: 95% (crude)/97% (isolated)). Enantiomeric excess established by HPLC analysis using a Chiralpak AD-H column (λ = 254 nm, eluents: *n*-hexane/isopropanol = 60:40, flow rate: 1.0 mL/min, 25 °C, t_r (major) = 16.9 min, t_r (minor) = 21.2 min); $[\alpha]_D^{20}$ = – 152.6° (c = 1.0, $CHCl_3$) for (*R*)-**7d** (97% *ee*).

^{1}H NMR (500 MHz, $CDCl_3$) δ 7.44 (br, 1H), 7.37-6.85 (m, 17H), 5.31-4.60 (m, 4H), 4.02-3.62 (m, 3H).

^{13}C NMR (126 MHz, $CDCl_3$) δ 188.5, 156.2, 155.2, 140.9, 135.7, 134.5, 131.6, 131.4, 130.0, 128.6, 128.4, 128.3, 128.2, 128.1, 128.0, 127.9, 127.8, 127.7, 127.4, 68.3, 67.1, 65.8, 35.9.

IR (film): ν (cm^{-1}) 3313, 3032, 2955, 2924, 1719, 1680, 1492, 1455, 1401, 1356, 1288, 1218, 1157, 1050, 1014, 915, 815, 740, 696.

HRMS (ESI, *m/z*) calcd for $C_{28}H_{25}ClN_4NaO_5$ $[M+Na]^+$: 555.1406, found: 555.1408.

(*R*)-Dibenzyl 1-(2-(1-methyl-*1H*-imidazol-2-yl)-1-(naphthalen-2-yl)-2-oxoethyl)hydrazine-1,2-dicarboxylate (7e)

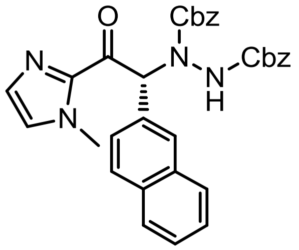

Starting from **6e** (50.0 mg, 0.20 mmol) and dibenzyl azodicarboxylate (125.5 mg, 0.40 mmol, 2.0 eq) according to the general procedure to give (*R*)-**7e** as a white solid. Catalyzed by Λ-**Ir** (3.7 mg,

0.0040 mmol, 2 mol%): 91.0 mg, yield: 83%, *ee*: 90% (crude)/>99.5% (isolated); catalyzed by Λ-**Rh** (0.33 mg, 0.40 μmol, 0.2 mol%): 94.2 mg, yield: 86%, *ee*: 96% (crude)/99% (isolated). Enantiomeric excess established by HPLC analysis using a Chiralpak AD-H column (λ = 254 nm, eluents: *n*-hexane/isopropanol = 60:40, flow rate: 1.0 mL/min, 25 °C, t_r (major) = 21.8 min, t_r (minor) = 31.3 min); $[\alpha]_D^{20}$ = – 167.8° (c = 0.55, $CHCl_3$) for (*R*)-**7e** (>99.5% *ee*).

^{1}H NMR (500 MHz, $CDCl_3$) *δ* 7.86-7.34 (m, 8H), 7.34-7.10 (m, 7H), 7.09-6.93 (m, 3H), 6.90-6.69 (m, 3H), 5.35-4.28 (m, 4H), 3.88 (br s, 3H).

^{13}C NMR (126 MHz, $CDCl_3$) *δ* 188.8, 156.3, 155.3, 141.0, 135.6, 133.0, 132.9, 130.0, 129.8, 129.7, 128.3, 128.2, 128.1, 128.0, 127.9, 127.8, 127.6, 127.3, 126.3, 125.9, 68.2, 66.9, 66.8, 35.8.

IR (film): *ν* (cm^{-1}) 3317, 3031, 2957, 1716, 1678, 1497, 1454, 1402, 1355, 1288, 1217, 1157, 1051, 1015, 976, 916, 817, 754, 696, 556, 478.

HRMS (ESI, *m/z*) calcd for $C_{32}H_{28}N_4NaO_5$ $[M+Na]^+$: 571.1952, found: 571.1958.

(*R*)-Dibenzyl 1-(2-(1-methyl-*1H*-imidazol-2-yl)-1-(thiophen-3-yl)-2-oxoethyl)hydrazine-1,2-dicarboxylate (7f)

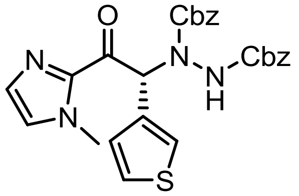

Starting from **6f** (41.2 mg, 0.20 mmol) and dibenzyl azodicarboxylate (125.5 mg, 0.40 mmol, 2.0 eq) according to the general procedure to give (*R*)-**7f** as a white solid. Catalyzed by Λ-**Ir** (3.7 mg, 0.0040 mmol, 2 mol%): 71.6 mg, yield: 71%, *ee*: 80% (crude)/94% (isolated); catalyzed by Λ-**Rh** (0.33 mg, 0.40 μmol, 0.2 mol%): 64.5 mg, yield: 64%, *ee*: 90% (crude)/97% (isolated). Enantiomeric excess established by HPLC analysis using a Chiralpak AD-H column (λ = 254 nm, eluents: *n*-hexane/isopropanol = 60:40, flow rate: 1.0 mL/min, 25 °C, t_r (major) = 18.0 min, t_r (minor) = 22.9 min); $[\alpha]_D^{20}$ = – 92.95° (c = 2.1, $CHCl_3$) for (*R*)-**7f** (94% *ee*).

^{1}H NMR (500 MHz, $CDCl_3$) *δ* 7.58 (br, 1H), 7.45-6.87 (m, 16H), 5.34-4.57 (m, 4H), 4.06-3.65 (m, 3H).

^{13}C NMR (126 MHz, $CDCl_3$) *δ* 188.5, 155.6, 155.2, 140.9, 135.9, 135.7, 132.3, 129.9, 128.9, 128.4, 128.3, 128.1, 127.9, 127.7, 127.6, 127.4, 126.9, 126.6, 125.4, 68.1, 67.1, 63.5, 35.9.

IR (film): ν (cm^{-1}) 3320, 3109, 3032, 2955, 2922, 1899, 1720, 1679, 1497, 1454, 1402, 1356, 1288, 1218, 1157, 1081, 1051, 978, 914, 841, 778, 739, 697, 558.

HRMS (ESI, *m/z*) calcd for $C_{26}H_{24}N_4NaO_5S$ [M+Na]$^+$: 527.1360, found: 527.1361.

(*R*)-Dibenzyl 1-(1-(1-methyl-*1H*-imidazol-2-yl)-1-oxopropan-2-yl)hydrazine-1,2-dicarboxylate (7g)

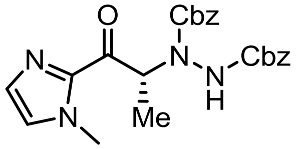

Starting from **6g** (27.6 mg, 0.20 mmol) and dibenzyl azodicarboxylate (125.5 mg, 0.40 mmol, 2.0 eq) according to the general procedure to give (*R*)-**7g** as a colorless oil. Catalyzed by Λ-**Ir** (3.7 mg, 0.0040 mmol, 2 mol%): 74.1 mg, yield: 85%, *ee*: 91%; catalyzed by Λ-**Rh** (1.66 mg, 0.0020 mmol, 1 mol%): 82.8 mg, yield: 95%, *ee*: 92%). Enantiomeric excess established by HPLC analysis using a Chiralpak AD-H column (λ = 254 nm, eluents: *n*-hexane/isopropanol = 60:40, flow rate: 1.0 mL/min, 25 °C, t_r (minor) = 15.2 min, t_r (major) = 19.5 min); $[\alpha]_D^{20}$ = – 6.2° (c = 1.1, $CHCl_3$) for (*R*)-**7g** (91% *ee*).

^{1}H NMR (500 MHz, $CDCl_3$) δ 7.82 (br, 1H), 7.40-6.99 (m, 11H), 6.94 (s, 1H), 5.68-5.59 (m, 1H), 5.29-4.88 (m, 4H), 3.82-3.62 (m, 3H), 1.65-1.45 (m, 3H).

^{13}C NMR (126 MHz, $CDCl_3$) δ 191.1, 156.3, 155.9, 140.0, 135.6, 129.1, 128.1, 128.0, 127.9, 127.7, 127.0, 67.5, 67.0, 60.5, 35.5, 14.6.

IR (film): ν (cm^{-1}) 3310, 3032, 2955, 1717, 1681, 1498, 1455, 1403, 1358, 1294, 1219, 1156, 1048, 1027, 969, 911, 778, 740, 697, 629.

HRMS (ESI, *m/z*) calcd for $C_{23}H_{24}N_4NaO_5$ [M+Na]$^+$: 459.1639, found: 459.1633.

4. Assignment of Absolute Configurations

The absolute configurations of the asymmetric Michael reactions were assigned by comparison of the optical rotations of product **5a** with literature values.[3] The absolute configurations of the α-amination products were assigned by converting product **7g** to the corresponding reported alcohol **S3** and comparing the optical rotation of this material with the literature value.[10]

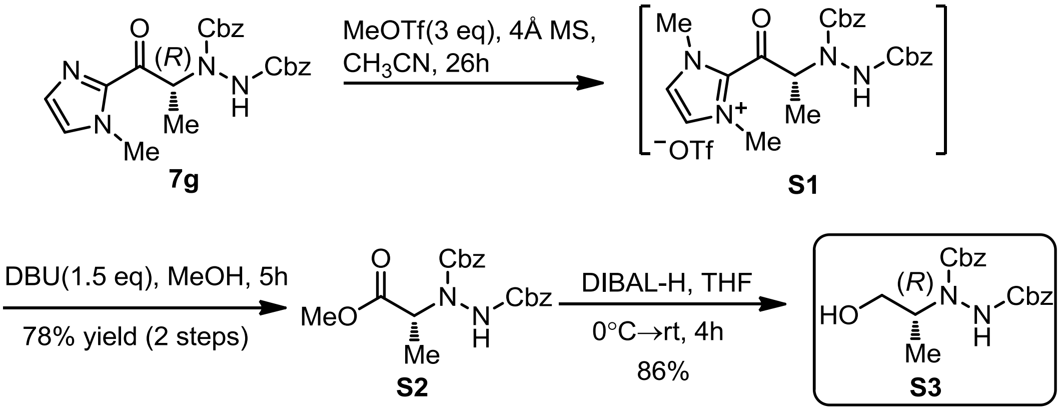

$[\alpha]_D^{20}$ = –21.3° (c = 0.6, $CHCl_3$) for **S3** with *R* configuration

lit[10a]: $[\alpha]_D^{20}$ = –22.5° (c = 0.4, $CHCl_3$) for *R* configuration

lit[10b]: $[\alpha]_D^{20}$ = –32.0° (c = 0.4, $CHCl_3$) for *R* configuration

Conversion of 2-acylimidazole 7g to ester S2.[11] To a solution of **7g** (265.2 mg, 0.607 mmol) in CH_3CN (6.0 mL, 0.1 M, freshly distilled over CaH_2) was added 4 Å MS (300 mg, 50 mg/0.1 mmol of **7g**) under argon atmosphere. The suspension was stirred vigorously under a positive pressure of argon for 2 h at room temperature, then MeOTf (206 µL, 1.82 mmol, 3.0 eq) was added and stirred at room temperature for 26 h. After that, methanol (1.5 mL) and DBU (136 µL, 0.91 mmol, 1.5 eq) were added successively at room temperature. After stirring at room temperature for 1 h, the solvent was evaporated and the residue was directly purified by flash silica chromatography with EtOAc/*n*-hexane = 1:10 to 1:8 to afford the **S2** (182.8 mg, 0.473 mmol, 78%) as a colorless oil. HRMS (ESI, *m*/*z*) calcd for $C_{20}H_{22}N_2NaO_6$ $[M+Na]^+$: 409.1370, found: 409.1370.

Reduction of ester S2 to alcohol S3. A solution of diisobutylaluminum hydride (0.875 mmol, 1.0 M in *n*-hexane) was added to a cold (0 °C) solution of **S2** (135 mg, 0.35 mmol) in THF (1.4 mL, 0.25 M) under argon atmosphere. The resulting mixture was stirred continuously for an additional 3 h. The mixture was quenched with an aqueous solution of saturated sodium tartrate and stirred

vigorously for 30 min at room temperature. The reaction mixture was extracted with EtOAc (3 x 5 mL). The combined organic layers were dried over Na_2SO_4, filtered and concentrated under reduced pressure. The residue was purified by column chromatography on silica gel (EtOAc/*n*-hexane) to afford the pure product **S3** (107.7 mg, 0.30 mmol, 86%) as a colorless oil.

^{1}H NMR (400 MHz, $CDCl_3$) δ 7.43-7.21 (m, 10H), 6.65 (s, 1H), 5.42-4.86 (m, 4H), 4.65-3.90 (m, 2H), 3.57-3.30 (m, 2H), 1.00 (d, J = 6.6 Hz, 3H).

$[\alpha]_D^{20}$ = – 21.3° (c = 0.6, $CHCl_3$). (Lit.[10a] $[\alpha]_D^{20}$ = – 22.5° (c = 0.4, $CHCl_3$) for product with *R* configuration). All other spectroscopic data were in agreement with the literature.[10,12]

5. HPLC on Chiral Stationary Phase

5.1. Enantiomeric Purities of the Rhodium Catalysts

The analysis was performed with a Daicel Chiralpak IB (250 × 4.6 mm) HPLC column on an Agilent 1200 Series HPLC System. The column temperature was 25 °C and UV-absorption was measured at 254 nm. Solvent A = 0.1% TFA, solvent B = MeCN.

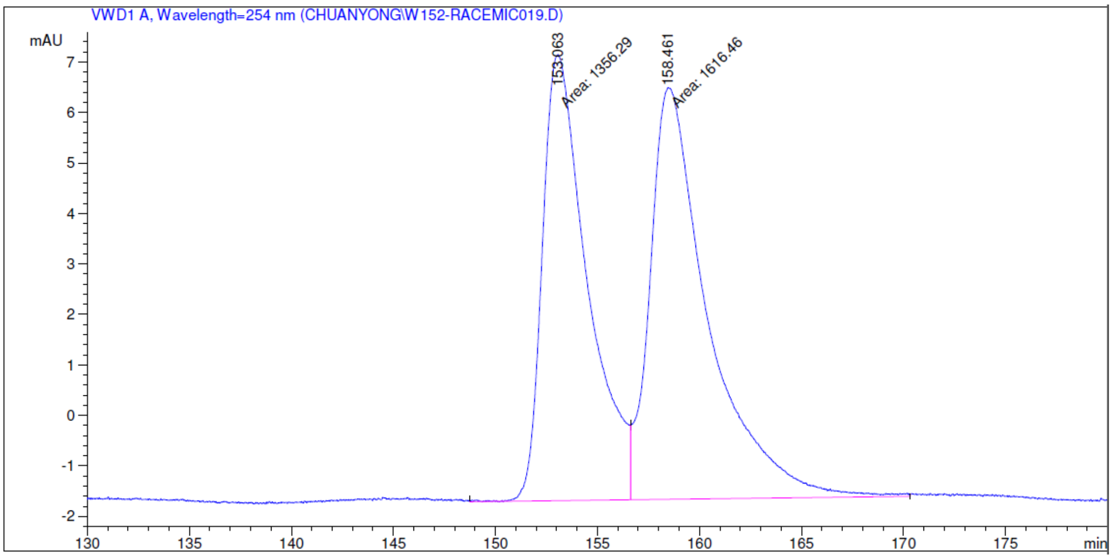

Figure S2. HPLC trace for the racemic reference complexes Δ/Λ-**Rh**. (Daicel Chiralpak IB, with a linear gradient of 30% to 41% B in 60 min, flow rate = 0. 6 mL/min).

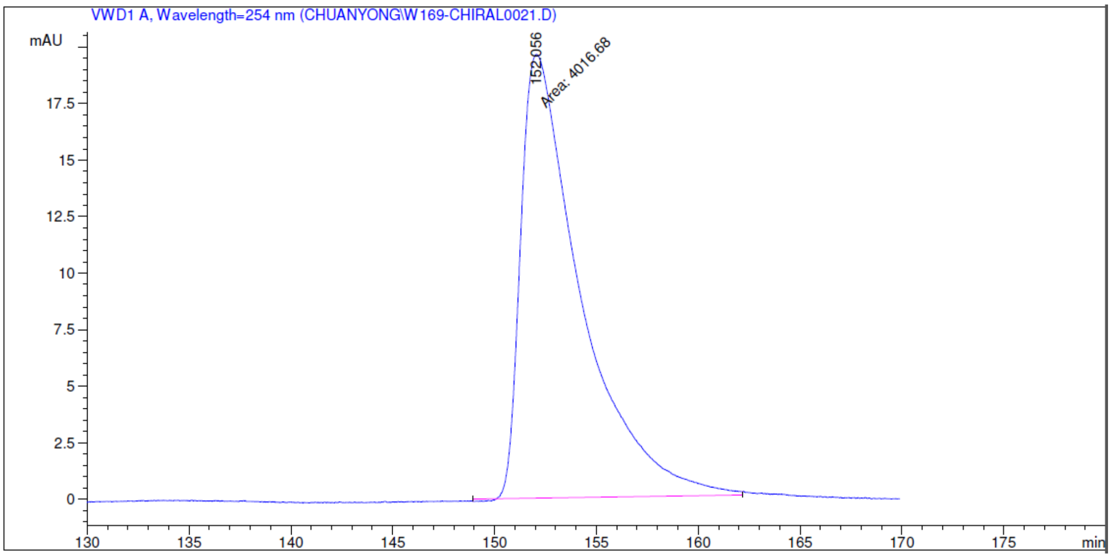

Figure S3. HPLC trace for the complex Δ-**Rh**. Integration of peak areas > 100:1 e.r.

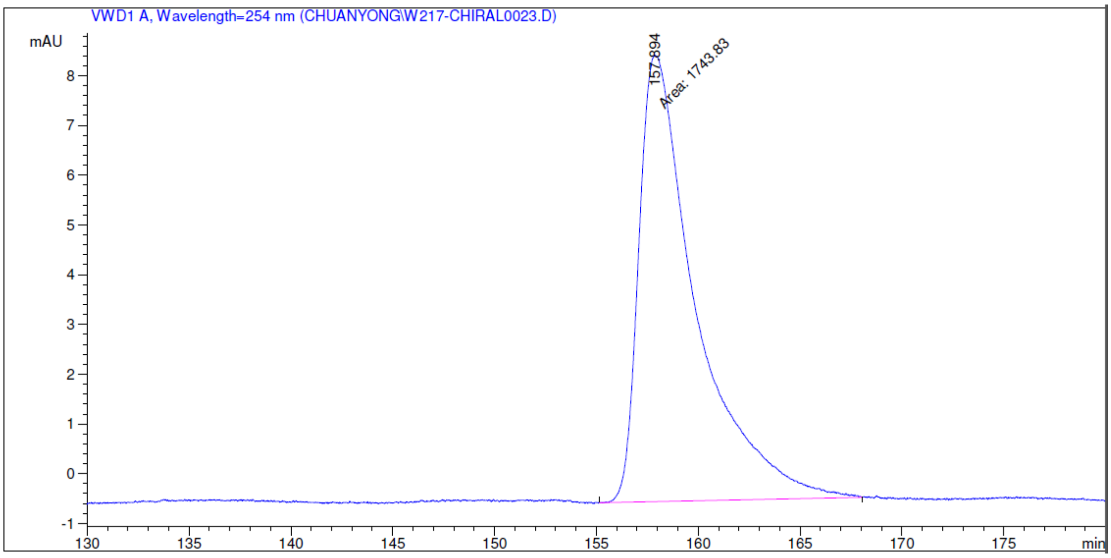

Figure S4. HPLC trace for the complex Λ-**Rh**. Integration of peak areas > 100:1 e.r.

5.2. Enantioselectivities of the Asymmetric Michael Reactions

Enantiomeric purities of the compounds **5a-f** were determined with a Daicel Chiralpak IC or AD-H (250 × 4.6 mm) HPLC column on an Agilent 1200 Series HPLC System using *n*-hexane/isopropanol as mobile phase, column temperature = 40 °C or 25 °C, UV-absorption was measured at 254 nm.

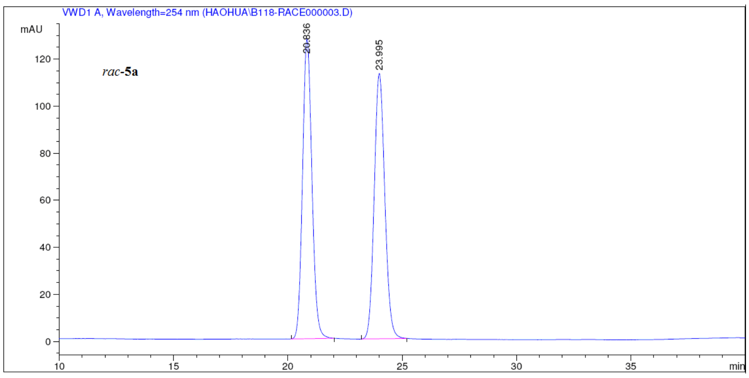

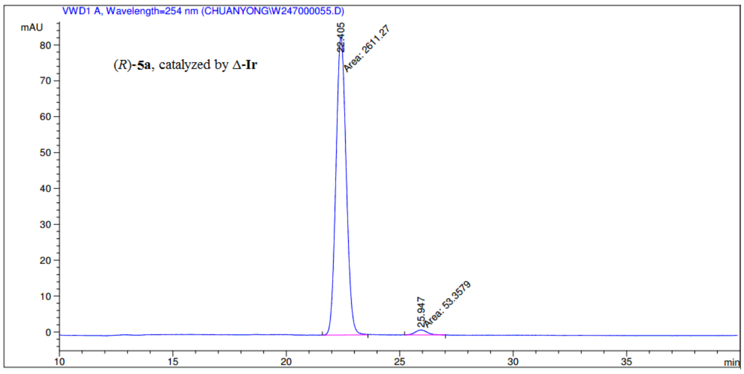

Peak #	RetTime [min]	Type	Width [min]	Area mAU *s	Height [mAU]	Area %
1	22.405	MM	0.5237	2611.27051	83.10204	97.9975
2	25.947	MM	0.6314	53.35791	1.40840	2.0025

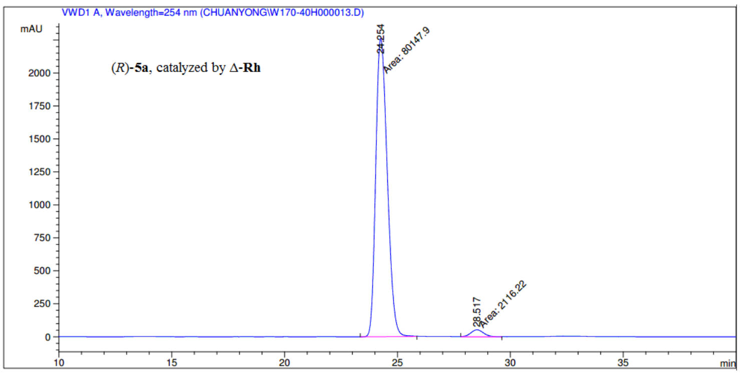

Peak #	RetTime [min]	Type	Width [min]	Area mAU *s	Height [mAU]	Area %
1	24.254	MM	0.5904	8.01479e4	2262.37061	97.4275
2	28.517	MM	0.6659	2116.22437	52.96912	2.5725

Figure S5. HPLC traces of *rac*-**5a** (reference HPLC trace on the top) and (*R*)-**5a**. Area integration = 98.0:2.0 (second trace, 96% ee, catalyzed by Δ-**Ir**) and 97.4:2.6 (third trace, 95% ee, catalyzed by Δ-**Rh**).

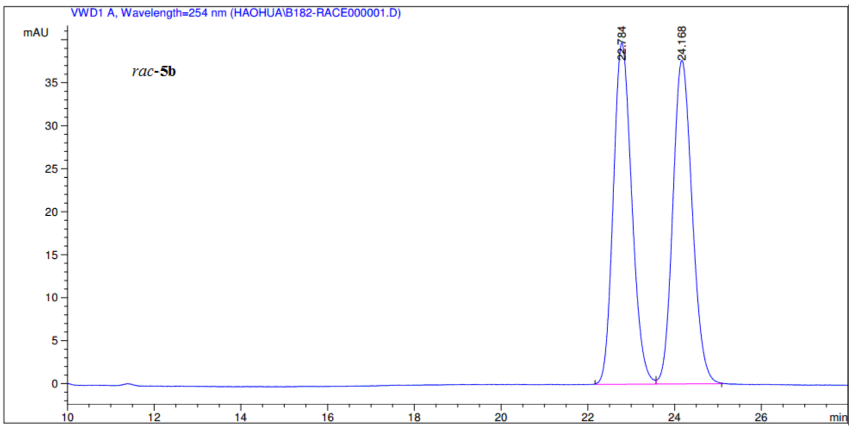

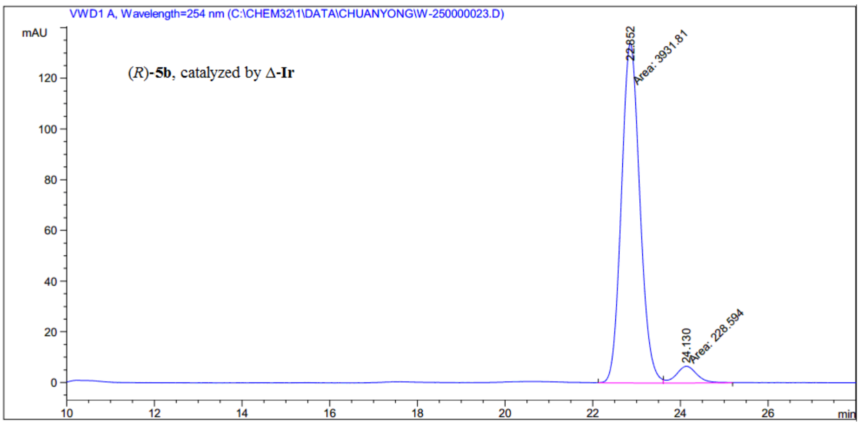

Peak #	RetTime [min]	Type	Width [min]	Area mAU *s	Height [mAU]	Area %
1	22.852	MM	0.4886	3931.80957	134.13013	94.5055
2	24.130	MM	0.5664	228.59448	6.72654	5.4945

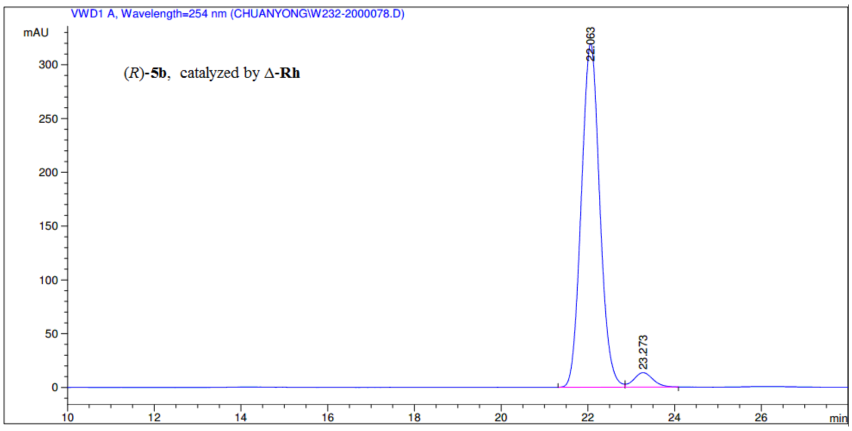

Peak #	RetTime [min]	Type	Width [min]	Area mAU *s	Height [mAU]	Area %
1	22.063	BV	0.4589	9423.82324	319.67722	95.8346
2	23.273	VB	0.4681	409.60535	13.47748	4.1654

Figure S6. HPLC traces of *rac*-**5b** (reference HPLC trace on the top) and (*R*)-**5b**. Area integration = 94.5:5.5 (second trace, 89% ee, catalyzed by Δ-**Ir**) and 95.8:4.2 (third trace, 92% ee, catalyzed by Δ-**Rh**).

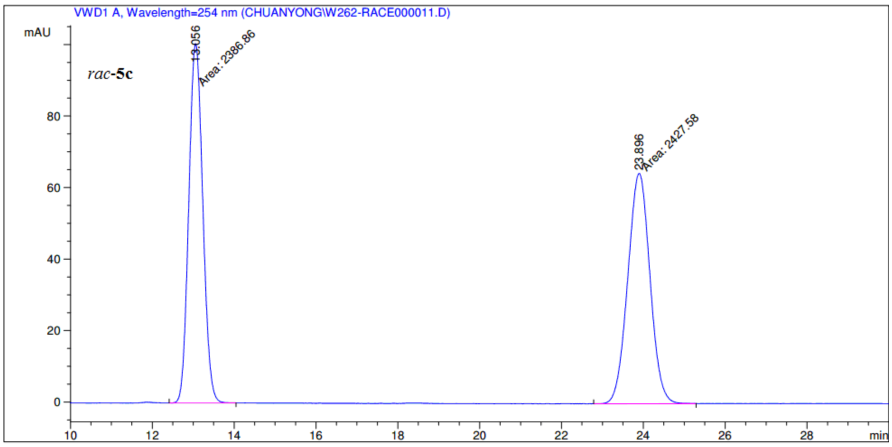

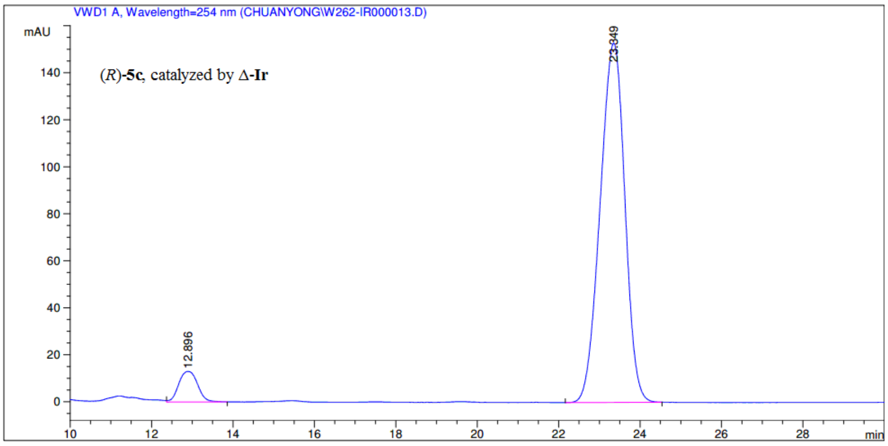

Peak #	RetTime [min]	Type	Width [min]	Area mAU *s	Height [mAU]	Area %
1	12.896	VB	0.5213	421.45984	13.03559	6.1411
2	23.349	VB	0.6663	6441.47266	152.85992	93.8589

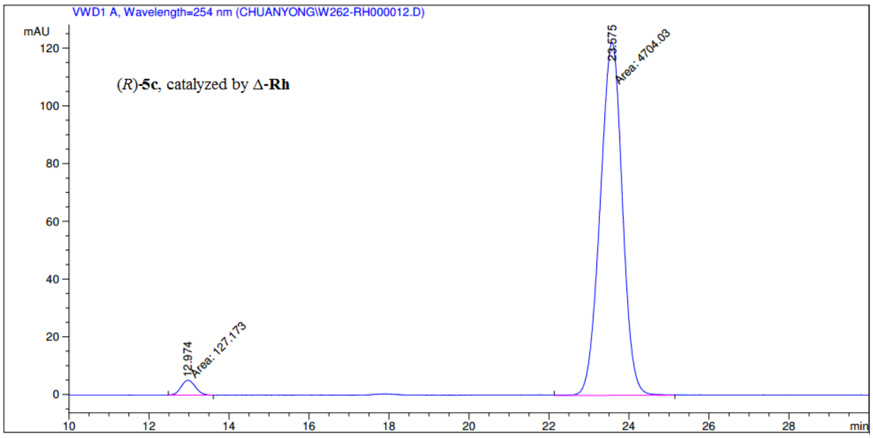

Peak #	RetTime [min]	Type	Width [min]	Area mAU *s	Height [mAU]	Area %
1	12.974	MM	0.4112	127.17268	5.15447	2.6323
2	23.575	MM	0.6412	4704.03027	122.27278	97.3677

Figure S7. HPLC traces of *rac*-**5c** (reference HPLC trace on the top) and (*R*)-**5c**. Area integration = 93.9:6.1 (second trace, 88% ee, catalyzed by Δ-**Ir**) and 97.4:2.6 (third trace, 95% ee, catalyzed by Δ-**Rh**).

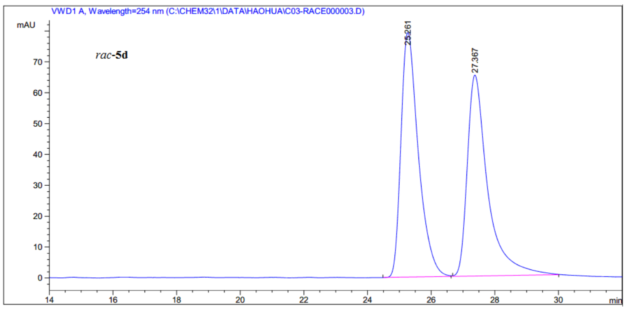

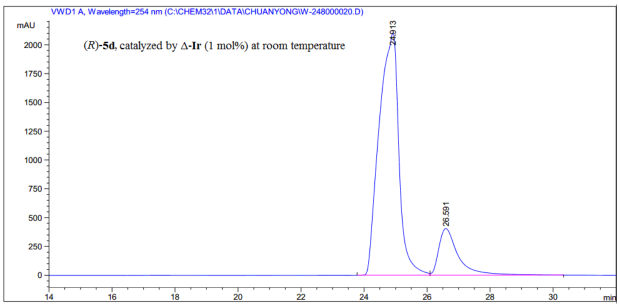

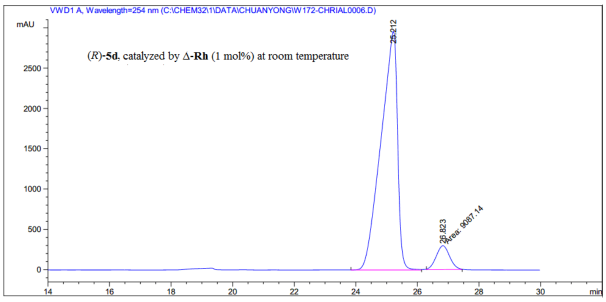

Peak #	RetTime [min]	Type	Width [min]	Area mAU *s	Height [mAU]	Area %
1	24.913	BV	0.5729	9.14997e4	2103.99976	84.0571
2	26.591	VB	0.6404	1.73545e4	404.45508	15.9429

Peak #	RetTime [min]	Type	Width [min]	Area mAU *s	Height [mAU]	Area %
1	25.212	VV	0.5121	1.10204e5	2956.29004	92.3824
2	26.823	MM	0.5095	9087.13965	297.25317	7.6176

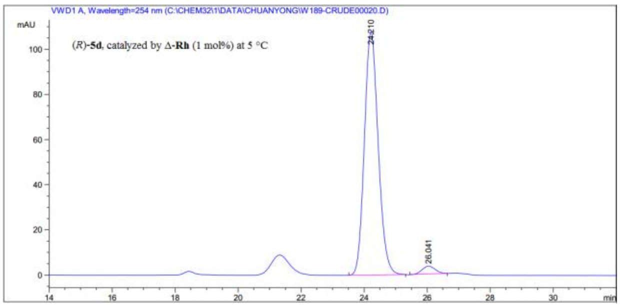

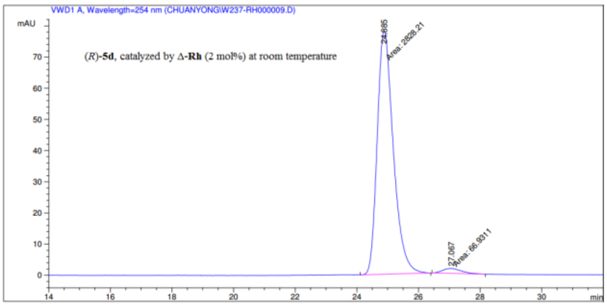

Peak #	RetTime [min]	Type	Width [min]	Area mAU *s	Height [mAU]	Area %
1	24.210	BB	0.4739	3285.07690	107.67587	97.0189
2	26.041	BV	0.4372	100.93921	3.48241	2.9811

Peak #	RetTime [min]	Type	Width [min]	Area mAU *s	Height [mAU]	Area %
1	24.885	MM	0.6044	2828.21021	77.98344	97.6882
2	27.067	MM	0.7059	66.93111	1.58025	2.3118

Figure S8. HPLC traces of *rac*-**5d** (reference HPLC trace on the top) and (*R*)-**5d**. Area integration = 84.1:15.9 (second trace, 68% ee, catalyzed by Δ-**Ir** (1 mol%) at room temperature), 92.4:7.6 (third trace, 85% ee, catalyzed by Δ-**Rh** (1 mol%) at room temperature), 97.0:3.0 (fourth trace, 94% ee, catalyzed by Δ-**Rh** (1 mol%) at 5 °C), 97.7:2.3 (fifth trace, 95% ee, catalyzed by Δ-**Rh** (2 mol%) at room temperature).

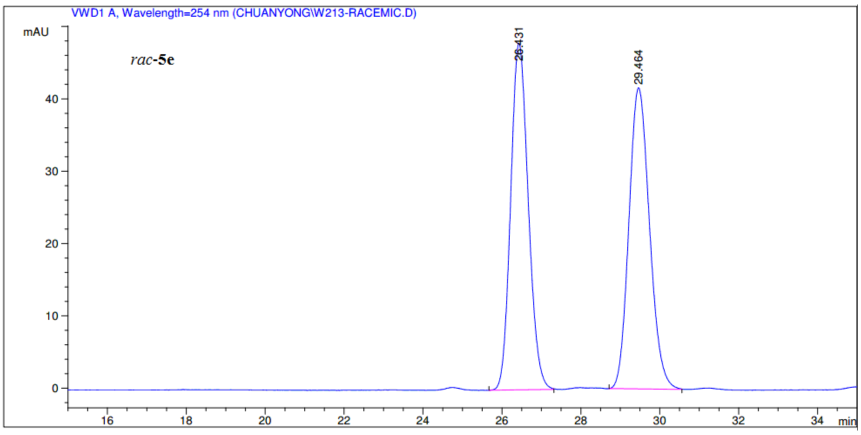

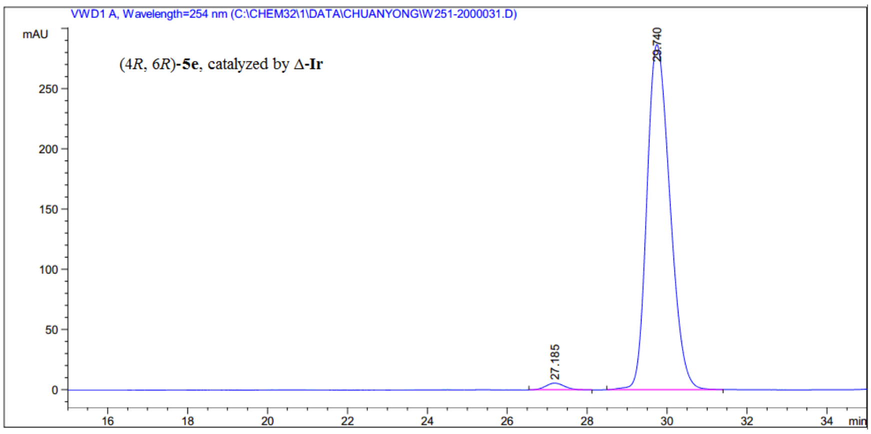

Peak #	RetTime [min]	Type	Width [min]	Area mAU *s	Height [mAU]	Area %
1	27.185	BB	0.5225	183.97090	5.60758	1.5766
2	29.740	BB	0.6306	1.14849e4	286.57001	98.4234

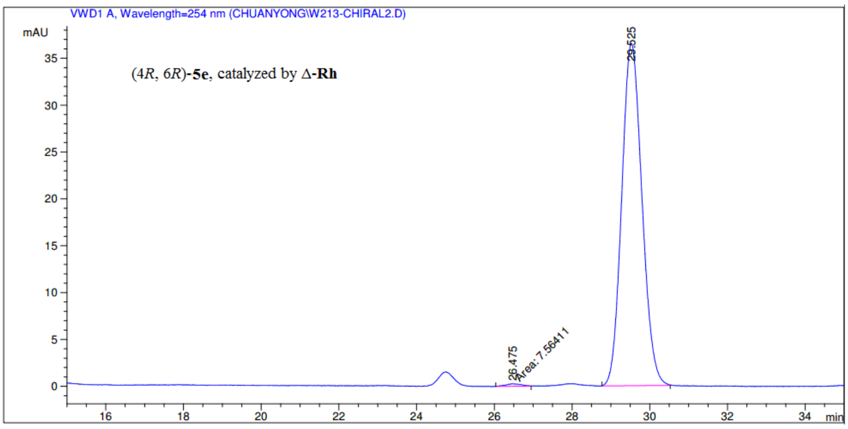

Peak #	RetTime [min]	Type	Width [min]	Area mAU *s	Height [mAU]	Area %
1	26.475	MM	0.4828	7.56411	2.61112e-1	0.5698
2	29.525	BB	0.5615	1319.99036	36.51560	99.4302

Figure S9. HPLC traces of *rac*-**5e** (reference HPLC trace on the top) and (*R*,*R*)-**5e**. Area integration = 98.4:1.6 (second trace, 97% ee, catalyzed by Δ-**Ir**) and 99.4:0.6 (third trace, 99% ee, catalyzed by Δ-**Rh**).

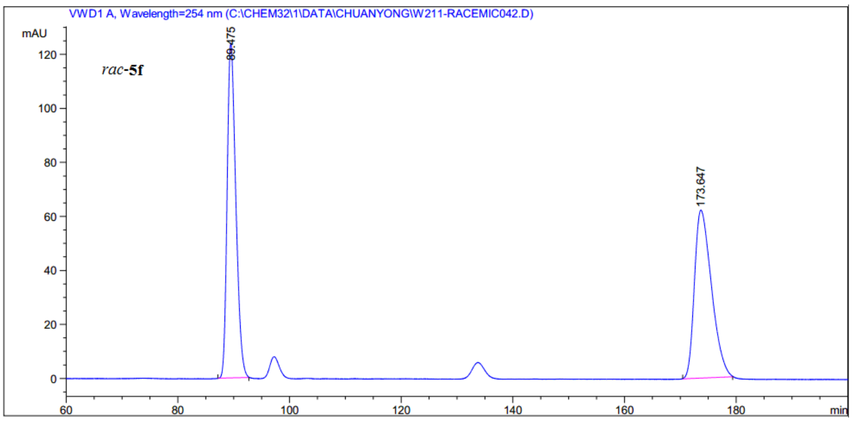

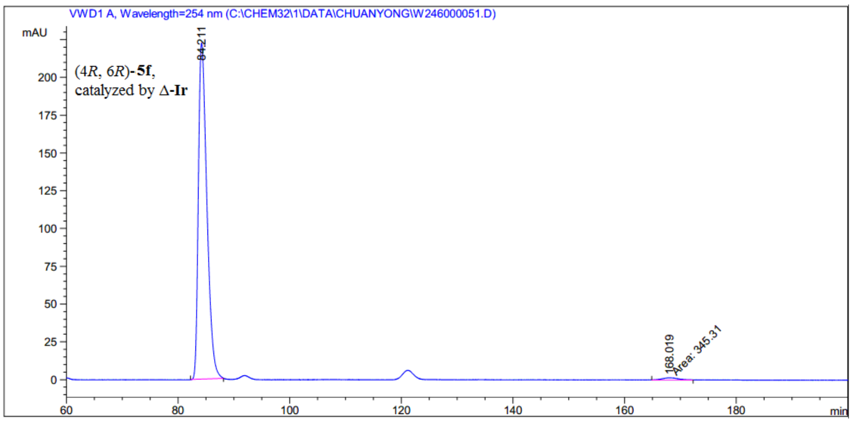

Peak #	RetTime [min]	Type	Width [min]	Area mAU *s	Height [mAU]	Area %
1	84.211	BB	1.5474	2.35205e4	222.56070	98.5531
2	168.019	MM	3.6661	345.30972	1.56983	1.4469

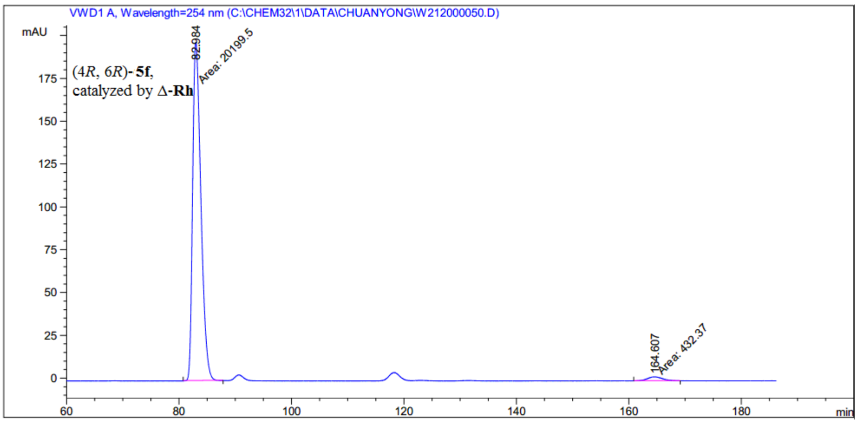

Peak #	RetTime [min]	Type	Width [min]	Area mAU *s	Height [mAU]	Area %
1	82.984	MM	1.7034	2.01995e4	197.64090	97.9044
2	164.607	MM	3.1124	432.37006	2.31528	2.0956

Figure S10. HPLC traces of *rac*-**5f** (reference HPLC trace on the top) and (*R*,*R*)-**5f**. Area integration = 98.6:1.4 (second trace, 97% ee, catalyzed by Δ-**Ir**) and 97.9:2.1 (third trace, 96% ee, catalyzed by Δ-**Rh**).

5.3. Enantioselectivities of the Asymmetric α-Aminations

Enantiomeric purities of the compounds (*R*)-**7a-g** were determined with a Daicel Chiralpak AD-H HPLC column (250 x 4.6 mm) on an Agilent 1260 Series HPLC System using *n*-hexane/isopropanol as the mobile phase. The column temperature was 25 °C and the UV-absorption was measured at 254 nm.

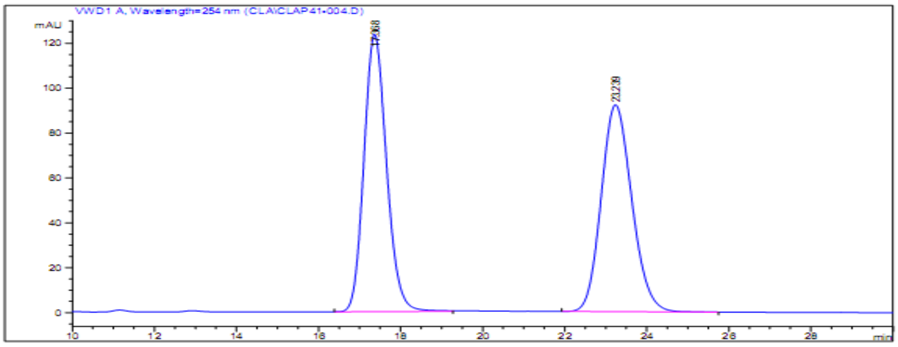

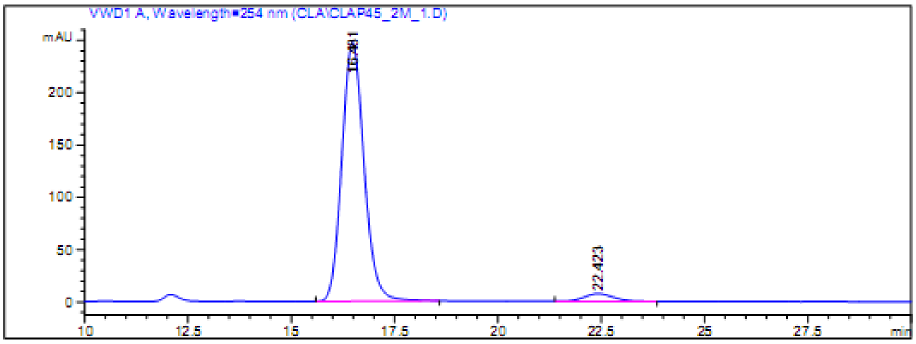

#	[min]		[min]	[mAU*s]	[mAU]	%
1	16.481	MM R	0.6223	9222.84570	247.00887	96.1914
2	22.423	MM R	0.8447	365.17065	7.20489	3.8086

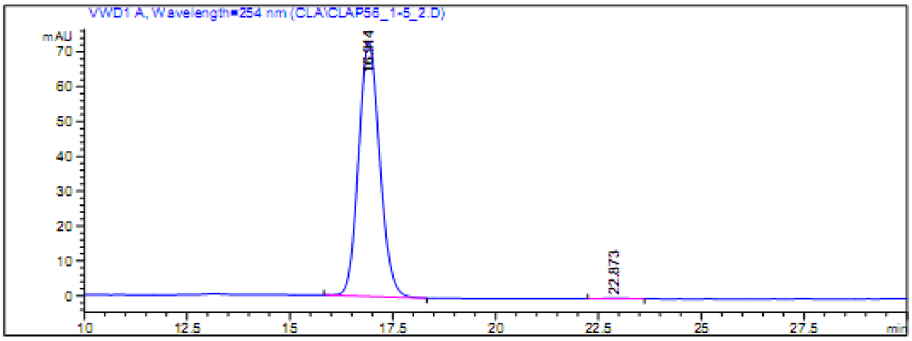

#	[min]		[min]	[mAU*s]	[mAU]	%
1	16.914	BB	0.5543	2606.09473	73.02632	99.7862
2	22.873	MM R	0.7416	5.58342	1.25485e-1	0.2138

Figure S11. HPLC traces of *rac*-**7a** (reference HPLC trace on the top) and (*R*)-**7a**. Area integration = 96.2:3.8 (second trace, 92% *ee* of crude (*R*)-**7a**, catalyzed by Λ-**Ir**) and 99.8:0.2 (third trace, >99.5% *ee* of isolated (*R*)-**7a**, catalyzed by Λ-**Ir**).

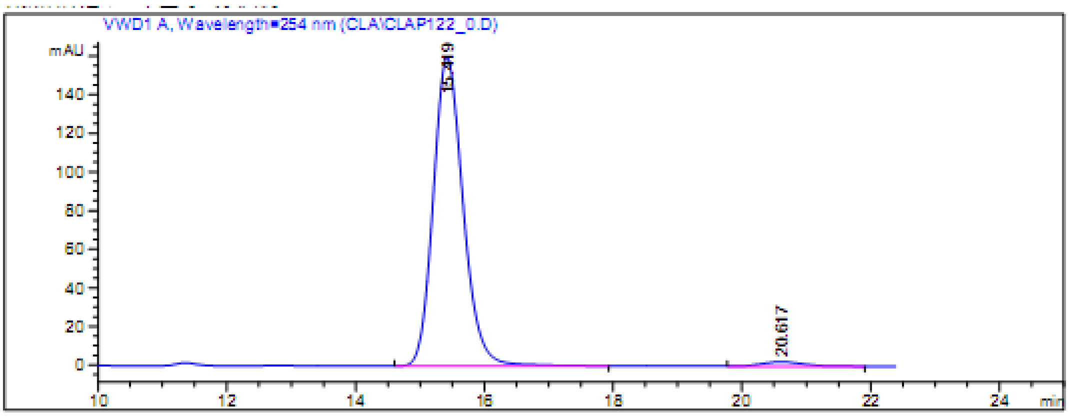

#	[min]		[min]	[mAU*s]	[mAU]	%
1	15.419	MM R	0.5367	5145.90918	159.79317	98.1111
2	20.617	MM R	0.7295	99.07210	2.26355	1.8889

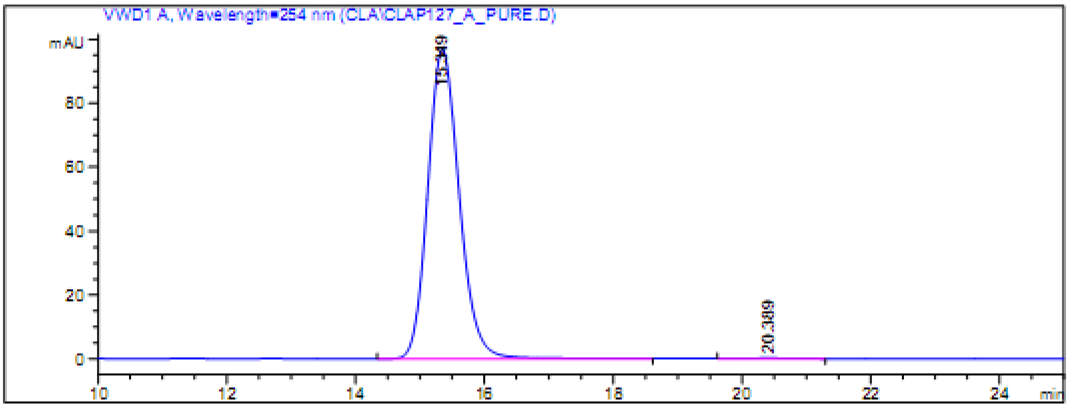

#	[min]		[min]	[mAU*s]	[mAU]	%
1	15.349	MM R	0.5663	3286.77051	96.73617	99.7931
2	20.389	MM R	0.7599	6.81481	1.49477e-1	0.2069

Figure S12. HPLC traces of (*R*)-**7a**. Area integration = 98.1:1.9 (first trace, 96% *ee* of crude (*R*)-**7a**, catalyzed by Λ-**Rh** (0.2 mol%)) and 99.8:0.2 (second trace, >99.5% *ee* of isolated (*R*)-**7a**, catalyzed by Λ-**Rh** (0.2 mol%)).

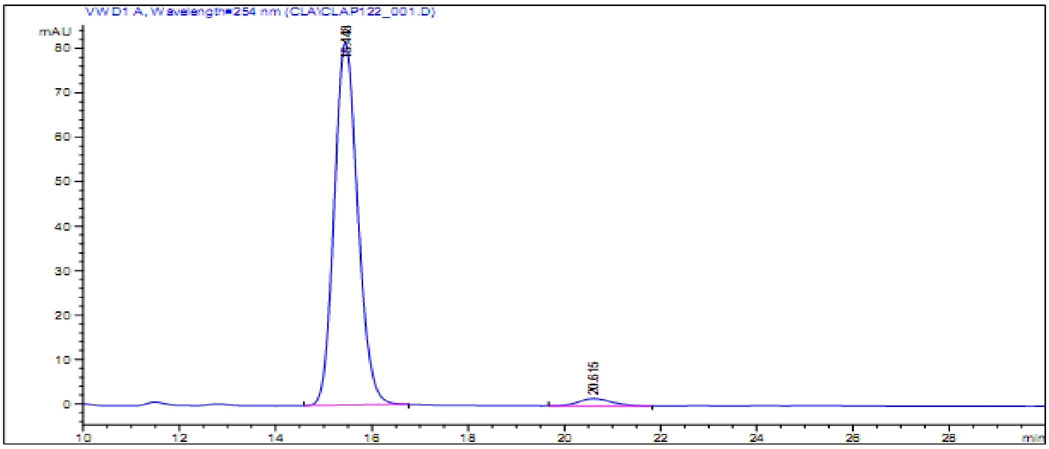

#	[min]		[min]	[mAU*s]	[mAU]	%
1	15.448	MM R	0.5666	2769.51660	81.46921	97.2494
2	20.615	MM R	0.8179	78.33437	1.59632	2.7506

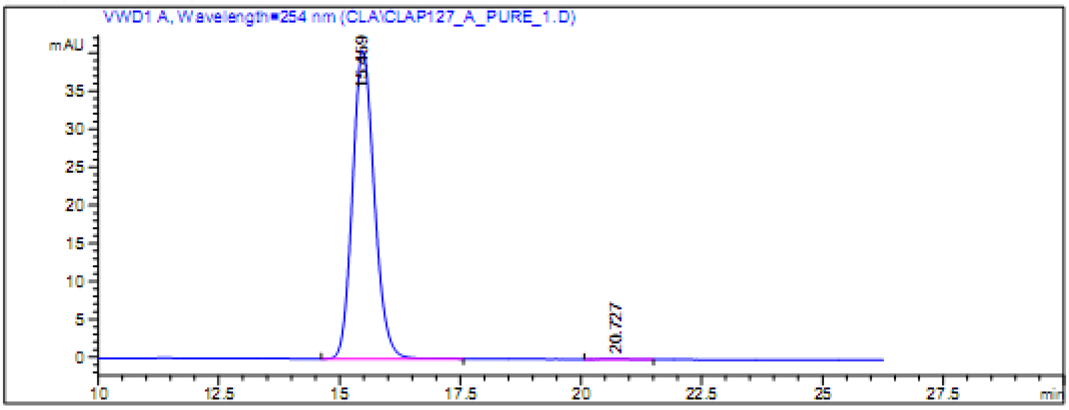

#	[min]		[min]	[mAU*s]	[mAU]	%
1	15.459	MM R	0.9999	1277.69482	21.29672	99.8216
2	20.727	MM R	0.6866	2.28355	5.54276e-2	0.1784

Figure S13. HPLC traces of (*R*)-**7a**. Area integration = 97.2:2.8 (first trace, 94% *ee* of crude (*R*)-**7a**, catalyzed by Λ-**Rh** (0.1 mol%)) and 99.8:0.2 (second trace, >99.5% *ee* of isolated (*R*)-**7a**, catalyzed by **Λ-Rh** (0.1 mol%)).

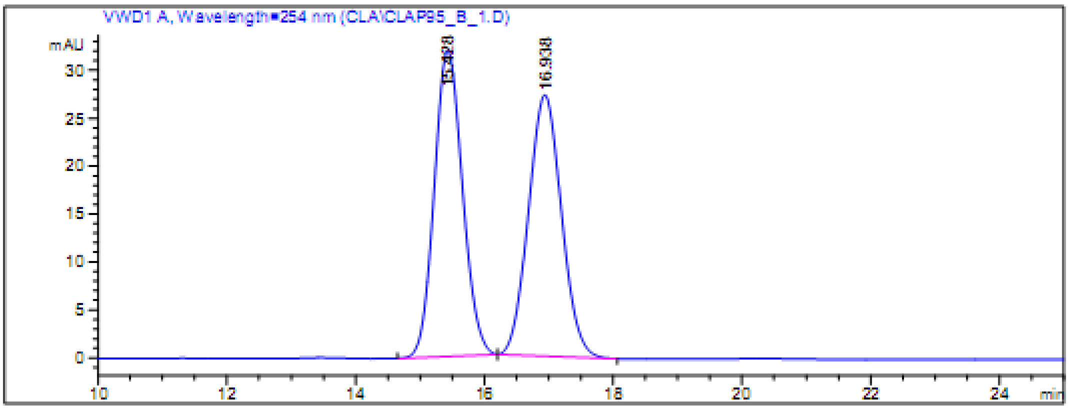

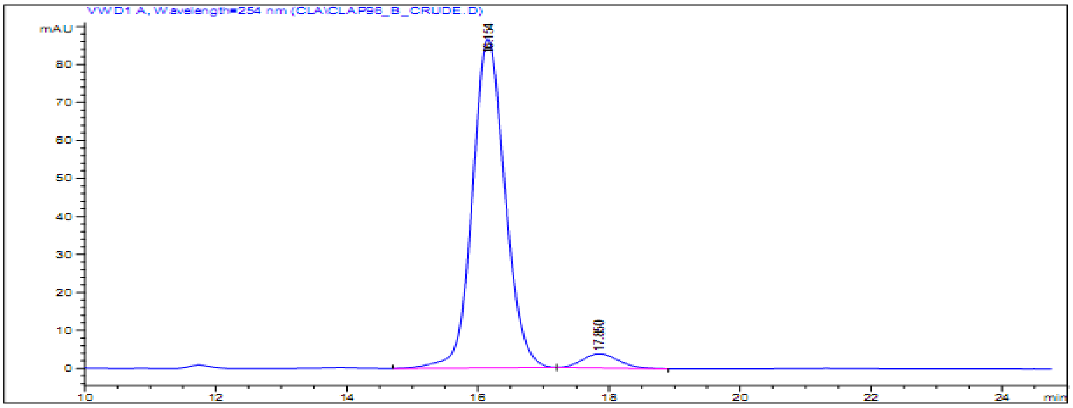

#	[min]		[min]	[mAU*s]	[mAU]	%
1	16.154	BB	0.5309	2986.38867	86.47682	95.5659
2	17.850	BB	0.5738	138.56366	3.66705	4.4341

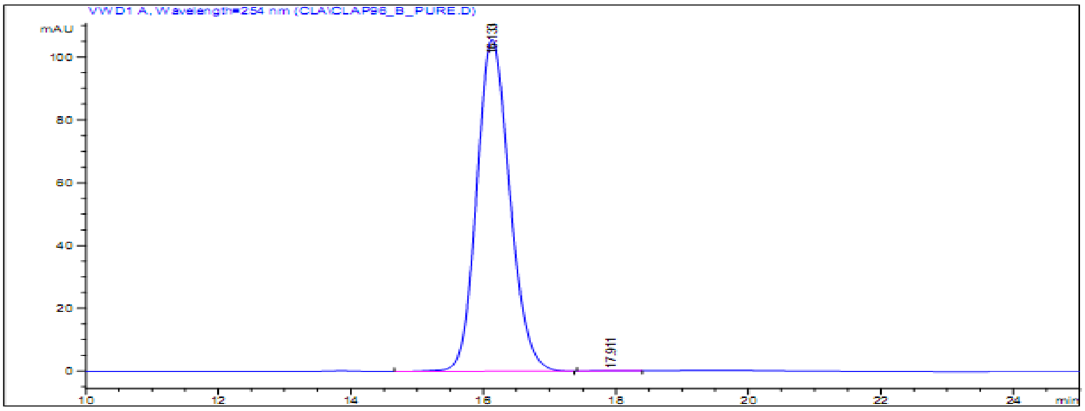

#	[min]		[min]	[mAU*s]	[mAU]	%
1	16.133	BB	0.5349	3657.02344	105.63079	99.8977
2	17.911	MM R	0.5160	3.74470	1.20964e-1	0.1023

Figure S14. HPLC traces of *rac*-**7b** (reference HPLC trace on the top) and (*R*)-**7b**. Area integration = 95.6:4.4 (second trace, 91% *ee* of crude (*R*)-**7b**, catalyzed by **Λ-Ir**) and 99.9:0.1 (third trace, >99.5% *ee* of isolated (*R*)-**7b**, catalyzed by **Λ-Ir**).

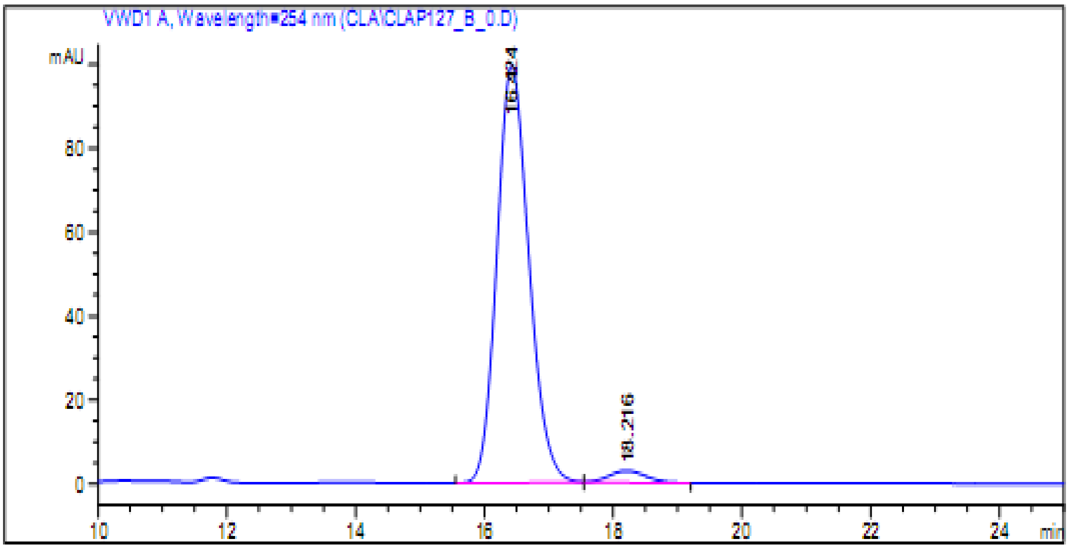

#	[min]		[min]	[mAU*s]	[mAU]	%
1	16.424	MM R	0.5759	3437.16602	99.47052	96.8542
2	18.216	MM R	0.6494	111.63770	2.86528	3.1458

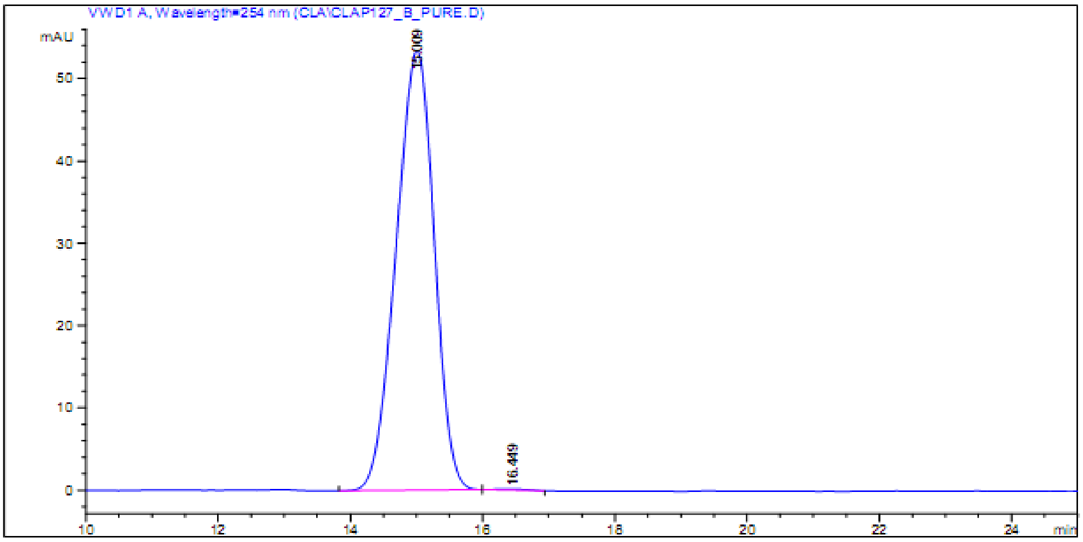

#	[min]		[min]	[mAU*s]	[mAU]	%
1	15.009	MM R	0.6672	2137.31543	53.38872	99.7758
2	16.449	MM R	0.5722	4.80312	1.39892e-1	0.2242

Figure S15. HPLC traces of (*R*)-**7b**. Area integration = 96.9:3.1 (first trace, 94% *ee* of crude (*R*)-**7b**, catalyzed by **Λ-Rh**) and 99.8:0.2 (second trace, >99.5% *ee* of isolated (*R*)-**7b**, catalyzed by **Λ-Rh**).

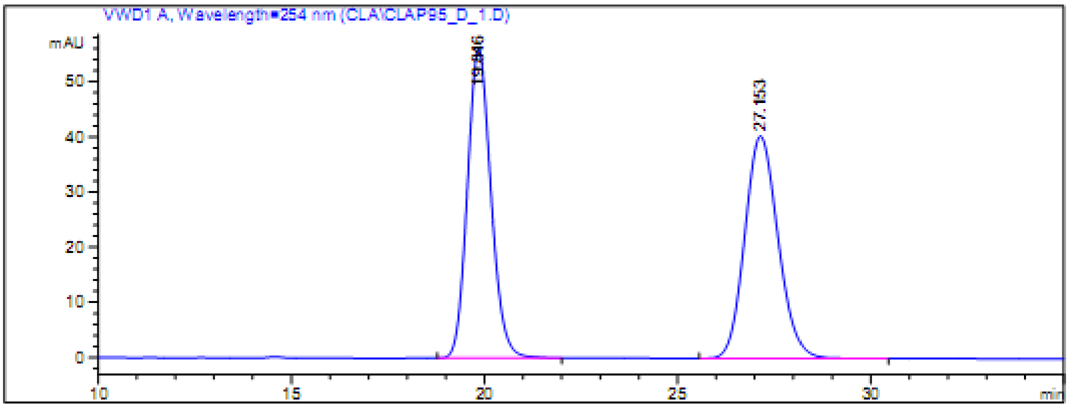

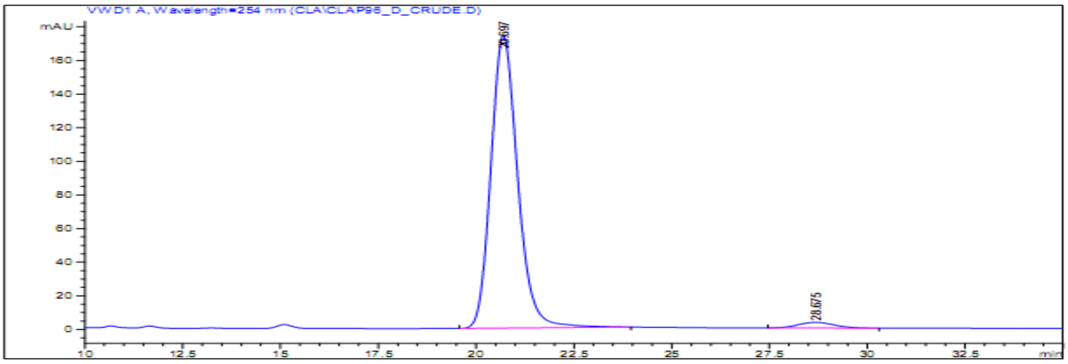

#	[min]		[min]	[mAU*s]	[mAU]	%
1	20.697	MM R	0.7919	8261.02441	173.87050	97.4509
2	28.675	MM R	1.0940	216.08800	3.29211	2.5491

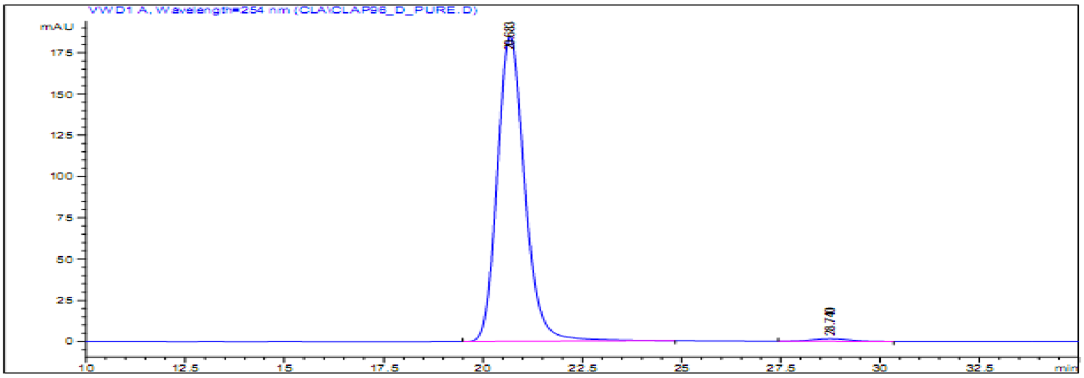

#	[min]		[min]	[mAU*s]	[mAU]	%
1	20.683	MM R	0.8033	8905.40137	184.77211	98.8860
2	28.740	MM R	1.0846	100.32333	1.54163	1.1140

Figure S16. HPLC traces of *rac*-**7c** (reference HPLC trace on the top) and (*R*)-**7c**. Area integration = 97.5:2.5 (second trace, 95% *ee* of crude (*R*)-**7c**, catalyzed by **Λ-Ir**) and 98.9:1.1 (third trace, 99% *ee* of isolated (*R*)-**7c**, catalyzed by **Λ-Ir**).

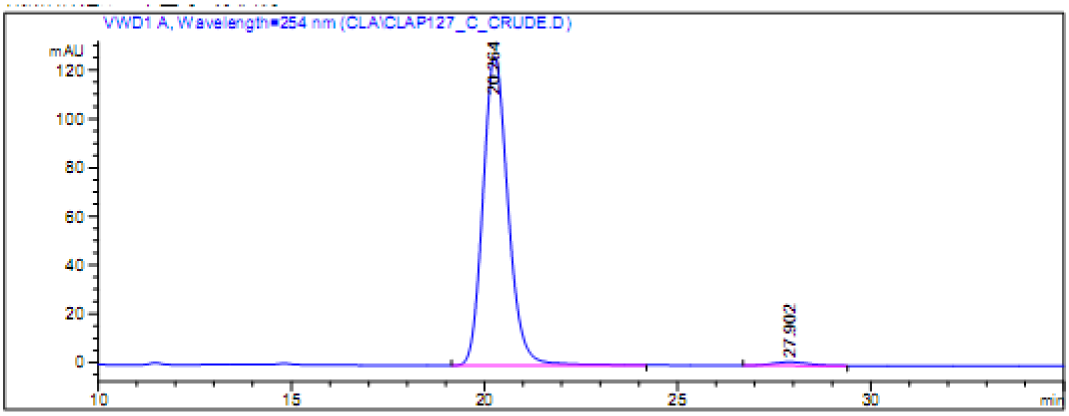

#	[min]		[min]	[mAU*s]	[mAU]	%
1	20.264	MM R	0.7522	5715.81738	126.64493	98.4555
2	27.902	MM R	1.0377	89.66805	1.44017	1.5445

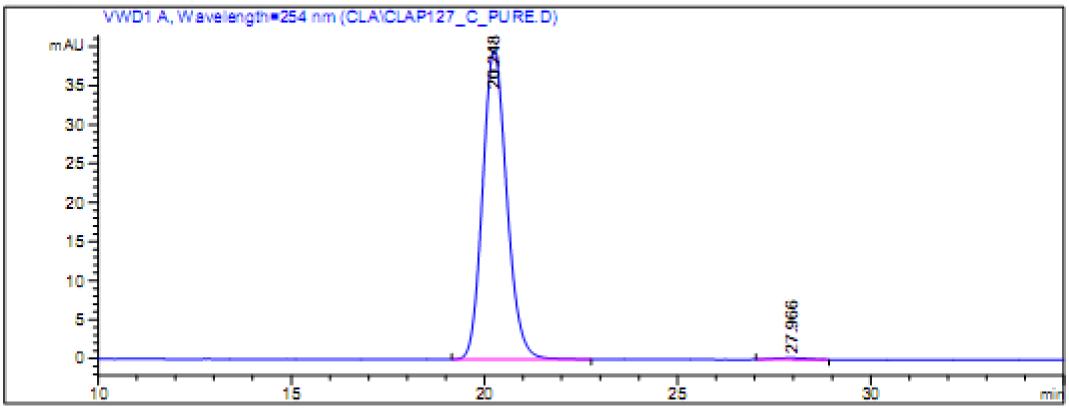

#	[min]		[min]	[mAU*s]	[mAU]	%
1	20.248	BB	0.6746	1723.84216	39.54572	99.3399
2	27.966	MM R	0.9333	11.45464	2.04563e-1	0.6601

Figure S17. HPLC traces of (*R*)-**7c**. Area integration = 98.5:1.5 (first trace, 97% *ee* of crude (*R*)-**7c**, catalyzed by **Λ-Rh**) and 99.3:0.7 (second trace, 99% *ee* of isolated (*R*)-**7c**, catalyzed by **Λ-Rh**).

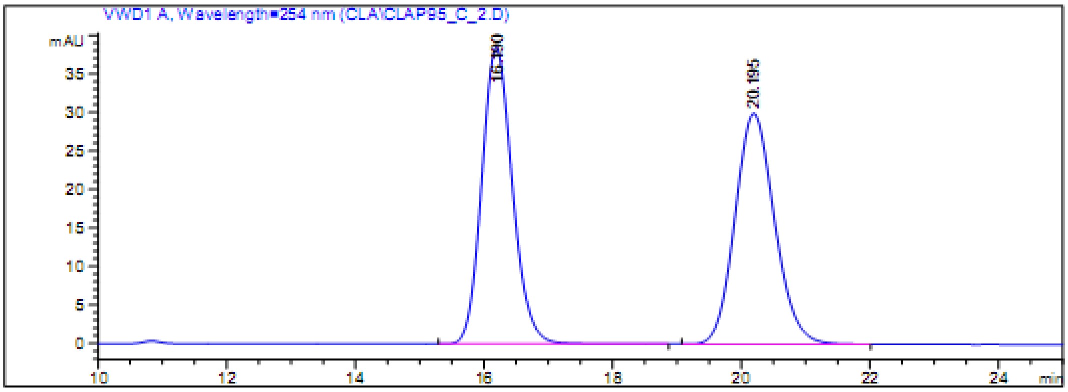

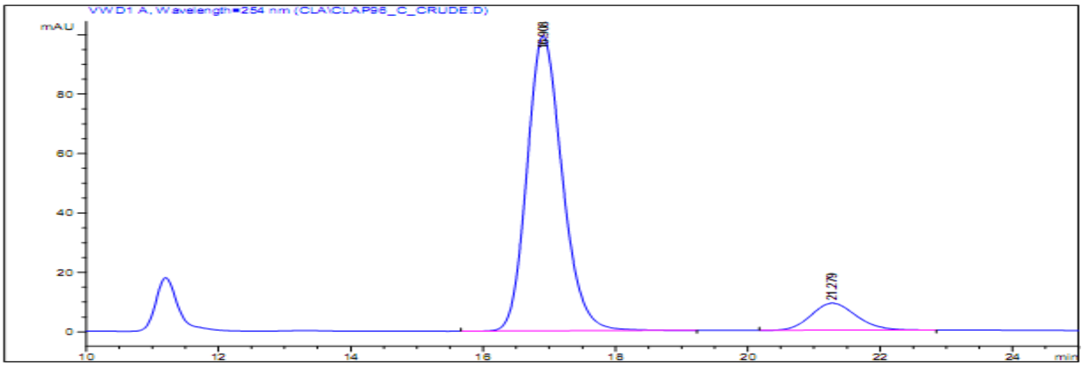

#	[min]		[min]	[mAU*s]	[mAU]	%
1	16.908	BB	0.5699	3673.81836	99.24155	89.5390
2	21.279	BB	0.7218	429.21817	9.07546	10.4610

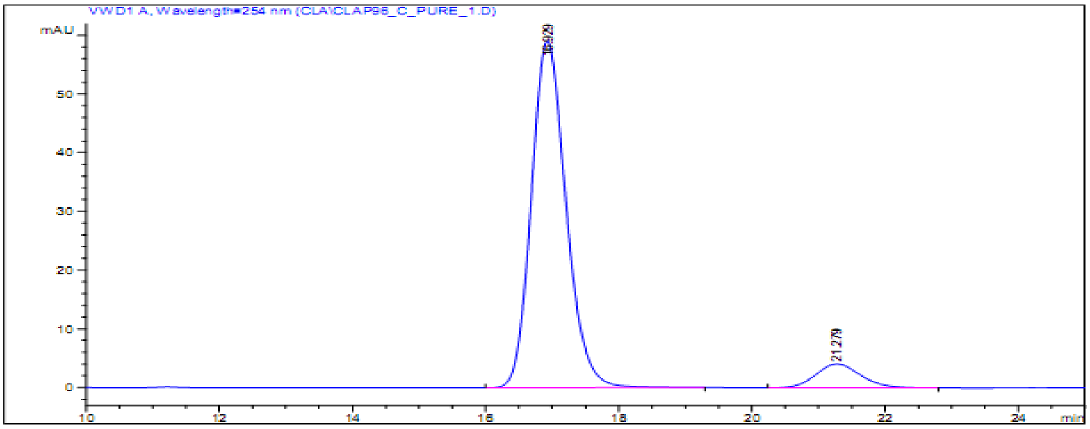

#	[min]		[min]	[mAU*s]	[mAU]	%
1	16.929	BB	0.5580	2136.63623	59.06593	91.8763
2	21.279	BB	0.7062	188.92233	4.03537	8.1237

Figure S18. HPLC traces of *rac*-**7d** (reference HPLC trace on the top) and (*R*)-**7d**. Area integration = 89.5:10.5 (second trace, 79% *ee* of crude (*R*)-**7d**, catalyzed by **Λ-Ir**) and 91.9:8.1 (third trace, 84% *ee* of isolated (*R*)-**7d**, catalyzed by **Λ-Ir**).

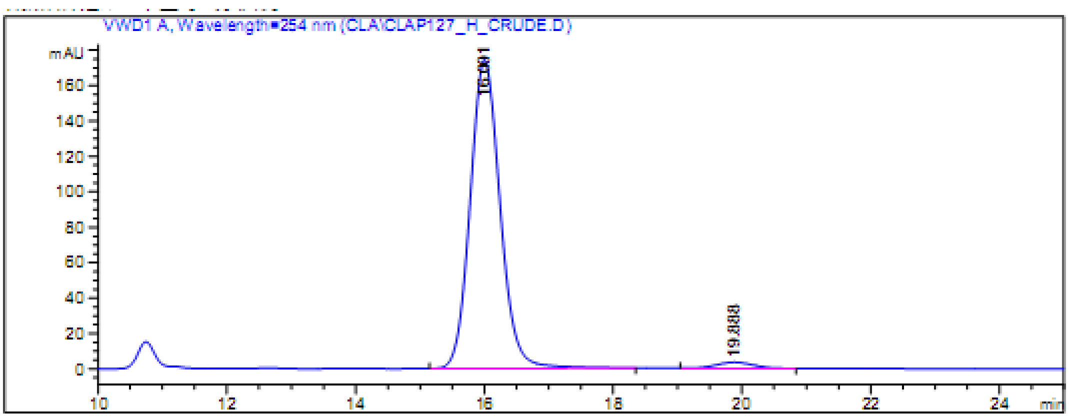

#	[min]		[min]	[mAU*s]	[mAU]	%
1	16.001	MM R	0.5378	5622.17480	174.22789	97.6297
2	19.888	MM R	0.6666	136.49632	3.41252	2.3703

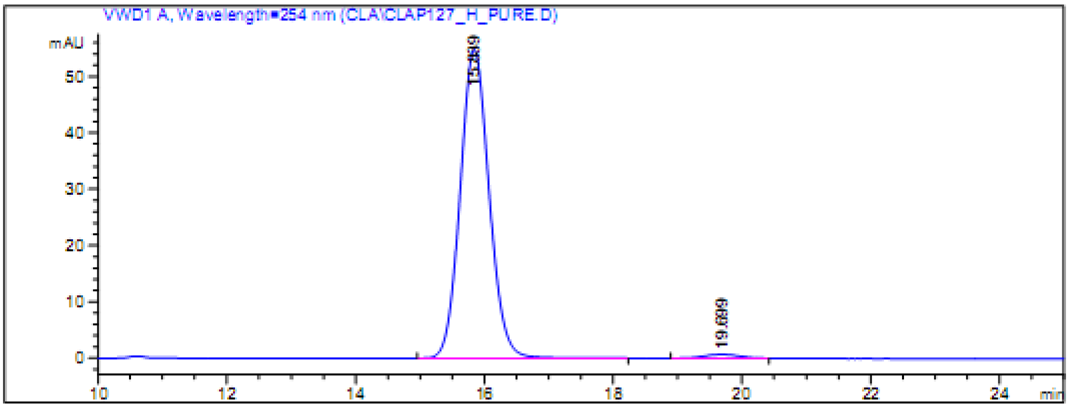

#	[min]		[min]	[mAU*s]	[mAU]	%
1	15.839	MM R	0.5214	1715.32739	54.83012	98.4391
2	19.699	MM R	0.6457	27.19997	7.02126e-1	1.5609

Figure S19. HPLC traces of (*R*)-**7d**. Area integration = 97.6:2.4 (first trace, 95% *ee* of crude (*R*)-**7d**, catalyzed by **Λ-Rh**) and 98.4:1.6 (second trace, 97% *ee* of isolated (*R*)-**7d**, catalyzed by **Λ-Rh**).

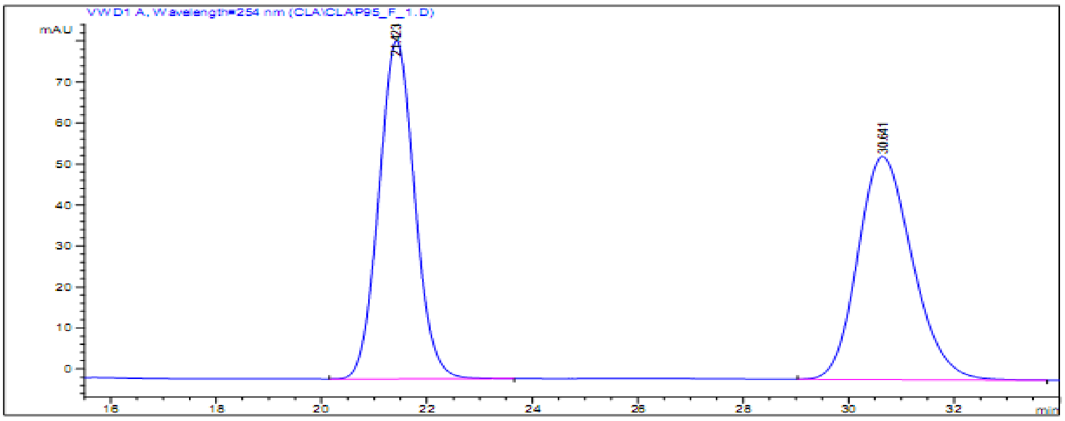

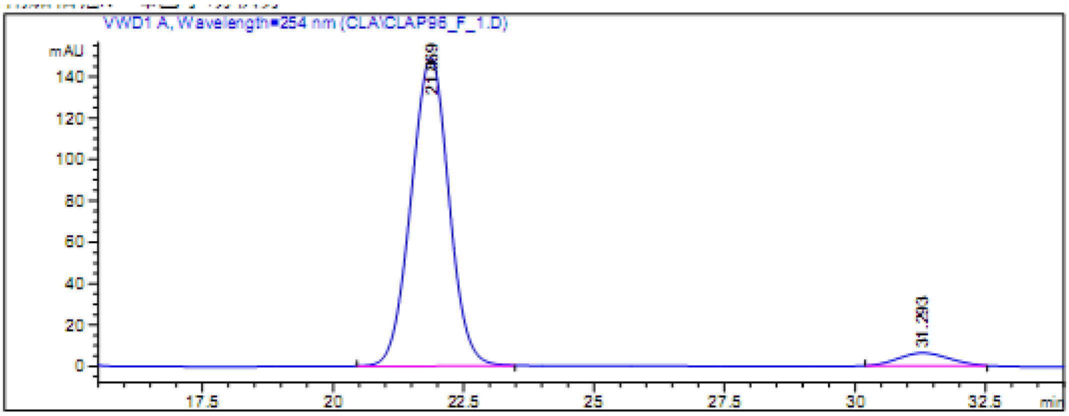

#	[min]		[min]	[mAU*s]	[mAU]	%
1	21.869	MM R	0.8417	7531.50635	149.13184	94.7839
2	31.293	MM R	1.1215	414.46735	6.15963	5.2161

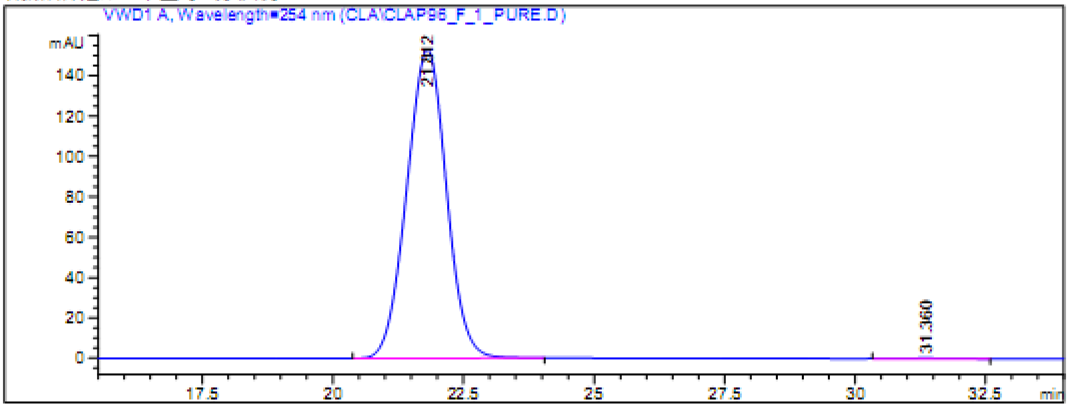

#	[min]		[min]	[mAU*s]	[mAU]	%
1	21.812	MM R	0.8813	8096.11719	153.10631	99.7964
2	31.360	MM R	1.1369	16.51513	2.42118e-1	0.2036

Figure S20. HPLC traces of *rac*-**7e** (reference HPLC trace on the top) and (*R*)-**7e**. Area integration = 94.8:5.2 (second trace, 90% *ee* of crude (*R*)-**7e**, catalyzed by **Λ-Ir**) and 99.8:0.2 (third trace, >99.5% *ee* of isolated (*R*)-**7e**, catalyzed by **Λ-Ir**).

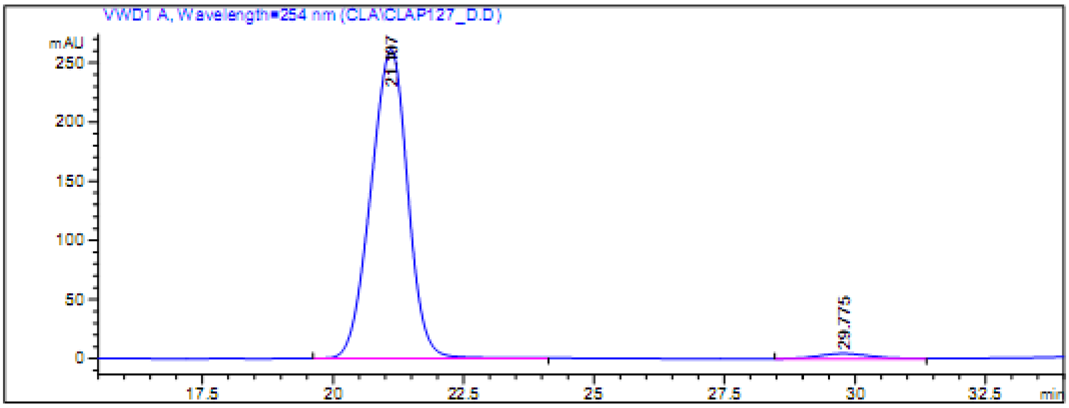

#	[min]		[min]	[mAU*s]	[mAU]	%
1	21.107	MM R	0.8185	1.28200e4	261.04633	97.8147
2	29.775	MM R	1.1207	286.41162	4.25923	2.1853

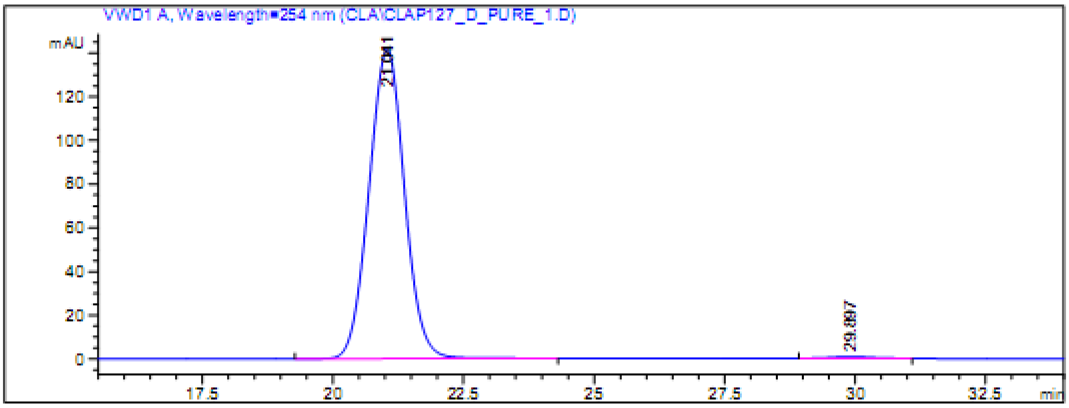

#	[min]		[min]	[mAU*s]	[mAU]	%
1	21.041	BB	0.7366	6701.76807	141.49170	99.2861
2	29.897	MM R	1.0530	48.18744	7.62726e-1	0.7139

Figure S21. HPLC traces of (*R*)-**7e**. Area integration = 97.8:2.2 (first trace, 96% *ee* of crude (*R*)-**7e**, catalyzed by **Λ-Rh**) and 99.3:0.7 (second trace, 99% *ee* of isolated (*R*)-**7e**, catalyzed by **Λ-Rh**).

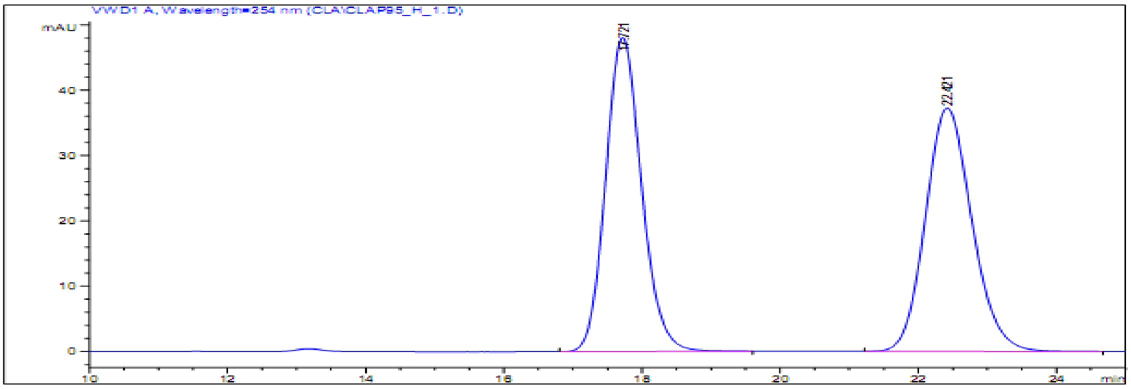

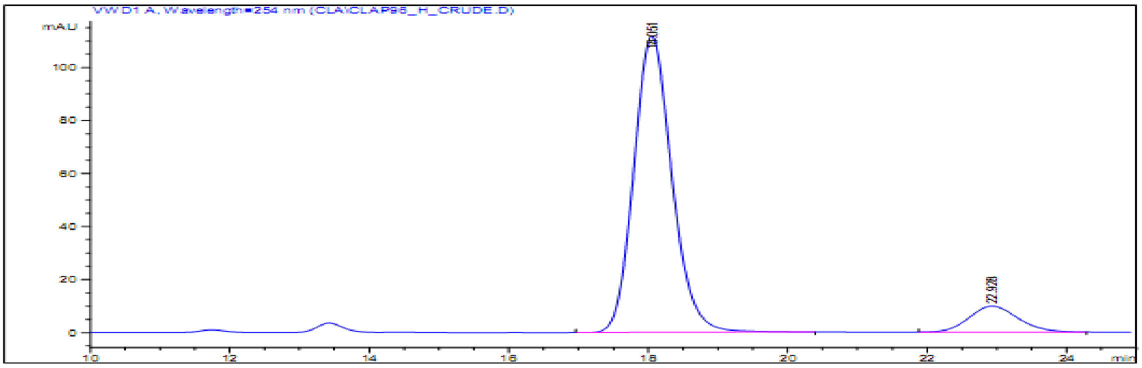

#	[min]		[min]	[mAU*s]	[mAU]	%
1	18.051	BB	0.5852	4245.28027	111.74877	89.9738
2	22.928	MM R	0.8105	473.07187	9.72775	10.0262

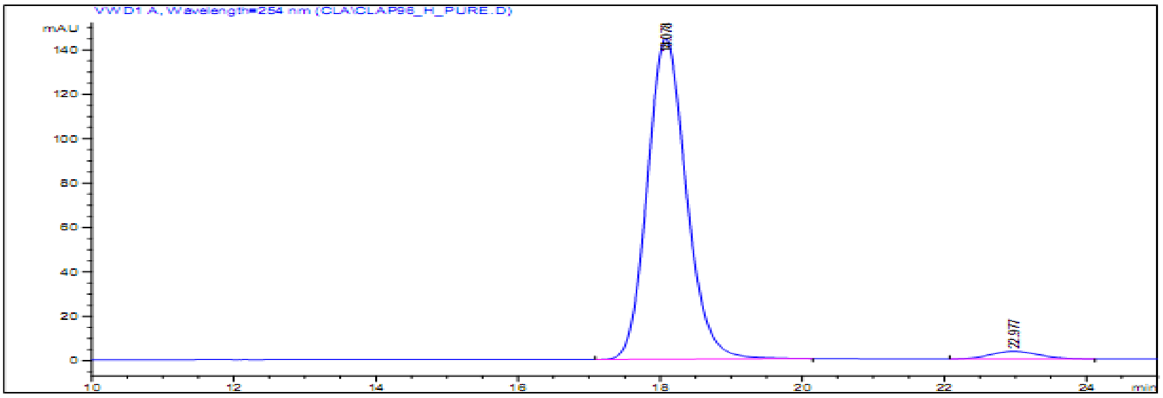

#	[min]		[min]	[mAU*s]	[mAU]	%
1	18.078	MM R	0.6336	5486.10449	144.32080	97.1995
2	22.977	MM R	0.7994	158.06741	3.29569	2.8005

Figure S22. HPLC traces of *rac*-**7f** (reference HPLC trace on the top) and (*R*)-**7f**. Area integration = 90:10 (second trace, 80% *ee* of crude (*R*)-**7f**, catalyzed by **Λ-Ir**) and 97.2:2.8 (third trace, 94% *ee* of isolated (*R*)-**7f**, catalyzed by **Λ-Ir**).

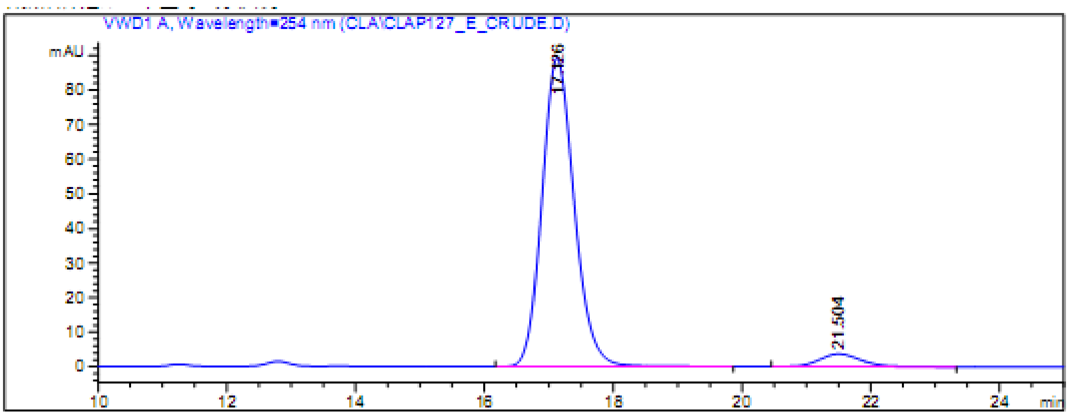

#	[min]		[min]	[mAU*s]	[mAU]	%
1	17.126	BB	0.5363	3093.38208	89.25340	95.0397
2	21.504	BB	0.6844	161.44954	3.64725	4.9603

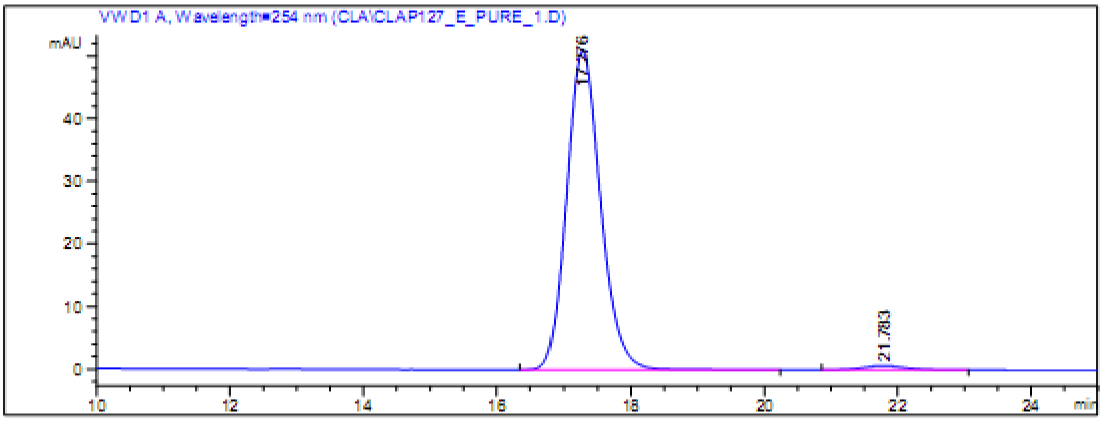

#	[min]		[min]	[mAU*s]	[mAU]	%
1	17.276	BB	0.5444	1798.06470	50.86566	98.4833
2	21.783	BB	0.6346	27.69135	6.18224e-1	1.5167

Figure S23. HPLC traces of (*R*)-**7f**. Area integration = 95:5 (first trace, 90% *ee* of crude (*R*)-**7f**, catalyzed by **Λ-Rh**) and 98.5:1.5 (second trace, 97% *ee* of isolated (*R*)-**7f**, catalyzed by **Λ-Rh**).

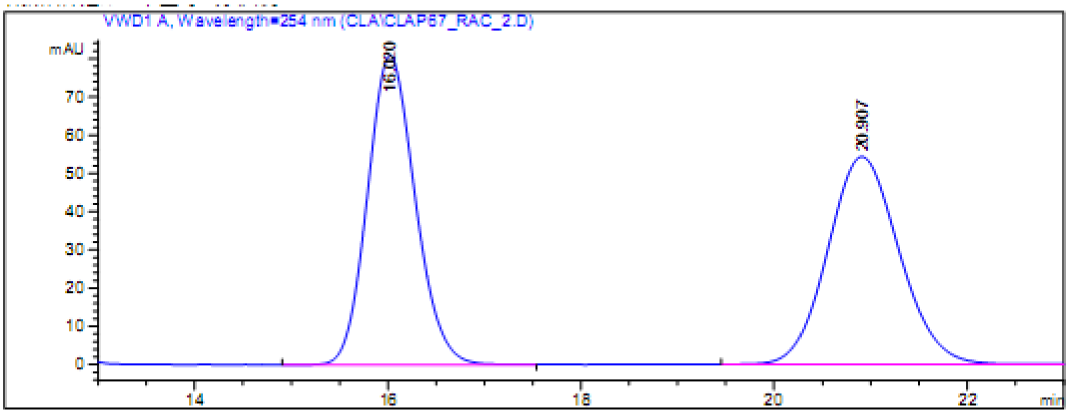

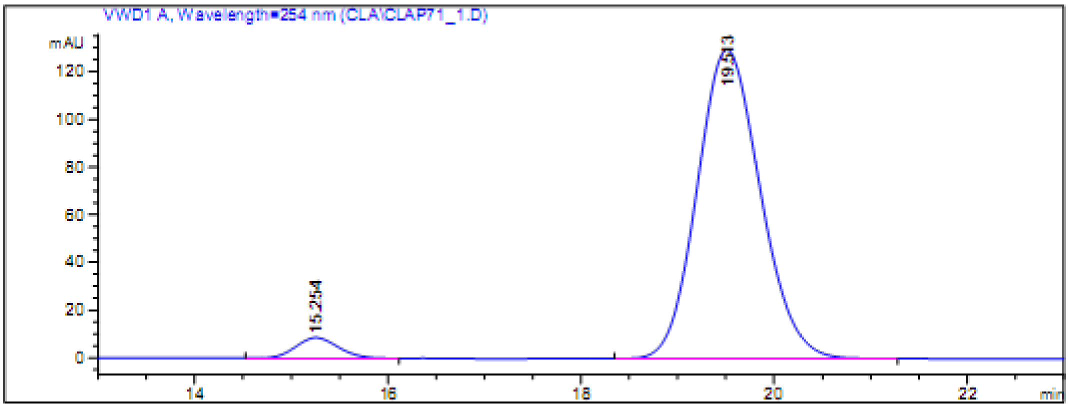

#	[min]		[min]	[mAU*s]	[mAU]	%
1	15.254	BB	0.4802	267.38327	8.65926	4.3499
2	19.513	MM R	0.7565	5879.52295	129.54134	95.6501

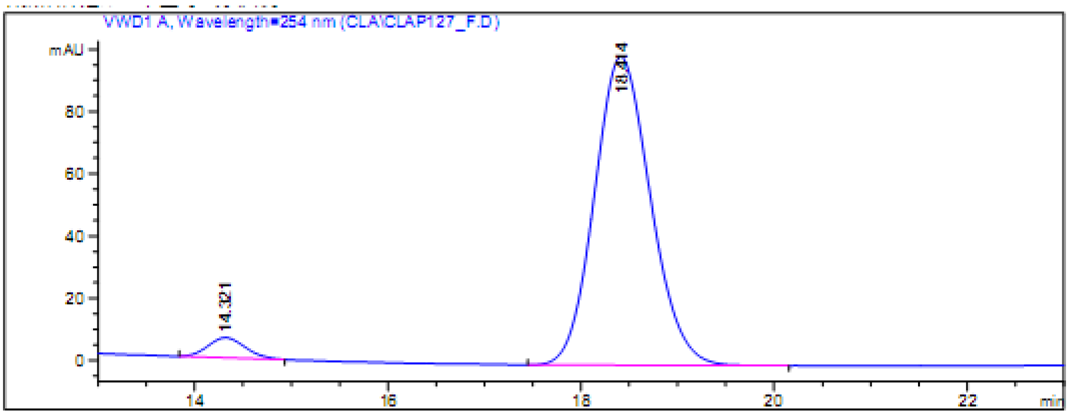

#	[min]		[min]	[mAU*s]	[mAU]	%
1	14.321	MM R	0.4459	171.94260	6.42729	4.1160
2	18.414	BB	0.6269	4005.52246	99.22857	95.8840

Figure S24. HPLC traces of *rac*-**7g** (reference HPLC trace on the top) and (*R*)-**7g**. Area integration = 95.7:4.3 (second trace, 91% *ee* of isolatd (*R*)-**7g**, catalyzed by **Λ-Ir**) and 95.9:4.1 (third trace, 92% *ee* of isolated (*R*)-**7g**, catalyzed by **Λ-Rh**).

6. Investigation of the Proposed Catalyst-Coordinated Substrate

To a solution of Δ-**Rh** (10.0 mg, 0.012 mmol) in CD_2Cl_2 (0.70 mL) at room temperature was added substrate **4** (9.5 mg, 0.063 mmol). The mixture was stirred at room temperature for 20 min and then analyzed by ^{1}H NMR spectroscopy. The ^{1}H NMR analysis is consistent with a fast bidentate coordination of **4** to Δ-**Rh** under release of the coordinated acetonitrile ligands.

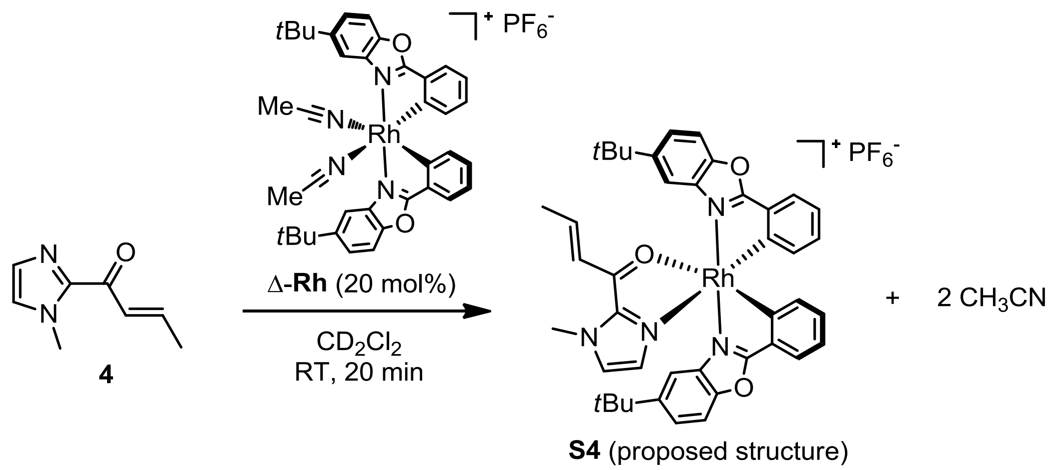

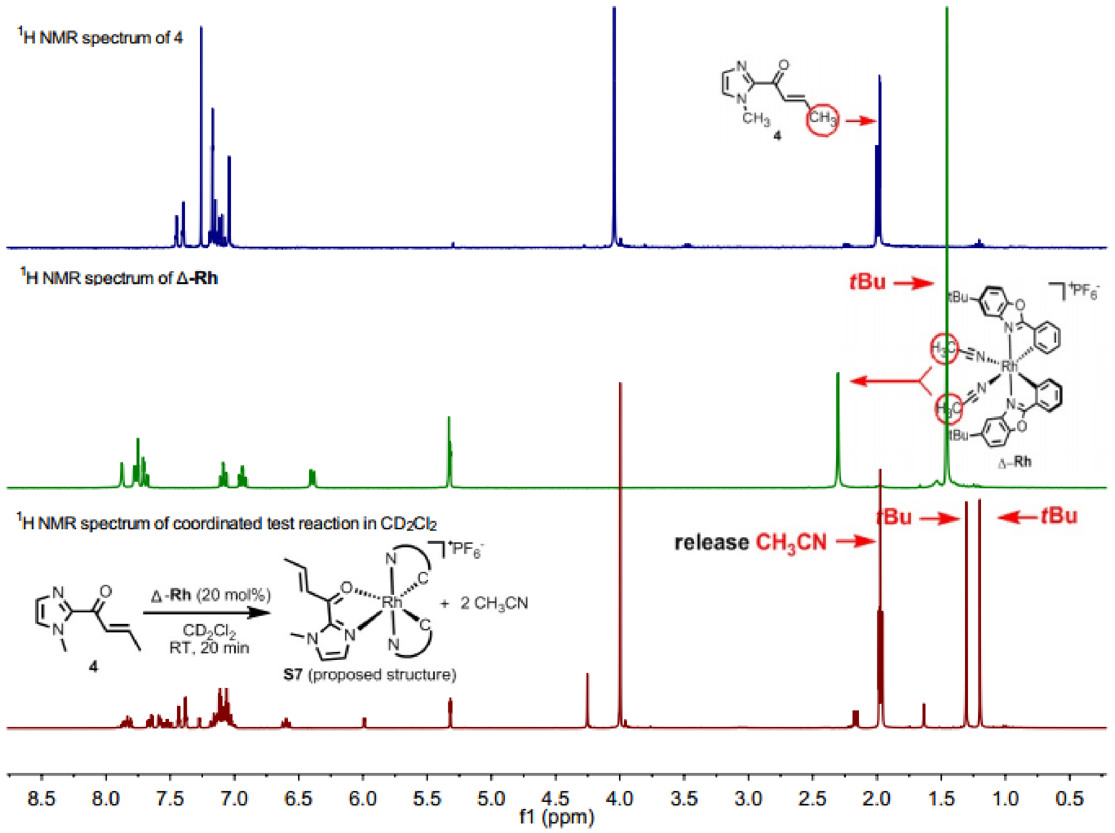

Figure S25. ^{1}H NMR spectra of acylimidazole substrate **4**, catalyst Δ-**Rh**, and a mixture of **4** and Δ-**Rh**.

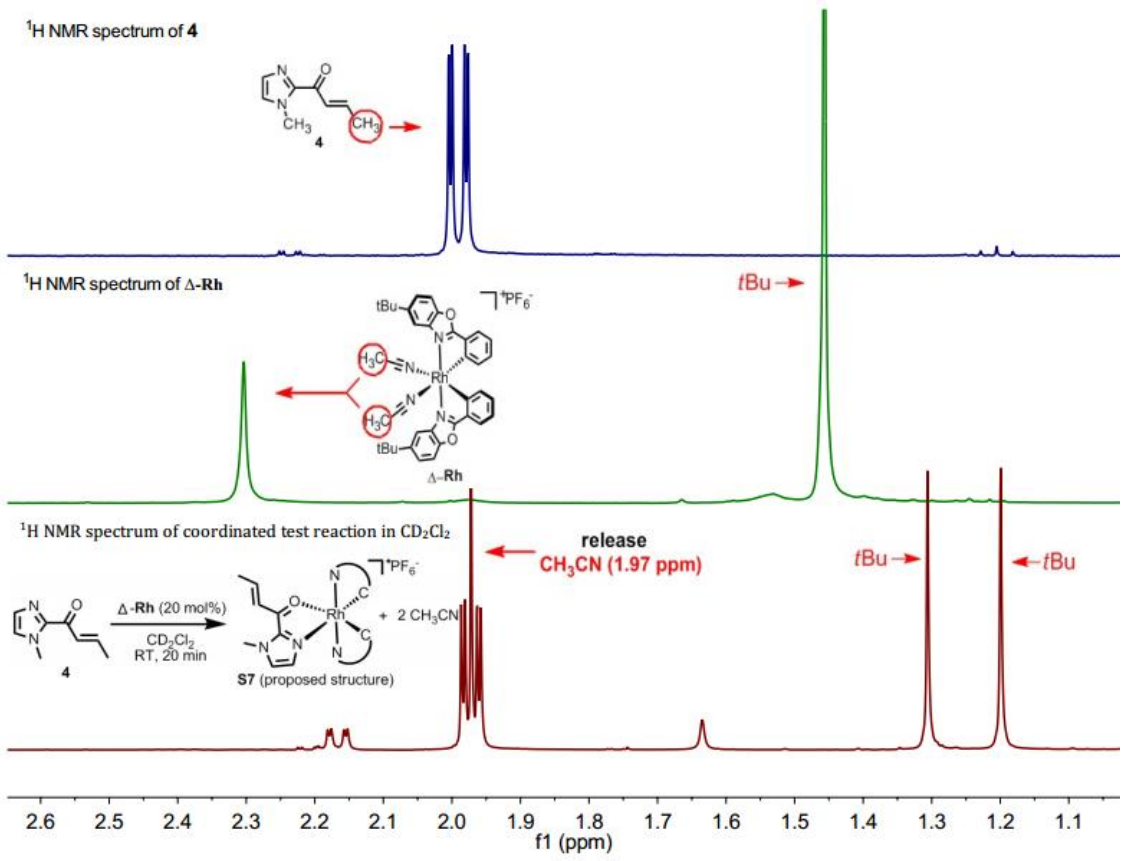

Figure S26. Excerpts of the ^{1}H NMR spectra from Figure S25.

7. Investigation of the Configurational Stability of the Rhodium Catalyst

7.1. Catalyst Stability Investigated by ^{1}H NMR

The rhodium complex Δ-**Rh** (5.0 mg) was dissolved in CD_2Cl_2 and kept in the NMR tube at room temperature under reduced light. ^{1}H NMR spectra were recorded after 2, 4, 6 and 8 days.

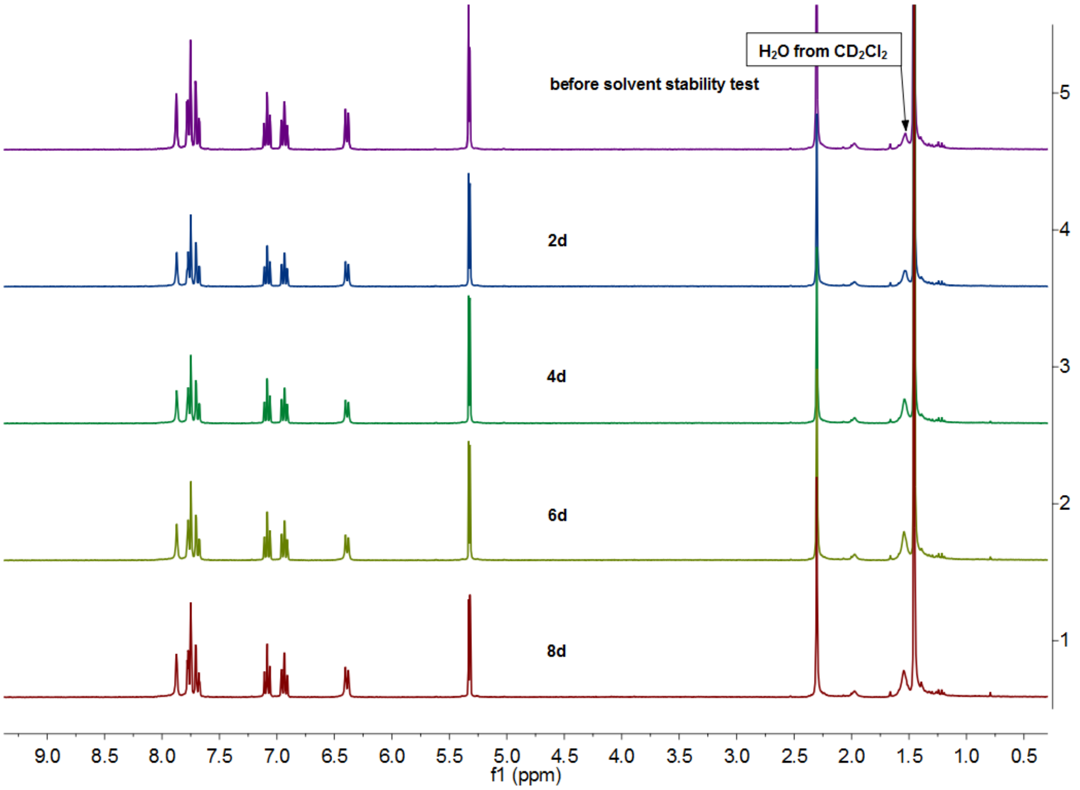

Figure S27. ^{1}H NMR of Δ-**Rh** recorded in CD_2Cl_2 over 8 days.

7.2. Catalyst Stability Investigated by HPLC on Chiral Stationary Phase

Enantiopure pure rhodium complex Λ-**Rh** (2.0 mg) was dissolved in CH_2Cl_2 (1.0 mL, HPLC grade) and kept in a brown glass vial at room temperature. The HPLC spectra were collected after 2-8 days. HPLC conditions: Daicel Chiralpak IB (250 × 4.6 mm) HPLC column, the column temperature was 25 °C and UV-absorption was measured at 254 nm. Solvent A = 0.1% TFA, solvent B = MeCN with a linear gradient of 30% to 41% B in 60 min at a flow rate = 0.6 mL/min.

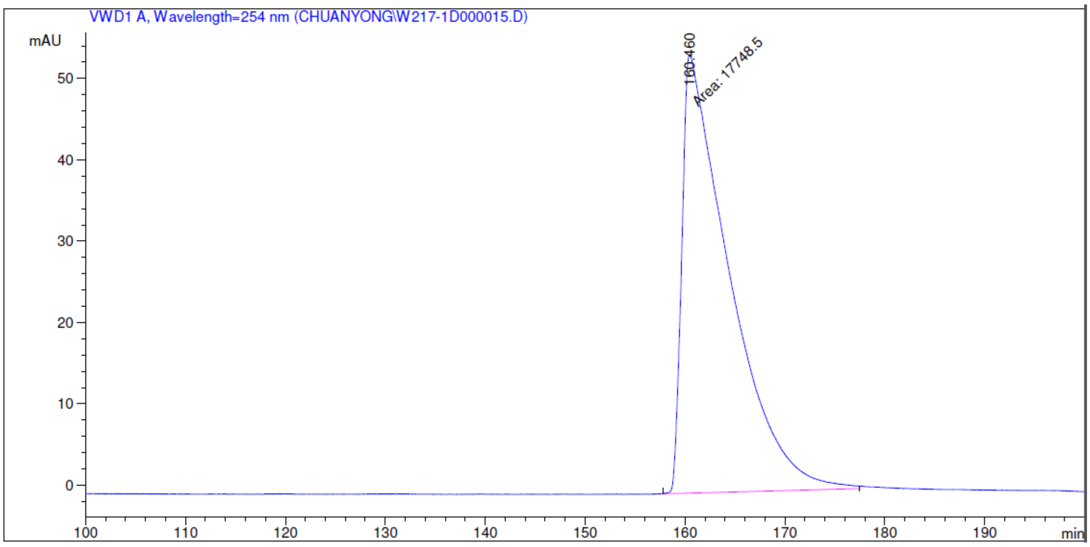

Figure S28. HPLC trace of the freshly prepared Λ-**Rh** in CH_2Cl_2 (>99% ee).

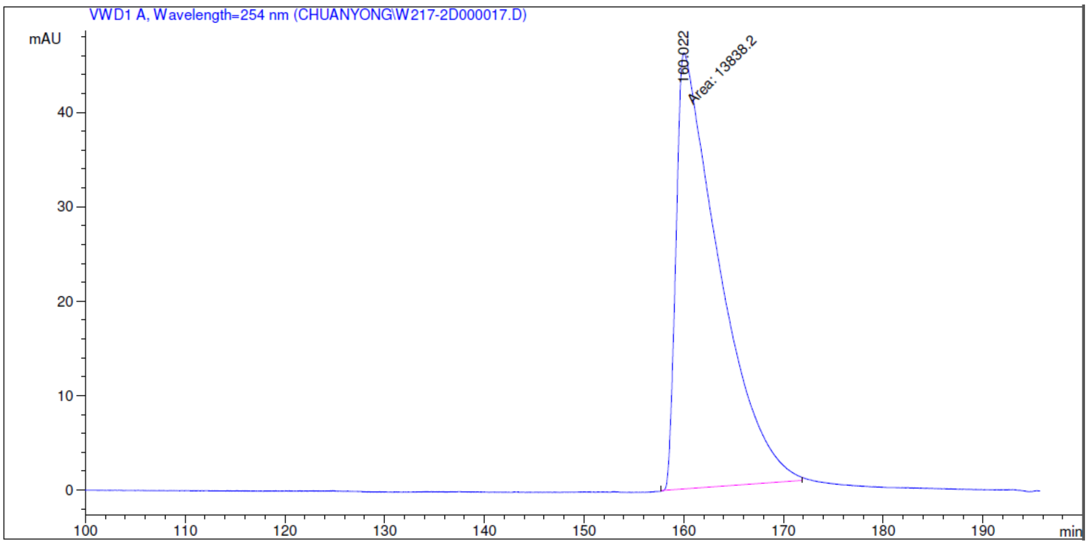

Figure S29. HPLC trace after 2 days in CH_2Cl_2 (>99% ee).

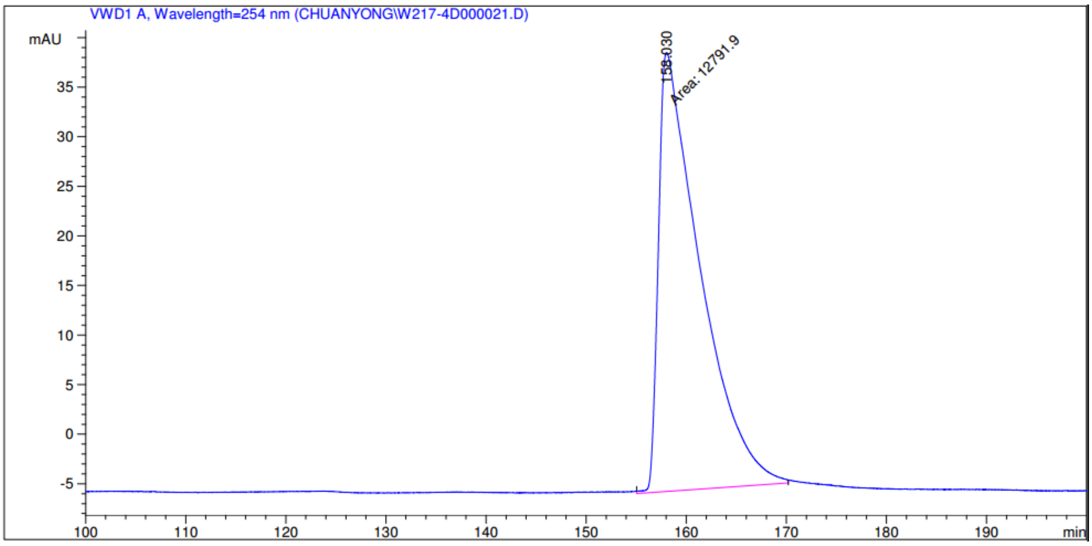

Figure S30. HPLC trace after 4 days in CH_2Cl_2 (>99% ee).

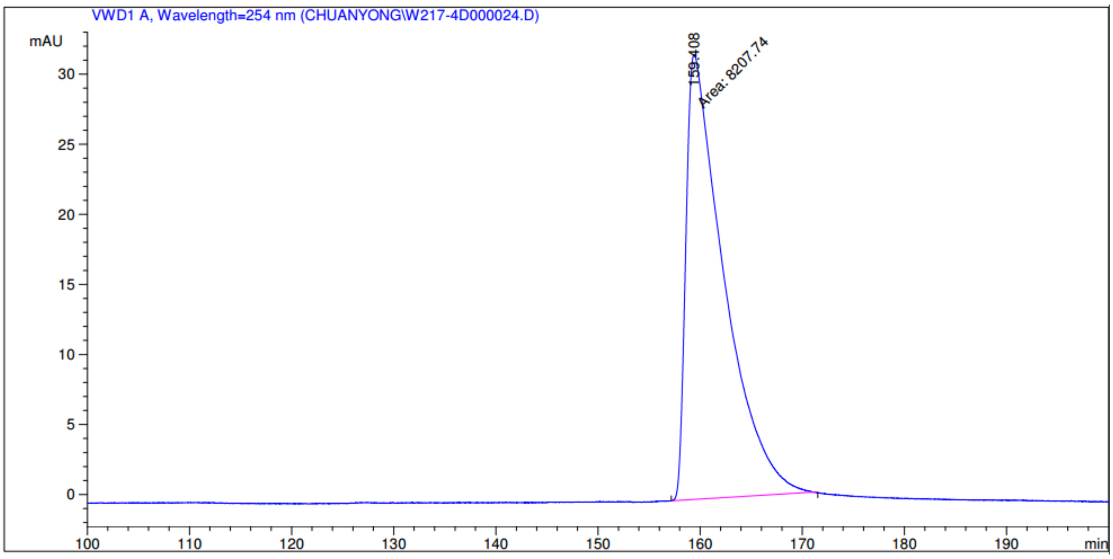

Figure S31. HPLC trace after 6 days in CH_2Cl_2 (>99% ee).

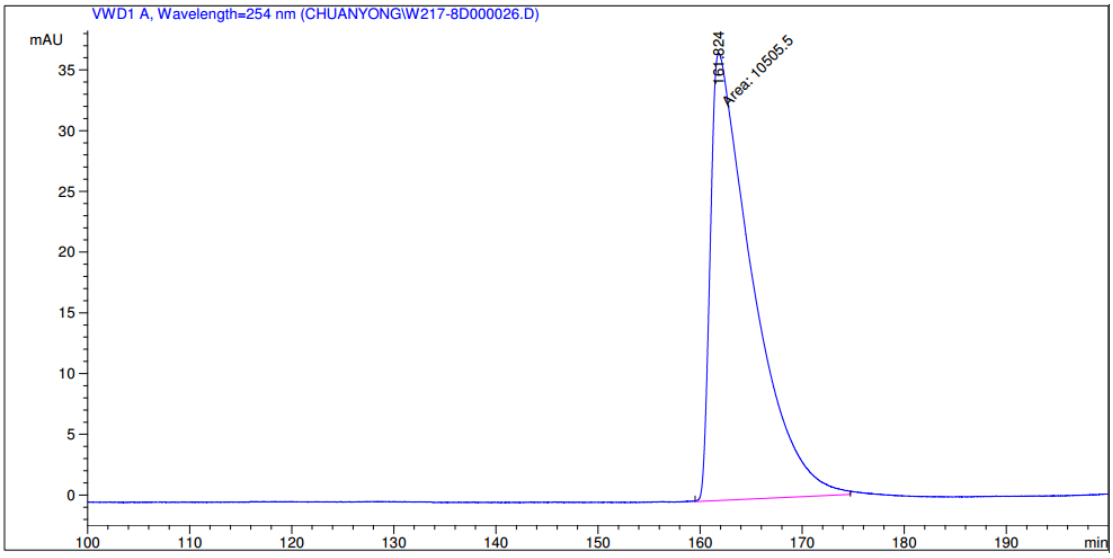

Figure S32. HPLC trace after 8 days in CH_2Cl_2 (>99% ee).

8. NMR Spectra of New Rhodium Complexes

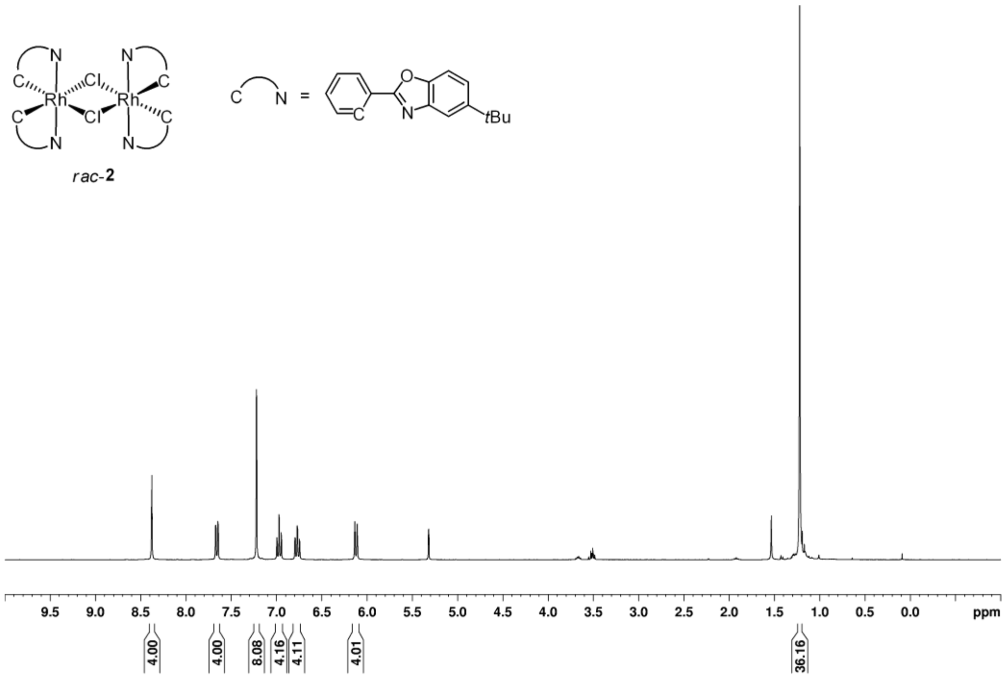

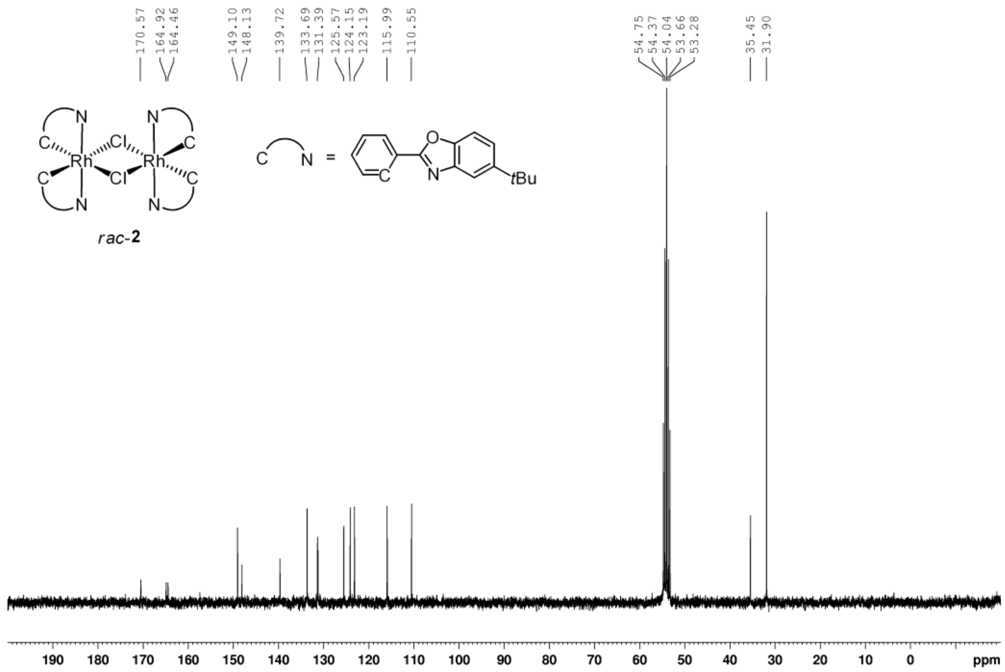

Figure S33. ^{1}H NMR and ^{13}C NMR spectrum of *rac*-**2**.

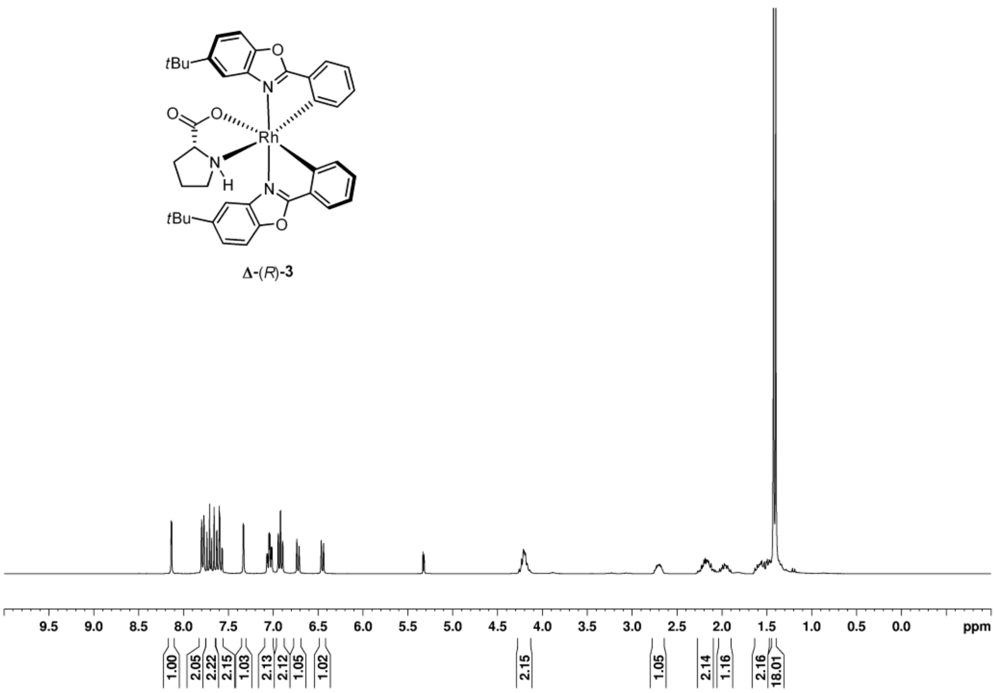

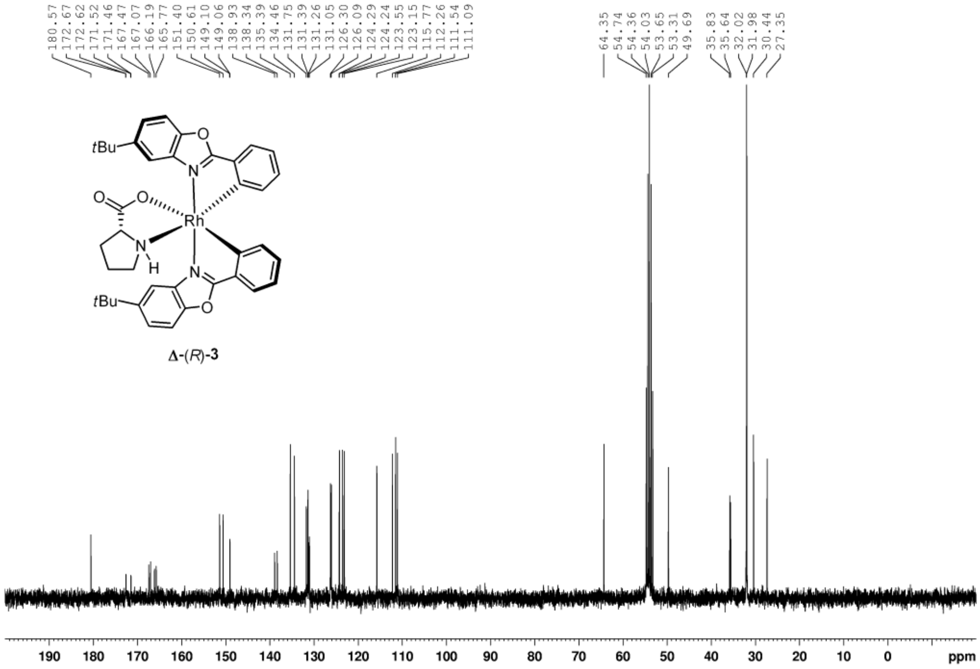

Figure S34. ^{1}H NMR and ^{13}C NMR spectrum of Δ-(*R*)-**3**.

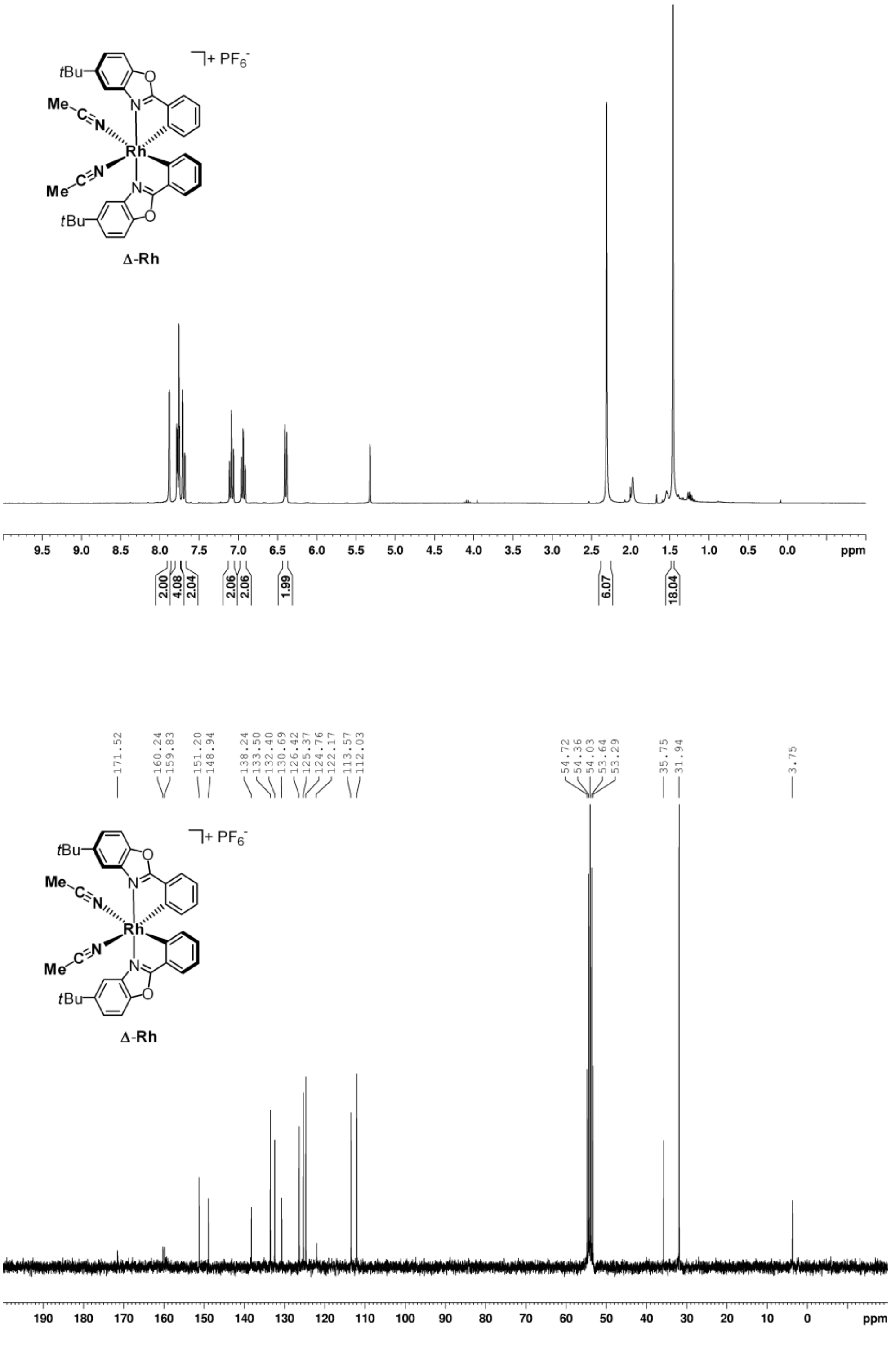

Figure S35. ^{1}H NMR and ^{13}C NMR spectrum of Δ-**Rh**.

9. Single Crystal X-Ray Diffraction

Crystals of Δ-(*R*)-**3** and Δ-**Rh** were obtained by slow diffusion from a solution of the compounds in CH_2Cl_2 layered with Et_2O at room temperature for several weeks. Crystals of the *R*-enantiomer of **5d** and racemic **5f** were obtained by slow diffusion from a solution of the compounds in CH_2Cl_2 layered with hexane at 5 °C for several days. Single crystals suitable for X-ray diffraction of the substrate coordinated rhodium catalyst (here denoted as **Rh1**) were obtained by reacting **4'** (0.060 mmol) with Δ/Λ-**Rh** (0.060 mmol) overnight at room temperature in CH_2Cl_2 (2.0 mL). After the slow addition of *n*-hexane (5.0 mL), crystals were collected after several days (70% yield). Single crystals of the iridium enolate complex were obtained as follows: To a solution of the racemic iridium catalyst (46.0 mg, 0.050 mmol) in CH_2Cl_2 (1.0 mL) was added the substrate **6a** (15.0 mg, 0.075 mmol). After 2 hours, the reaction was concentrated and purified by chromatography over silica gel using CH_2Cl_2/EtOAc (50:1), affording a pale yellow solid (42.8 mg, 0.048 mmol). Single crystals suitable for X-ray diffraction were obtained by slow diffusion from a solution of the compound in CH_2Cl_2 layered with *n*-hexane at room temperature for several days.

Crystal data and details of the structure determination are presented in Table S1. X-ray data were collected with a Bruker 3 circuit D8 Quest diffractometer with MoKα radiation (microfocus tube with multilayer optics) and Photon 100 CMOS detector. Scaling and absorption correction was performed by using the SADABS[13] software package of Bruker. Structures were solved using direct methods in SHELXS-97[14] and refined using the full matrix least squares procedure in SHELXL-2013[15]. The hydrogen atoms were placed in calculated positions and refined as riding on their respective C atom, and Uiso(H) was set at 1.2 Ueq(Csp^2) and 1.5 Ueq(Csp^3). Disorder of PF_6 ions, solvent molecules or methylene groups was refined using restraints for both the geometry and the anisotropic displacement factors. In the packing of **Rh1** there are holes present that contain diffuse electron density that may belong to heavily disordered solvent molecules. This was taken into account using the “squeeze” procedure in the PLATON[16] program system. The determination of the absolute configuration of the light atom structure **5d** by means of refining the “Flack parameter”[17] was not possible. The absolute configurations of compounds Δ-(*R*)-**3**, Δ-**Rh**, and the absolute structure of **Rh1** have been determined.[17] CCDC 1027144-1027147 and 1014508 contain the supplementary crystallographic data for this paper. These data can be obtained free of charge from The Cambridge Crystallographic Data Centre via www.ccdc.cam.ac.uk/data_request/cif.

Table S1. Crystal data and details of the structure determination.

Metal Complexes:

	Δ-(*R*)-**3**	**Δ-Rh**	**Rh1**	**Ir-enolate complex**
Empiric formula	$2(C_{39}H_{40}N_3O_4Rh)$ $\cdot 3(CH_2Cl_2)$	$C_{38}H_{38}N_4O_2Rh \cdot F_6P$ $\cdot CH_2Cl_2$	$C_{49}H_{48}N_4O_3Rh \cdot F_6P$ $\cdot 2(CH_2Cl_2)$	$C_{46}H_{43}IrN_4O_3$ $\cdot CH_2Cl_2$
Formula weight	1690.07	915.53	1158.64	976.97
Crystal system, space group	Orthorhombic, $P2_12_12_1$	Orthorhombic, $P2_12_12_1$	Orthorhombic, $Pna2_1$	Monoclinic, $C2/c$
a, *b*, *c* (Å)	13.919 (6), 19.144 (9), 28.616 (11)	13.1445 (5), 13.6427 (6), 22.5166 (8)	17.7761 (6), 22.9437 (8), 13.1111 (4)	38.9286(14), 13.3583(5), 17.0015(7)
α, β, γ (°)	90, 90, 90	90, 90, 90	90, 90, 90	90, 111.4793(12), 90
V (Å^3)	7625 (6)	4037.8 (3)	5347.4 (3)	8227.1(5)
Z	4	4	4	8
μ (mm^{-1})	0.70	0.66	0.62	3.423
Crystal size (mm)	0.44 × 0.07 × 0.06	0.51 × 0.24 × 0.17	0.26 × 0.06 × 0.03	0.13 x 0.07 x 0.02
T_{min}, T_{max}	0.76, 0.96	0.75, 0.90	0.88, 0.98	0.77, 0.94
No. of measured, independent and observed [$I > 2\sigma(I)$] reflections	64188, 14187, 12148	39009, 9272, 8540	56957, 9643, 8691	86572, 7482, 6105
R_{int}	0.064	0.042	0.050	0.087
$(\sin \theta/\lambda)_{max}$ (Å^{-1})	0.606	0.650	0.602	0.601
$R[F^2 > 2\sigma(F^2)]$, $wR(F^2)$, S	0.036, 0.068, 1.02	0.029, 0.058, 1.03	0.035, 0.089, 1.03	0.030, 0.064, 1.04
No. of reflections	14187	9272	9643	7482
No. of parameters	996	590	705	568
No. of restraints	114	168	106	68
$\Delta\rho_{max}$, $\Delta\rho_{min}$ (e Å^{-3})	0.57, -0.43	0.51, -0.40	1.12, -0.51	0.85, -0.91
Absolute structure parameter	-0.020 (10)	-0.033 (8)	-0.015 (8)	-
CCDC no.	1027144	1027145	1028075	1014508

Organic Reaction Products:

	**5d**	**5f**
Empiric formula	$C_{14}H_{18}N_2O_5$	$C_{22}H_{26}N_2O_4$
Formula weight	294.30	382.45
Crystal system, space group	Orthorhombic, $P2_12_12_1$	Monoclinic, $P2_1/c$
a, b, c (Å)	5.4571 (2), 15.3289 (6), 17.4084 (9)	10.1905 (5), 11.5731 (6), 17.4203 (8)
α, β, γ (°)	90, 90, 90	90, 97.5045 (16), 90
V (Å^3)	1456.24 (11)	2036.88 (17)
Z	4	4
μ (mm^{-1})	0.10	0.09
Crystal size (mm)	0.45 × 0.08 × 0.05	0.40 × 0.30 × 0.08
T_{min}, T_{max}	0.95, 0.99	0.93, 0.99
No. of measured, independent and observed [$I > 2\sigma(I)$] reflections	11088, 2707, 2478	47435, 4679, 3939
R_{int}	0.037	0.037
$(\sin \theta/\lambda)_{max}$ (Å^{-1})	0.605	0.650
$R[F^2 > 2\sigma(F^2)]$, $wR(F^2)$, S	0.029, 0.067, 1.05	0.038, 0.114, 1.12
No. of reflections	2707	4679
No. of parameters	194	258
No. of restraints	0	0
$\Delta\rho_{max}$, $\Delta\rho_{min}$ (e Å^{-3})	0.15, -0.20	0.35, -0.35
Absolute structure parameter	0.3 (4)	–
CCDC no.	1027146	1027147

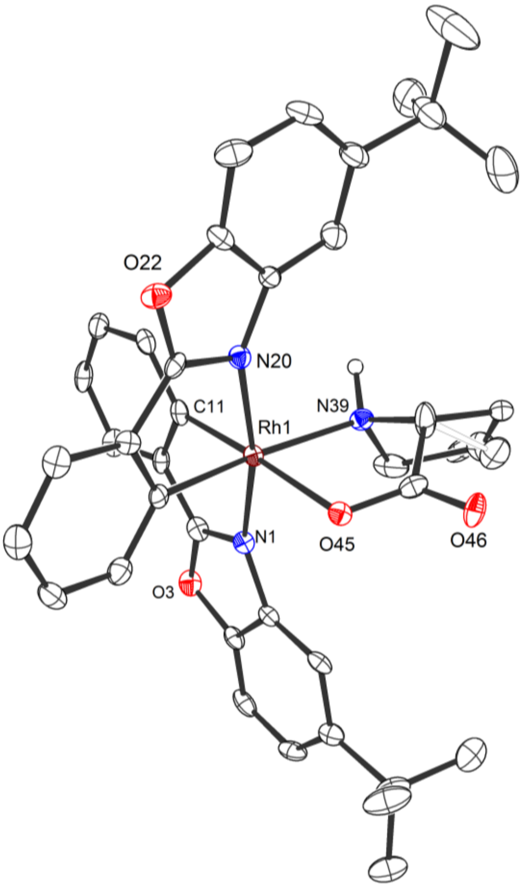

Figure S36. Crystal structure of Δ-(*R*)-**3**. ORTEP drawing with 50% probability thermal ellipsoids.

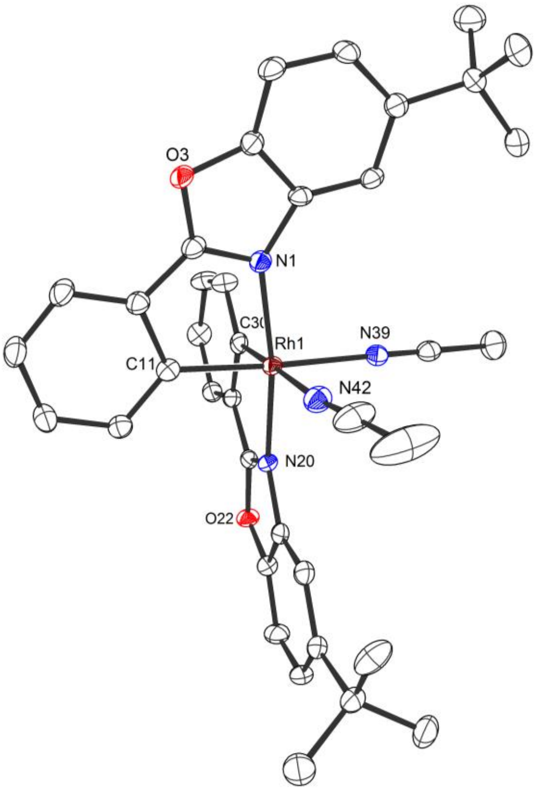

Figure S37. Crystal structure of Δ-**Rh**. ORTEP drawing with 50% probability thermal ellipsoids. The hexafluorophosphate counteranion is omitted for clarity.

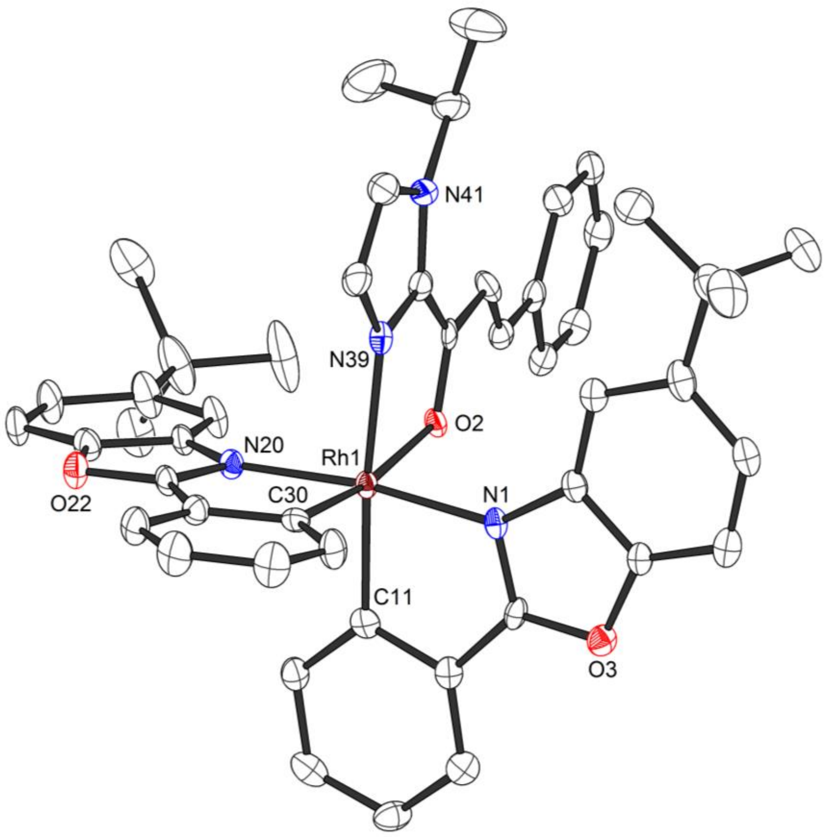

Figure S38. Crystal structure of racemic **Rh1**. ORTEP drawing with 50% probability thermal ellipsoids. The hexafluorophosphate counteranion is omitted for clarity.

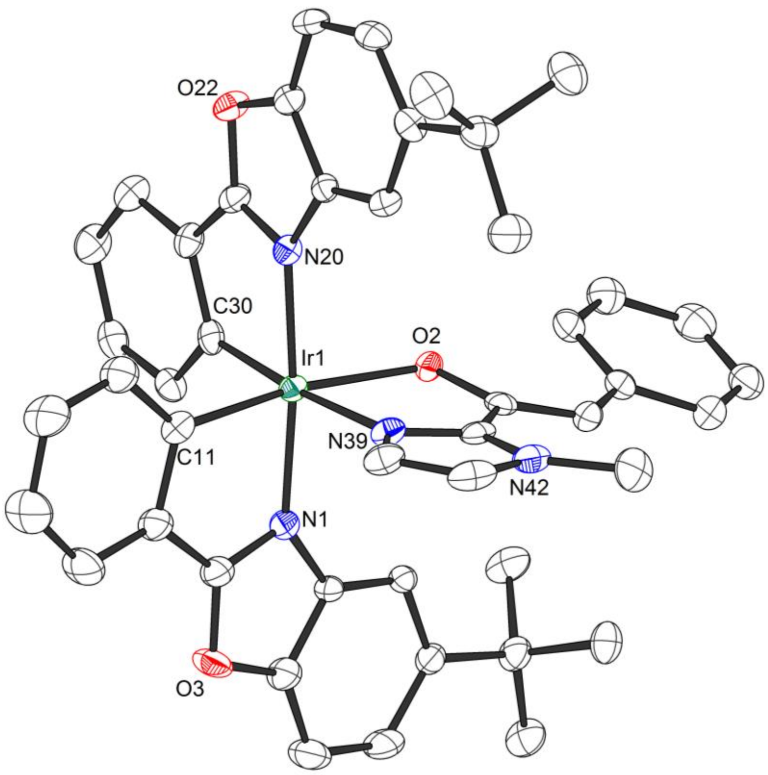

Figure S39. Crystal structure of a racemic iridium enolate complex. ORTEP drawing with 50% probability thermal ellipsoids. The hexafluorophosphate counteranion is omitted for clarity.

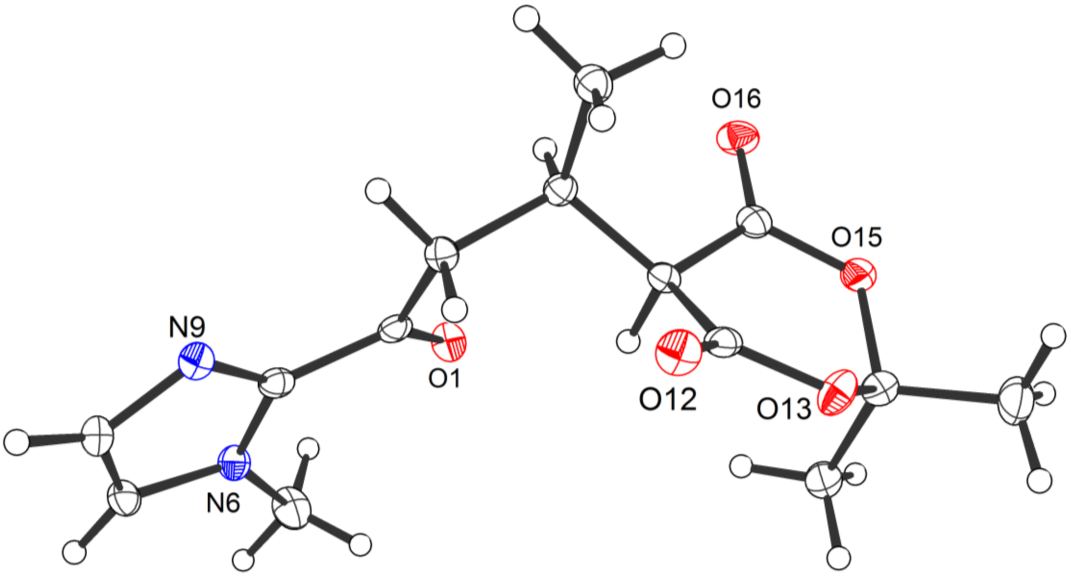

Figure S40. Crystal structure of **5d**. ORTEP drawing with 50% probability thermal ellipsoids.

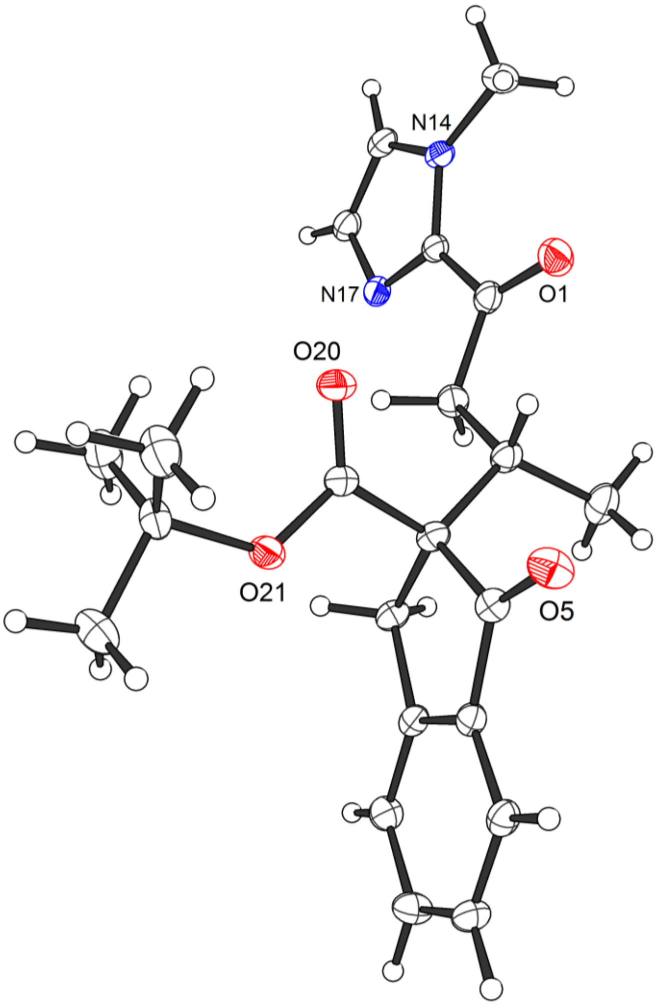

Figure S41. Crystal structure of racemic **5f** to verify the relative configuration. ORTEP drawing with 50% probability thermal ellipsoids.

10. References

1) D. A. Evans, K. R. Fandrick, H. J. Song, *J. Am. Chem. Soc.* **2005**, *127*, 8942.

2) a.) T. A. Moss,D. R. Fenwick, D. J. Dixon, *J. Am. Chem. Soc.* **2008**, *130*, 10076; b.) Q. H. Deng, T. Bleith, H. Wadepohl, L. H. Gade, *J. Am. Chem. Soc.* **2013**, *135*, 535.

3) H. Huo, C. Fu, K. Harms, E. Meggers, *J. Am. Chem. Soc.* **2014**, *136*, 2990.

4) Y. Chen, L. Qian, W. Zhang, B. Han, *Angew. Chem. Int. Ed.* **2008**, *47*, 9330.

5) R. Xing, Y. Li, Q. Liu, Q. Meng, J. Li, X. Shen, Z. Liu, B. Zhou, X. Yao, Z. Liu, *Eur. J. Org. Chem.* **2010**, 6627.

6) D. Ramlot, M. Rebarz, L. Volker, M. Ovaere, D. Beljonne, W. Dehaen, L. Van Meervelt, C. Moucheron, A. Kirsch-De Mesmaeker, *Eur. J. Org. Chem.* **2013**, 2031.

7) R. Urban, R. Krämer, S. Mihan, K. Polborn, B. Wagner, W. Beck, *J. Organomet. Chem.* **1996**, *517*, 191.

8) Y. Li, C. Wang, G. Jia, S. Lu, C. Li, *Tetrahedron*, **2013**, *69*, 6585.

9) B. M. Trost, K. Lehr, D. J. Michaelis, J. Xu, A. K. Buckl, *J. Am. Chem. Soc.* **2010**, *135*, 8915.

10) a.) A. Msutu, R. Hunter, *Tetrahedron Lett.* **2014**, *55*, 2295; b.) C. Beattie, M. North, P. Villuendas, *Molecules*, **2011**, *16*, 3420.

11) D. A. Evans, K. R. Fandrick, *Org. Lett.* **2006**, *8*, 2249.

12) B. List, *J. Am. Chem. Soc.* **2002**, *124*, 5656.

13) SADABS 2012/1, Bruker AXS Inc., Madison, Wisconsin, USA, **2012**.

14) G. M. Sheldrick, *Acta Crystallogr., Sect. A* **2008**, *64*, 112.

15) SHELXL-2013, G. M. Sheldrick, University of Göttingen, Germany, **2013**.

16) A. L. Spek, *Acta Crystallogr., Sect. D* **2009**, *65*, 148.

17) S. Parsons, H. Flack, T. Wagner, *Acta Crystallogr., Sect. B* **2013**, *69*, 249.
